# Supplementary material for: Bacterial turbulence drives interfacial waves and shape dynamics in phase-separated droplets
Source: Nat Commun. 2026 Jul 14;17:8661. doi: 10.1038/s41467-026-75497-5 (PMC13490374; doi:10.1038/s41467-026-75497-5)
Supplement: Supplementary file 1 [file 41467_2026_75497_MOESM12_ESM.pdf]

nature portfolio

Corresponding author(s): Zhihong You; H. P. Zhang

Last updated by author(s): Jun 23, 2026

Reporting Summary

Nature Portfolio wishes to improve the reproducibility of the work that we publish. This form provides structure for consistency and transparency in reporting. For further information on Nature Portfolio policies, see our Editorial Policies and the Editorial Policy Checklist.

Statistics

For all statistical analyses, confirm that the following items are present in the figure legend, table legend, main text, or Methods section.

n/a	Confirmed	
☐	☒	The exact sample size (*n*) for each experimental group/condition, given as a discrete number and unit of measurement
☐	☒	A statement on whether measurements were taken from distinct samples or whether the same sample was measured repeatedly
☒	☐	The statistical test(s) used AND whether they are one- or two-sided<br>*Only common tests should be described solely by name; describe more complex techniques in the Methods section.*
☒	☐	A description of all covariates tested
☒	☐	A description of any assumptions or corrections, such as tests of normality and adjustment for multiple comparisons
☐	☒	A full description of the statistical parameters including central tendency (e.g. means) or other basic estimates (e.g. regression coefficient) AND variation (e.g. standard deviation) or associated estimates of uncertainty (e.g. confidence intervals)
☒	☐	For null hypothesis testing, the test statistic (e.g. *F*, *t*, *r*) with confidence intervals, effect sizes, degrees of freedom and *P* value noted<br>*Give P values as exact values whenever suitable.*
☒	☐	For Bayesian analysis, information on the choice of priors and Markov chain Monte Carlo settings
☒	☐	For hierarchical and complex designs, identification of the appropriate level for tests and full reporting of outcomes
☒	☐	Estimates of effect sizes (e.g. Cohen's *d*, Pearson's *r*), indicating how they were calculated

Our web collection on statistics for biologists contains articles on many of the points above.

Software and code

Policy information about availability of computer code

Data collection	Confocal microscopy data were collected using a Nikon spinning disk confocal microscope. Image acquisition and preprocessing were performed using Nikon NIS-Element Viewer.
Data analysis	Data analysis was performed using MATLAB .

For manuscripts utilizing custom algorithms or software that are central to the research but not yet described in published literature, software must be made available to editors and reviewers. We strongly encourage code deposition in a community repository (e.g. GitHub). See the Nature Portfolio guidelines for submitting code & software for further information.

Data

Policy information about availability of data

All manuscripts must include a data availability statement. This statement should provide the following information, where applicable:

- Accession codes, unique identifiers, or web links for publicly available datasets
- A description of any restrictions on data availability
- For clinical datasets or third party data, please ensure that the statement adheres to our policy

Source data underlying the graphs in the main figures and Supplementary Information are provided with this paper as Source Data files. Representative microscopy images and movies are provided in the main figures, Supplementary Information and Supplementary Movies.

Research involving human participants, their data, or biological material

Policy information about studies with human participants or human data. See also policy information about sex, gender (identity/presentation), and sexual orientation and race, ethnicity and racism.

Reporting on sex and gender	NA
Reporting on race, ethnicity, or other socially relevant groupings	NA
Population characteristics	NA
Recruitment	NA
Ethics oversight	NA

Note that full information on the approval of the study protocol must also be provided in the manuscript.

Field-specific reporting

Please select the one below that is the best fit for your research. If you are not sure, read the appropriate sections before making your selection.

- [x] Life sciences
- [ ] Behavioural & social sciences
- [ ] Ecological, evolutionary & environmental sciences

For a reference copy of the document with all sections, see nature.com/documents/nr-reporting-summary-flat.pdf

Life sciences study design

All studies must disclose on these points even when the disclosure is negative.

Sample size	No statistical method was used to predetermine sample size. The sample size for each analysis is reported in the corresponding figure legends, Methods or Supplementary Information. No statistical test was performed to determine sample size sufficiency. Our sample sizes are similar to those reported in previous publications.
Data exclusions	For analyses of time-resolved interfacial deformation and shape dynamics, droplets were excluded if they merged with other droplets before the required observation duration was reached, because after coalescence the radius of the droplet are changed.
Replication	Key experimental observations, including bacterial partitioning into dextran-rich droplets, activity-driven interfacial fluctuations, large droplet deformations, filament-like protrusions, contact-line deformation and accelerated coarsening, were reproduced across independently prepared samples. Quantitative results are reported from the independent droplets, movies or temporal segments specified in the figure legends, Supplementary Information and Source Data files.
Randomization	Randomization was not applicable because the experimental groups were defined by the prepared bacterial volume fractions.
Blinding	Blinding was not used because the bacterial-density conditions and droplet morphologies were visually apparent during imaging and analysis. Quantitative image analysis was performed using predefined procedures for contour extraction, flow-field analysis, fluctuation spectra and dynamic structure factors.

Reporting for specific materials, systems and methods

We require information from authors about some types of materials, experimental systems and methods used in many studies. Here, indicate whether each material, system or method listed is relevant to your study. If you are not sure if a list item applies to your research, read the appropriate section before selecting a response.

Materials & experimental systems

n/a	Involved in the study	
x		Antibodies
x		Eukaryotic cell lines
x		Palaeontology and archaeology
x		Animals and other organisms
x		Clinical data
x		Dual use research of concern
x		Plants

Methods

n/a	Involved in the study	
x		ChIP-seq
x		Flow cytometry
x		MRI-based neuroimaging

Plants

Seed stocks	NA
Novel plant genotypes	NA
Authentication	NA
