# Supplementary material for: Bacterial turbulence drives interfacial waves and shape dynamics in phase-separated droplets
Source: Nat Commun. 2026 Jul 14;17:8661. doi: 10.1038/s41467-026-75497-5 (PMC13490374; doi:10.1038/s41467-026-75497-5)
Supplement: Supplementary file 2 [file 41467_2026_75497_MOESM13_ESM.pdf]

nature portfolio

Peer Review File

Bacterial turbulence drives interfacial waves and shape dynamics in phase-separated droplets

Corresponding Author: Professor H. P. Zhang

This file contains all reviewer reports in order by version, followed by all author rebuttals in order by version.

Version 0:

Reviewer comments:

Reviewer #1

(Remarks to the Author)
This manuscript presents an original, visually compelling, and mechanistically insightful experimental-theoretical study of internally active liquid-liquid phase-separated (LLPS) droplets powered by motile bacteria. The platform is clever and minimal, bridging synthetic biology, active matter hydrodynamics, and non-equilibrium multiphase systems.

They found that in low-activity droplets, propagating interfacial waves via viscosity matching in low-Re regime, high-activity droplets sustained super-Plateau deformations with cell-sized filaments, enhanced motility, and dramatically accelerated coarsening and sedimentation.
These results are novel and well-supported by confocal imaging, PIV, structure factor analysis, TTSH + Cahn-Hilliard simulations. The "effective inertial response," arising from active stresses that counteract viscous damping, represents a novel hydrodynamic mechanism for generating underdamped dynamics in confined active multiphase fluids, distinct from director-mediated coupling in active nematics or localized forcing mechanisms in active vesicles reported in previous studies.

This work demonstrates clear significance for understanding activity-driven reprogramming of phase-separated systems (relevant to biomolecular condensates under cellular forces or programmable synthetic compartments). Overall, this is a strong and original contribution that is suitable for publication after minor revisions. The main text is generally clear and logically structured, but several points require clarification showed below.

1. Bacterial cells have a density slightly higher than water, With increasing bacterial volume fraction, whether the dextran-rich droplet phase becomes denser than the surrounding PEG-rich phase? In Fig. 1, the accelerated sedimentation (<10 min to chamber bottom) and coarsening are attributed primarily to activity-induced motility, flows, and enhanced coalescence. However, could buoyancy/density mismatch contribute significantly on the 10–30 min timescale? A control experiments that with non-motile or dead bacteria at the same φ_b would help disentangle these contributions ?
2. In Figure 2e–h (and related simulation results), droplet radii are given as R=12 and R=32 with no units, the same for velocity. Could the authors clarify the unit system for the simulations (and any conversion to physical scales) to ensure readers can directly compare with experiments?
3. Figure 3 caption refers to "simulations of a spherical (top) and flat (bottom) interface." Does the flat interface case correspond to the results shown in Fig. S14d–e?
4. the authors set $\nu_1 + \nu_2 \approx 0$ (viscosity matching) to achieve near-zero effective damping and underdamped waves. How is the magnitude of the negative active viscosity ν_2 specifically chosen? Is it purely phenomenological/tuned to match observed wave cutoff and frequencies, or is there literature/experimental support for its order of magnitude in bacterial suspensions? Prior works on bacterial "superfluids" report negative effective viscosity primarily at very low shear rates (near-zero or weak flows, e.g., in confined Couette or microfluidic rheometry), what is the typical shear rate in the droplet?
5. In Figure 4, aspect ratios exceed the classical Rayleigh-Plateau threshold \pi and are sustained for seconds, with excess surface area $\Delta \approx 0.6$ and formation of cell-sized filaments (bacteria nematically aligned along the axis). What is the dominant physical mechanism stabilizing these extreme, non-equilibrium shapes against capillary breakup?
6. Do the low-activity "effective inertial response" (propagating waves via viscosity matching) and high-activity "sustained super-Plateau deformations + filaments" share the same mechanism?
7. In the Discussion, to better highlight novelty, could the authors more explicitly contrast this with wave propagation

mechanisms in other active interface literature, such as nonreciprocal director-velocity coupling in active nematics, or contractility-driven modes in active vesicles?

(Remarks on code availability)

Reviewer #2

(Remarks to the Author)
Chang et al. investigate phase-separated PEG–dextran droplets that encapsulate motile E. coli and ask how active stresses generated by E. coli affect the dynamics and shape fluctuations of a closed liquid–liquid interface. By varying the bacterial volume fraction inside the dextran-rich phase, the authors report two distinct regimes. At lower bacterial volume fraction, droplets remain nearly spherical but display strong interfacial fluctuations, including a crossover in the static fluctuation spectrum from S(k) ~ k^{-2} at small k to S(k) ~ k^{-4} at larger k, together with finite-frequency peaks in the dynamic spectrum that are interpreted as wave-like interfacial modes. At higher bacterial volume fraction, droplets show much stronger deformations, tether-like protrusions, enhanced near-wall motility after sedimentation, and faster coarsening. The authors support these observations using a Cahn–Hilliard + Toner–Tu–Swift–Hohenberg continuum model and an analytical flat-interface treatment. Overall, the topic is timely and relevant for the active soft matter community. However, I am not convinced by the present interpretation, and I think the manuscript requires major revision before it can be considered further.
Here are some specific comments, which might be helpful for future submissions:
1. The central claim that increased bacterial concentration corresponds to increased activity is not convincing. Increasing concentration is not equivalent to increasing activity at the level of individual bacteria. Activity should be defined through quantities such as swimming speed, persistence, or a suitable dimensionless activity parameter like Pe number. In contrast, increasing concentration mainly changes collision frequency, crowding, hydrodynamic coupling, and the onset of collective motion. The manuscript currently conflates these effects.

2. Related to this, without resolving the bacterial dynamics more carefully, it is difficult to interpret the physical state of the system at different concentrations. At high bacterial volume fraction, are the bacteria still individually motile, or is the system dominated by collective motion, crowding, or even partial kinetic arrest? The manuscript uses terms such as collective flow and active turbulence, but these claims are not sufficiently supported by direct characterization of bacterial motion.
3. I could not find a clear quantification of the bacterial swimming speed, even in the dilute limit. This is an important missing control. If the authors want to compare two concentrations and interpret them in terms of activity, they should at least report the individual bacterial speed distributions and how these change with concentration and time.
4. It is also not clear whether the high-concentration state corresponds to swarming motion, active turbulence, local alignment, or some other collective state. Without single-particle or local flow-level analysis, these distinctions remain unclear. I encourage the authors to look more carefully into the bacterial collective dynamics, especially at 35%, before making strong claims.
5. Another important point concerns how the bacterial volume fraction inside droplets was measured. Since single-bacterium visualization and counting are not clearly shown in the manuscript, it is difficult to assess how accurately the reported volume fractions were determined. In a system like this, I would expect substantial droplet-to-droplet variation, both in droplet size and in the number of encapsulated bacteria. This becomes especially important because the main conclusions rely on comparing only two bacterial volume fractions. Were the reported values obtained from direct counting, fluorescence intensity calibration, or some indirect estimate? How many droplets were analyzed to determine these values, and what is the spread in bacterial number for droplets of comparable size? Without this information, it is difficult to know how well controlled the bacterial loading actually is, and whether the observed differences reflect systematic changes in concentration or simply broad heterogeneity across droplets

6. The way the interfacial tension is measured is not described with sufficient clarity. How exactly was the contour extracted? How many droplets were analyzed for each condition? Were the spectra obtained from single representative droplets or from an ensemble average? This is important because the main conclusions rely strongly on the fluctuation spectra.
7. I am not fully convinced by the statement that the scale-dependent spectra demonstrate a fundamentally nonequilibrium redistribution of interfacial energy, in contrast to equilibrium-like equipartition. The large-k scaling appears more like an equilibrium, Helfrich-like decay. If bacterial activity indeed enhances interfacial fluctuations, I would instead expect the stronger effect to appear at low wave numbers or low mode numbers, similar to what has been reported for bacteria encapsulated inside GUVs (see refs. 19 and 20). This point should be discussed much more carefully.
8. The continuum model may reproduce some of the phenomenology, but the connection to the experiment remains somewhat indirect. Important ingredients such as bacterial tumbling, discrete steric effects, near-wall hydrodynamics, and local ordering are not explicitly resolved. I think the authors should be more careful not to overinterpret the model as establishing the mechanism.
9. The most striking observations in the paper, namely the strong deformations and tether-like protrusions at high bacterial concentration, are also the least mechanistically explained. What sets the width, lifetime, and frequency of these protrusions? Are they true stable structures, or transient interfacial failures? Since the bacteria appear aligned along the tube axis, does local curvature promote accumulation and alignment, or do aligned bacteria generate the protrusions? This feedback is interesting, but not sufficiently discussed.
10. Since much of the later dynamics is observed after sedimentation to the substrate, wall effects likely play an important role. The enhanced near-wall motility and faster coarsening may not only reflect active droplet physics, but also wall-induced effects and asymmetric confinement. This limitation should be discussed much more explicitly.
11. Several important experimental details are missing or too brief. These include: the number of droplets analyzed, the

number of independent experiments, uncertainty in bacterial volume fraction, temporal stability of motility, and whether oxygen depletion or cell viability were controlled. These controls are important, especially because the conclusions mainly rely on comparing only two concentrations.
12. More broadly, the manuscript would benefit from a more careful discussion of previous work on active interfaces, active vesicles, and bacteria-driven deformations of soft boundaries. The distinction between a liquid–liquid interface and a membrane is important, but there are still conceptual overlaps that should be discussed more critically.
Minor points:
1. Error bars and droplet-to-droplet variability are largely missing in the main text. Are the reported spectra based on single droplets, averaged over several droplets, or averaged over repeated experiments?
2. The discussion of the high-concentration regime would benefit from more quantitative analysis of local bacterial alignment inside the protrusions.

(Remarks on code availability)
The manuscript also lacks a clear data and code availability statement.

Reviewer #3

(Remarks to the Author)
Summary:
The manuscript presents experimental data and simulations that show waves and large deformations on the surfaces of fluid droplets due to bacterial motility induced active stresses. The work is well-executed, and the manuscript is overall interesting to read. However, there are major points outlined below that need to be addressed before the work can be published.
Major Comments:
1. As the manuscripts stands right now, most of the phenomena (effective non-inertial propagating waves and large dynamic protrusions) observed by them and even the analysis methodology can be found in several publications, many of which are cited in the manuscript. Therefore, it is crucial that authors state exactly what is the research gap that their study fills in and why that is non-trivial/important. For example, it is not enough to say that few experiments have realized closed liquid-liquid droplets that are actively deformed from within and that the presented experiment adds to this list. Title and abstract should reflect this level of detail.
2. The discussion section states “Whereas interfacial waves in active nematics are typically attributed to the nonreciprocal coupling between the nematic director and the interface [16, 58], our results reveal a hydrodynamic mechanism based on viscosity matching between active and passive fluids.” I agree with the authors that their theory based on viscosity mismatch explains the presented data well. But the negative viscosity of the active fluid in their model arises primarily due to the formation of an active nematic phase. So, how are the authors so sure that a mechanism based on non-reciprocal coupling between the nematic director and the interface will not be good enough to explain the data. In other words, multiple models can explain the same set of data in which case it becomes important to address the physical considerations to rule out some of them.
3. Fig. 2c,g show that S(k) scales as k^{-2} for $k<k_c$ and k^{-4} for $k>k_c$. Usually, one associates k^{-2} scaling as equilibrium capillary waves (Science, 304, 847 (2004)). So it is non-intuitive to associate the whole spectrum regardless of scaling laws as associated with waves generated by active stresses. Other works on droplets with active particles inside them have only seen k^{-4} scaling (Communications Physics, 5, 91 (2022)). Can the authors comment on this?
4. The authors mention: “As k increases, $S_n(k, \omega)$ no longer displays distinct wave peaks. This transition occurs at a k_c , marked by the dashed line in Fig. 2d, which approximately corresponds to the scale of the velocity correlation length ℓ_v.” This suggests that when the correlation length is lower (higher k), these wave peaks would extend even more (FIG. S18). But a lower correlation length implies weaker collective behaviour. Since the authors claim that the scale-dependent fluctuations arise from collective bacterial flows, this seems like a contradiction. Is collective behaviour the sole cause of the propagating waves?
a) If there were no collective behaviour but random active stresses, will there be any enhanced fluctuations or propagating waves?
b) Can the authors clarify what would happen if the correlation length were very low (lower than shown in FIG S18)?
c) In FIG. S18, it would be appropriate to include snapshots of the flow fields.
5 The authors have mentioned that they used the pre-separated PEG–dextran phases (PEG rich and dextran rich phases) and then mixed them with the bacterial suspensions. Can they shed some light on why this is being done, instead of directly mixing PEG, dextran and bacterial cells?
Minor Comments:
1. The labels in the insets of the figures 2 (g) and 3 (c) are too faint to read.
2. Figure 2 caption for panel (h) states R=32. Are the units missing or was this some dimensionless radius being specified?. Either way, this should be stated in the caption.

(Remarks on code availability)

Reviewer #4

(Remarks to the Author)
I co-reviewed this manuscript with one of the reviewers who provided the listed reports. This is part of the Nature Communications initiative to facilitate training in peer review and to provide appropriate recognition for Early Career

Researchers who co-review manuscripts.

(Remarks on code availability)

Version 1:

Reviewer comments:

Reviewer #1

(Remarks to the Author)
I thank the authors for their thorough and thoughtful responses to all of my previous comments. The revised manuscript has been significantly strengthened through the addition of new control experiments, clarification of the simulation framework and parameter choices, and expansion of the discussion regarding the physical mechanisms underlying the observed phenomena.

In particular, the non-motile bacterial control experiments provide important evidence distinguishing passive sedimentation effects from activity-driven dynamics. The authors have also carefully clarified the interpretation of the effective inertial response, the role of active viscosity, and the distinction between the low-activity wave regime and the high-activity deformation regime. Furthermore, the comparison with previously reported active-interface systems helps clearly establish the novelty and significance of the present work.

Overall, I find that the authors have satisfactorily addressed all of my concerns. The manuscript now presents a compelling combination of experiments, quantitative analysis, and theoretical modeling that advances our understanding of active liquid-liquid phase-separated systems. I believe this work will be of broad interest to the active matter and soft condensed matter communities.

I have no further comments and support the publication of the manuscript with this current version.

(Remarks on code availability)

Reviewer #2

(Remarks to the Author)
I am satisfied with the authors' replies to my questions. I think the manuscript is ready for publication.

(Remarks on code availability)

Reviewer #3

(Remarks to the Author)
The authors have addressed my concerns satisfactorily. I recommend publication of the manuscript.

(Remarks on code availability)

Reviewer #4

(Remarks to the Author)
I co-reviewed this manuscript with one of the reviewers who provided the listed reports. This is part of the Nature Communications initiative to facilitate training in peer review and to provide appropriate recognition for Early Career Researchers who co-review manuscripts.

(Remarks on code availability)

Open Access This Peer Review File is licensed under a Creative Commons Attribution 4.0 International License, which permits use, sharing, adaptation, distribution and reproduction in any medium or format, as long as you give appropriate credit to the original author(s) and the source, provide a link to the Creative Commons license, and indicate if changes were made.
In cases where reviewers are anonymous, credit should be given to 'Anonymous Referee' and the source.
The images or other third party material in this Peer Review File are included in the article's Creative Commons license, unless indicated otherwise in a credit line to the material. If material is not included in the article's Creative Commons license and your intended use is not permitted by statutory regulation or exceeds the permitted use, you will need to obtain permission directly from the copyright holder.
To view a copy of this license, visit https://creativecommons.org/licenses/by/4.0/

<u>Reply to Reviewer #1</u>:

This manuscript presents an original, visually compelling, and mechanistically insightful experimental-theoretical study of internally active liquid-liquid phase-separated (LLPS) droplets powered by motile bacteria. The platform is clever and minimal, bridging synthetic biology, active matter hydrodynamics, and non-equilibrium multiphase systems.

They found that in low-activity droplets, propagating interfacial waves via viscosity matching in low-Re regime, high-activity droplets sustained super-Plateau deformations with cell-sized filaments, enhanced motility, and dramatically accelerated coarsening and sedimentation.

These results are novel and well-supported by confocal imaging, PIV, structure factor analysis, TTSH + Cahn-Hilliard simulations. The "effective inertial response," arising from active stresses that counteract viscous damping, represents a novel hydrodynamic mechanism for generating underdamped dynamics in confined active multiphase fluids, distinct from director-mediated coupling in active nematics or localized forcing mechanisms in active vesicles reported in previous studies.

This work demonstrates clear significance for understanding activity-driven reprogramming of phase-separated systems (relevant to biomolecular condensates under cellular forces or programmable synthetic compartments). Overall, this is a strong and original contribution that is suitable for publication after minor revisions. The main text is generally clear and logically structured, but several points require clarification showed below.

<u>**Comment 1.1**</u> *Bacterial cells have a density slightly higher than water, With increasing bacterial volume fraction, whether the dextran-rich droplet phase becomes denser than the surrounding PEG-rich phase? In Fig. 1, the accelerated sedimentation (<10 min to chamber bottom) and coarsening are attributed primarily to activity-induced motility, flows, and enhanced coalescence. However, could buoyancy/density mismatch contribute significantly on the 10–30 min timescale? A control experiments that with non-motile or dead bacteria at the same φ_b would help disentangle these contributions*

Response: We thank the Reviewer for this important suggestion. To distinguish this passive effect from activity, we performed control experiments with non-motile bacteria (RP1616) at comparable volume fractions. The coarsening rate has the following order: no bacteria < non-motile bacteria < motile bacteria. In both the non-motile and motile groups, higher bacterial loading leads to faster coarsening. Thus, non-motile bacteria do accelerate coarsening relative to bacteria-free droplets, confirming a passive contribution from bacterial loading and density mismatch. However, motile bacteria produce faster coarsening than non-motile controls at comparable loading, indicating an additional activity-dependent contribution from motility-induced flows, droplet deformation, and enhanced droplet encounters.

Revision: To address this concern, we added a control experiment and the corresponding discussion in the Supplementary Information (Fig. S14-S16). Specifically, we used the non-motile bacterial strain RP1616 at the same volume fraction as a control. We now state in the SI (Sec. S3D) that, when viewed as a passive suspension, bacteria can also enhance sedimentation. However, motile bacteria induce even faster sedimentation and coarsening, particularly in groups with high bacterial volume fraction.

<u>**Comment 1.2**</u> *In Figure 2e–h (and related simulation results), droplet radii are given as R=12 and R=32 with no units, the same for velocity. Could the authors clarify the unit system for the simulations (and any conversion to physical scales) to ensure readers can directly compare with experiments?*

Response: We thank the Reviewer for pointing this out. We have clarified that the quantities shown in Fig. 2(e--h) are reported in nondimensional simulation units. Following the approach used by Dunkel et al. [PRL 110, 228102 (2013)], we relate these units to experimental scales by matching the velocity-correlation length and the characteristic collective-flow speed.

In our simulations, the length unit is set by the default active-flow length scale $\sqrt{\zeta/|\nu_2|} = 1$ where $\nu_2<0$ is the negative viscosity that drives the instability of the bacterial flow, and $\zeta > 0$ is the higher-order viscosity that selects a correlation length scale. With this default choice, the velocity correlation length measured from the simulated active flow is $\ell_v \simeq 5$ as shown in Fig. S5.

For the smaller droplet shown in Fig. 2a, with $R = 39.6$ μm, the measured correlation length is approximately 20μm (See Fig. S17), therefore the scale bar (10 simulation length units) in Fig. 2e corresponds to 40 μm. For the larger droplet shown in Fig. 2b, with $R = 273$ μm, the measured correlation length is approximately 50μm (See Fig. S17), therefore the scale bar (10 simulation length units) in Fig. 2f corresponds to 100 μm.

The velocity unit is calibrated in the same way by comparing the characteristic simulation speed v_0 with the experimentally measured bacterial-flow speed. In the simulations, the mean active-flow speed is $v_0 = 0.1$ in simulation units. For the droplet in Fig. 2a and b, the corresponding experimental mean flow speed is approximately 3 μm/s, so the velocity scale of 0.2 simulation units in the colorbar corresponds to 6 μm/s.

Revision: We have revised the caption of Fig. 2 to explicitly state that the simulation radii and velocities are given in nondimensional simulation units.

We have also revised the Supplementary Information to clarify the unit mapping used for the simulations. In the SI Sec. S2, we state explicitly that simulation units are related

to experiments by matching the characteristic velocity-correlation length and collective-flow speed.

Comment 1.3 *Figure 3 caption refers to "simulations of a spherical (top) and flat (bottom) interface." Does the flat interface case correspond to the results shown in Fig. S14d–e?*

Response and Revision: Yes. The flat-interface case shown in the bottom panels of Fig. 3 corresponds to the same 2D flat-interface simulations presented in Fig. S20d, e (S14d, e in the previous version).
We have clarified this connection in the Fig. 3 caption: "Details of the initial-perturbation analysis are provided in Fig. S20d-f."
We have clarified this connection in the Fig. S20 caption: "For (d-f), the flat-interface setup as the same as that shown in Fig. 3d in the main text."

Comment 1.4 *the authors set $\nu_1 + \nu_2 \approx 0$ (viscosity matching) to achieve near-zero effective damping and underdamped waves. How is the magnitude of the negative active viscosity ν_2 specifically chosen? Is it purely phenomenological/tuned to match observed wave cutoff and frequencies, or is there literature/experimental support for its order of magnitude in bacterial suspensions? Prior works on bacterial "superfluids" report negative effective viscosity primarily at very low shear rates (near-zero or weak flows, e.g., in confined Couette or microfluidic rheometry), what is the typical shear rate in the droplet?*

Response: We thank the Reviewer for this question. We use a set of dimensionless parameters similar to those used by Dunkel et al. [Dunkel et al., Phys. Rev. Lett. 110, 228102 (2013)], as listed in Table S1. These parameters are first chosen to describe the bulk collective bacterial flow, following the same nondimensionalization strategy in which the simulation units are related to experiments by matching the characteristic velocity-correlation length and the collective-flow speed. After coupling the active bacterial phase to the liquid-liquid interface, we keep the same parameters fixed. Therefore, ν_2 is constrained by the bulk properties of bacterial turbulence, rather than being tuned solely to reproduce the observed interfacial wave dispersion.

The coefficient ν_2 should be understood as an effective coarse-grained active coefficient in the TTSH description. It is related to the flow-amplifying effect of collective pusher-type bacterial motion, but it is not intended to be compared one-to-one with a rheological viscosity measured under steady imposed shear. We therefore use the bacterial-rheology literature as physical motivation, not as a direct quantitative input for choosing ν_2.

We have added an estimate of the typical shear rate inside the droplets. From the measured internal flow speed and vortex size, $\dot{\gamma}_{\mathrm{drop}} \sim U/\ell_v$. For U ~ 3-4μm/s and

$\ell_v \simeq 20$–$50\mu m$, this gives a typical shear rate $\dot{\gamma}_{\text{drop}} \sim 0.06$–$0.2\ s^{-1}$. If the intermittent maximum speed $U \sim 10\mu m/s$ is used, local values can reach approximately $\dot{\gamma}_{\text{drop}} \sim 0.5\ s^{-1}$. This is a low-shear regime, although the comparison with Couette rheometry should be made cautiously because the droplet flow is unsteady, vortical, and spatially heterogeneous rather than a steady imposed shear.

Revision: We revised SI Sec. S2 to clarify the selection of ν_2 in the simulations.

Comment 1.5 *In Figure 4, aspect ratios exceed the classical Rayleigh-Plateau threshold π and are sustained for seconds, with excess surface area $\Delta \approx 0.6$ and formation of cell-sized filaments (bacteria nematically aligned along the axis). What is the dominant physical mechanism stabilizing these extreme, non-equilibrium shapes against capillary breakup?*

Response: For the globally elongated droplets, the dominant mechanism responsible for their several-second persistence has not yet been fully resolved. We therefore avoid making a strong mechanistic claim in the manuscript. Our current interpretation is that two plausible, but still unconfirmed, effects may contribute to this effect. **First**, active forcing generated by the bacterial turbulent flow acts from inside the droplet and can produce unsteady pressure fields and recirculating flows. Because the droplet volume is conserved and the flow is incompressible, local interfacial deformations are coupled to the global pressure and velocity field. This coupling may allow internally generated active fluctuations to partially counteract the capillary pressure gradients that would otherwise promote necking and breakup. **Second**, active flows near the interface may generate tangential interfacial motion. In a spatially modulated protrusion, such tangential motion can redistribute fluid and momentum along the interface and may smooth axial variations between necks and bulges. This effect is conceptually similar to a dynamic self-healing process, but it remains a possible physical scenario rather than a demonstrated mechanism.

The cell-sized filaments represent a different deformation regime. Their formation reflects the particulate nature of the bacterial suspension: when the protrusion width becomes comparable to the bacterial length scale, local confinement can organize bacteria along the filament axis (See Fig. S36). Thus, the observed nematic alignment should be understood as local organization within a confined, transient protrusive structure. These filaments are not stable structures; they are transient and often retract or break (See Fig. S35 and Movie S8). Therefore, we do not interpret cell-sized filaments as being stabilized against Rayleigh-Plateau breakup.

Revision: We have revised the main text to use more cautious language and to avoid implying that the suppression of breakup is already fully understood.

The revised text in paragraph 1 on page 5 now states: "These observations suggest that internal active forcing can transiently maintain highly elongated interfacial configurations beyond the passive capillary-breakup expectation."

The revised text in paragraph 2 on page 7 now states: "These observations suggest that internal activity may help maintain strongly deformed, connected interfacial configurations that have no direct passive counterpart."

In addition, we have expanded the Supplementary Information to clarify this point. In Sec. S5A, we added a discussion of the possible mechanism by which active internal flows may suppress Plateau-Rayleigh-type breakup. We also added Sec. S5C to distinguish two types of protrusions observed in the experiments.

Comment 1.6 *Do the low-activity "effective inertial response" (propagating waves via viscosity matching) and high-activity "sustained super-Plateau deformations + filaments" share the same mechanism?*

Response: We thank the Reviewer for this important question. Our view is that the two phenomena share the same broad nonequilibrium origin, namely the coupling between internally generated bacterial activity and a deformable liquid–liquid interface, but they represent different dynamical regimes rather than one identical mechanism.

At low activity, droplets remain close to spherical, and the interface dynamics are small-amplitude and near-linear. Activity mainly renormalizes the hydrodynamic damping: the negative effective viscosity of the active phase partially compensates the viscous damping from the passive exterior, making the interface effectively underdamped and allowing propagating capillary-like modes.

At high activity, the dynamics become strongly nonlinear. Active stresses directly drive large protrusions and thin filaments, while flow–shape feedback can transiently sustain elongated structures beyond the passive Rayleigh–Plateau expectation. Thus, the low-activity waves reflect activity-reduced damping, whereas the high-activity deformations reflect nonlinear active forcing and flow–shape feedback.

Revision: We have added a comment in the main text in the first paragraph on page 6: "Together, the interfacial wave modes, protrusions, and bacteria-scale filaments show that bacterial droplets do not follow a single interfacial response mechanism. Instead, they exhibit activity-dependent regimes ranging from near-linear propagating waves to nonlinear large-scale deformation and localized bacterial alignment."

We also added a new subsection in Sec. S5C to compare the mechanism of global deformation and thin filaments.

Comment 1.7 *In the Discussion, to better highlight novelty, could the authors more explicitly contrast this with wave propagation mechanisms in other active interface*

literature, such as nonreciprocal director-velocity coupling in active nematics, or contractility-driven modes in active vesicles?

Response: We thank the Reviewer for this helpful suggestion. In active nematic liquid–liquid interfaces, propagating modes are commonly understood as arising from coupling between interfacial shape, flow, and the nematic director field. In particular, recent theories emphasize nonreciprocal coupling between the interface and orientational degrees of freedom: director distortions drive interfacial motion, while interfacial deformation and flow feedback on the director dynamics. In this mechanism, the nematic director is an explicit and essential dynamical variable.

Active vesicles provide another distinct route to propagating or oscillatory shape dynamics. In that case, the boundary is an elastic membrane. Contractile stresses, membrane bending elasticity, membrane tension, and area constraints can together generate active shape modes. Thus, the relevant restoring forces and dissipation channels are membrane-based.

Our system differs from both cases. The interface is a liquid–liquid boundary with surface tension but no membrane bending elasticity, and the low-amplitude wave dynamics can be described without introducing an explicit interfacial director field. Instead, our theory and simulations identify a hydrodynamic damping-compensation mechanism. Collective bacterial motion modifies the effective viscous response of the droplet phase, so that active stresses partially compensate the viscous damping from the passive outer phase. When this compensation is sufficient, selected long-wavelength interfacial modes become effectively underdamped even in the low-Reynolds-number regime.

This comparison clarifies the novelty of our interpretation. We do not simply claim that active interfaces can support waves; rather, we identify a bulk active-rheological route to wave propagation in a closed bacterial liquid–liquid droplet. This mechanism is distinct from nonreciprocal director–interface coupling in active nematics and from contractility- or membrane-elasticity-based shape modes in active vesicles.

Revision: We revised the Discussion in the last paragraph on page 6: "Interfacial waves in active nematics are typically attributed to nonreciprocal coupling between the nematic director and the interface. Traveling membrane deformations in active vesicles, on the other hand, occur on elastic boundaries, where membrane bending contributes to the restoring force. In contrast, our results suggest a continuum-level hydrodynamic mechanism in which activity-induced viscosity reduction in the bacterial phase compensates viscous damping from the passive phase."

We also added a Supplementary Information subsection Sec. S4G, "Comparison with other active-interface wave mechanisms".

Reply to Reviewer #2:

Chang et al. investigate phase-separated PEG–dextran droplets that encapsulate motile E. coli and ask how active stresses generated by E. coli affect the dynamics and shape fluctuations of a closed liquid–liquid interface. By varying the bacterial volume fraction inside the dextran-rich phase, the authors report two distinct regimes. At lower bacterial volume fraction, droplets remain nearly spherical but display strong interfacial fluctuations, including a crossover in the static fluctuation spectrum from $S(k) \sim k^{-2}$ *at small k to* $S(k) \sim k^{-4}$ *at larger k, together with finite-frequency peaks in the dynamic spectrum that are interpreted as wave-like interfacial modes. At higher bacterial volume fraction, droplets show much stronger deformations, tether-like protrusions, enhanced near-wall motility after sedimentation, and faster coarsening. The authors support these observations using a Cahn–Hilliard + Toner–Tu–Swift–Hohenberg continuum model and an analytical flat-interface treatment. Overall, the topic is timely and relevant for the active soft matter community. However, I am not convinced by the present interpretation, and I think the manuscript requires major revision before it can be considered further.*

Here are some specific comments, which might be helpful for future submissions:

Comment 2.1 *The central claim that increased bacterial concentration corresponds to increased activity is not convincing. Increasing concentration is not equivalent to increasing activity at the level of individual bacteria. Activity should be defined through quantities such as swimming speed, persistence, or a suitable dimensionless activity parameter like Pe number. In contrast, increasing concentration mainly changes collision frequency, crowding, hydrodynamic coupling, and the onset of collective motion. The manuscript currently conflates these effects*

Response: We thank the reviewer for this important comment. There are two pieces of experimental evidence indicating that the high-bacterial-density droplets correspond to a higher activity.

First, the droplet morphology becomes much more strongly deformed at higher bacterial loading. In the lower-concentration condition, droplets remain close to spherical and exhibit relatively weak interfacial deformation, as shown in Fig. 2a, b. In contrast, at the higher bacterial volume fraction, droplets display pronounced elongation, twisting, and protrusion formation, as shown in Figs. 4a and S29, S31, and S33. Since these deformations are driven by active stresses exerted by the bacterial suspension on the liquid-liquid interface, the stronger deformation provides direct evidence that the coarse-grained active forcing is enhanced in the high-loading regime.

Second, the measured internal bacterial flow field is also stronger at higher bacterial loading. In Fig. S4, we show the velocity distributions for the two bacterial-volume-fraction conditions. The mean internal flow speed increases from approximately $3 \mu\text{m/s}$ in the lower-concentration droplets to approximately $4 \, \mu\text{m/s}$ in the higher-concentration droplets. In addition, the higher-concentration droplets exhibit a stronger

high-speed tail, with maximum speeds reaching approximately 15–20 μm/s. Thus, although bacterial concentration should not be equated with single-cell activity, the measured collective flow field is stronger at higher bacterial loading.

One possible way to understand the link between bacterial loading and effective activity is through the coarse-grained active stress generated by the suspension, rather than through the motility of individual bacteria. In a coarse-grained description, this can be expressed schematically as $\sigma_{ij}^{a} \sim -n_b S Q_{ij}$, where n_b is the bacterial number density, S is the stresslet strength of an individual swimmer, and Q_{ij} represents the coarse-grained local orientational order tensor. Thus, even if the single-bacterium swimming speed does not significantly increase, the collective active stress exerted on the liquid–liquid interface can increase with bacterial loading.

Revision: We have revised the terminology throughout the manuscript to avoid using "activity" interchangeably with bacterial concentration in the experimental description. Specifically, we revised main text and Supplementary Information to describe the experimental conditions primarily in terms of bacterial volume fraction or bacterial density. The term "activity" is now retained only in the context of the coarse-grained TTSH model, where it refers to the effective active forcing or active stress generated by the bacterial suspension.

We have added an explanation in paragraph 3 on page 2 to clarify the relationship between bacterial volume fraction and activity: "Under both volume fractions, the encapsulated bacteria exhibit turbulence-like collective motion. Increasing φ_b increases the collective active forcing generated per unit droplet volume and therefore corresponds to a higher collective activity of the bacterial suspension. As we show below, these two loading conditions lead to qualitatively distinct regimes of interfacial dynamics."

We also added Fig. S4 to show the velocity distributions at different bacterial concentrations. In Sec. S1F3, we added a statement that: "Operationally, the low- and high-activity cases in the model correspond to the low- and high-bacterial-volume-fraction droplets in the experiments, respectively."

Comment 2.2 *Related to this, without resolving the bacterial dynamics more carefully, it is difficult to interpret the physical state of the system at different concentrations. At high bacterial volume fraction, are the bacteria still individually motile, or is the system dominated by collective motion, crowding, or even partial kinetic arrest? The manuscript uses terms such as collective flow and active turbulence, but these claims are not sufficiently supported by direct characterization of bacterial motion.*
Comment 2.4 *It is also not clear whether the high-concentration state corresponds to swarming motion, active turbulence, local alignment, or some other collective state.*

Without single-particle or local flow-level analysis, these distinctions remain unclear. I encourage the authors to look more carefully into the bacterial collective dynamics, especially at 35%, before making strong claims.

Response: We thank the reviewer for this important comment. The bacterial state inside the droplets is best described as a **bacterial-turbulent state**.

First, direct visualization supports the presence of bacterial-turbulent dynamics inside the droplets. As shown in Movie S2, bacteria in the low-bacterial-volume-fraction droplets exhibit collective chaotic motion rather than independent swimming. In the high-bacterial-volume-fraction droplets, Movies S6-S9 show persistent shape deformation, indicating that the system remains dynamically active and is not kinetically arrested.

We then quantify the resulting coarse-grained flow field using the PIV method. As shown in Figs. 2a, b and 4a, PIV measurements reveal persistent chaotic internal flows, vortical and jet-like structures, and long-ranged velocity correlations in both bacterial-volume-fraction conditions.

This interpretation is further supported by quantitative measurements of the internal flow field. As shown in Fig. S17a, the velocity-correlation length is approximately 20-50 μm, much larger than the size of a single bacterium. This separation of length scales indicates that the observed motion is collective rather than dominated by independent swimming. Moreover, as shown in Fig. S17b, ℓ_v increases with droplet size following a power-law trend, consistent with recent evidence that collective motion in bacterial suspensions can be scale-free [Pérez-Estay et al., PNAS 123, e2600266123 (2026)].The velocity power spectra shown in Fig. S17c,d further exhibit scaling behaviors consistent with those reported in previous studies of bacterial turbulence under spherical confinement [Granick et al., Soft Matter 18, 6419-6425 (2022)] and near rigid walls [Peng et al., Adv. Sci. 11, 2402643 (2024)].

At the same time, the droplet interior should not be interpreted as a globally ordered state. We do not observe a system-spanning coherent circulation in which the bacterial population rotates collectively around the droplet, as reported in confined bacterial suspensions [Wioland et al., Phys. Rev. Lett. 110, 268102 (2013)]. Nor do we observe persistent long-range nematic order throughout the droplet interior. In representative high-resolution images used for bacterial-volume-fraction estimation (Fig. S2), bacteria do not show persistent interfacial nematic alignment.

This observation of bacterial turbulence is also consistent with previous studies of dense bacterial suspensions. In our experiments, the bacterial volume fractions $\phi_b \simeq 21\%$ and 34%, far above the reported onset concentration for bacterial turbulence in three-dimensional E. coli suspensions, $\phi_c \simeq 1.8\%$ [Peng et al., Sci. Adv. 7, eabd1240 (2021)]. Sokolov et al. reported collective bacterial dynamics over a broad

concentration range, $\phi_b \simeq 10\% - 90\%$ [Sokolov et al., Phys. Rev. Lett. 98, 158102 (2007)], which covers the concentration range used here. Besides, the three-dimensional bacterial-turbulence experiments analyzed by Dunkel et al. were performed at dense bacterial volume fractions of approximately 30%-40% [Dunkel et al., Phys. Rev. Lett. 110, 228102 (2013)], comparable to our high-volume-fraction condition. In addition, our bacterial volume fractions are much below the reported glass transition threshold of bacterial suspension, $\phi_b \simeq$ 85%-88% [Lama et al., PNAS Nexus 3, pgae238 (2024)].

Taken together, the direct observation of motile bacteria, PIV-resolved chaotic collective flows, long velocity-correlation length, velocity-spectrum scaling, absence of global nematic order, absence of kinetic arrest, and consistency with previously reported concentration ranges all support the interpretation that the droplet interior is in a bacterial-turbulent state.

Revision: We revised the title to ``Bacterial turbulence drives interfacial waves and shape dynamics in phase-separated droplets" to more clearly indicate the collective-flow state inside the droplets.

We revised the Introduction to state that this system provides a minimal setting in which a closed liquid-liquid interface is driven internally by **bacterial turbulence**.

We added an explanation in paragraph 3 on page 2 to clarify the bacterial turbulence state: "Under both volume fractions, the encapsulated bacteria exhibit turbulence-like collective motion."

We added Fig. S2 to show that bacteria do not reveal long-range nematic order in the droplet interior.

We also revised SI Sec. S1F to emphasize that our system is not kinetically arrested.

Comment 2.3 *I could not find a clear quantification of the bacterial swimming speed, even in the dilute limit. This is an important missing control. If the authors want to compare two concentrations and interpret them in terms of activity, they should at least report the individual bacterial speed distributions and how these change with concentration and time.*

Response and Revision: To characterize the bacterial dynamics more directly, we added a new SI subsection (Sec. S1F3), "Flow fields of bacterial droplets at two concentrations", together with Fig. S4, to show the velocity distributions of individual bacteria and collective bacterial flows at two bacterial volume fractions.

We first measured the swimming-speed distribution of individual bacteria in the dilute limit. The mean single-cell swimming speed is approximately $2.5\mu\mathrm{m/s}$. This relatively

low value is expected because the bacteria swim in a polymer-rich aqueous two-phase medium, whose viscosity is higher than that of standard motility buffer.

For the collective motion inside bacterial droplets, the mean internal flow speed is approximately $3\ \mu m/s$ in the lower-bacterial-volume-fraction droplets and increases to approximately $4\ \mu m/s$ in the higher-bacterial-volume-fraction droplets. In addition, the maximum flow speed in the higher-concentration case reaches approximately 15–20 μm/s, corresponding to a stronger high-speed tail in the velocity distribution. These stronger flows are associated with the jet-like events and pronounced protrusions observed in the high-concentration droplets.

Comment 2.5 *Another important point concerns how the bacterial volume fraction inside droplets was measured. Since single-bacterium visualization and counting are not clearly shown in the manuscript, it is difficult to assess how accurately the reported volume fractions were determined. In a system like this, I would expect substantial droplet-to-droplet variation, both in droplet size and in the number of encapsulated bacteria. This becomes especially important because the main conclusions rely on comparing only two bacterial volume fractions. Were the reported values obtained from direct counting, fluorescence intensity calibration, or some indirect estimate? How many droplets were analyzed to determine these values, and what is the spread in bacterial number for droplets of comparable size? Without this information, it is difficult to know how well controlled the bacterial loading actually is, and whether the observed differences reflect systematic changes in concentration or simply broad heterogeneity across droplets*

Response: To quantify the bacterial density, we used 3D confocal microscopy in a super-resolution optical reassignment imaging mode that improves the spatial resolution for resolving individual bacteria. We manually identified the bacteria and then calculated the volume fraction. A representative example is now shown in Fig. S2 of the Supplementary Information.

We analyzed five droplets for the low-bacterial-concentration condition and five droplets for the high-bacterial-concentration condition. From these measurements, we obtained a bacterial volume fraction of $21\% \pm 4\%$ for the low-concentration state and $34\% \pm 3\%$ for the high-concentration state, where the uncertainty represents the droplet-to-droplet variation across the measured samples.

We agree that some heterogeneity from droplet to droplet is unavoidable in this system, due to variations in both droplet size and bacterial encapsulation. However, the measured distributions for the two conditions remain clearly separated within the reported spread, indicating that the observed differences are associated with a systematic change in bacterial loading rather than solely with uncontrolled droplet-to-droplet heterogeneity.

Revision: We revised the main text to report the bacterial volume fractions quantitatively. Specifically, we now state that the measured bacterial volume fraction is $21\% \pm 4\%$ for the low-concentration state and $34\% \pm 3\%$ for the high-concentration state, where the uncertainty reflects droplet-to-droplet variation. We also added a new subsection in the Supplementary Information, SI Sec. S1F1, "Estimation of bacterial volume fraction," to describe how the bacterial concentrations were measured experimentally.

Comment 2.6 *The way the interfacial tension is measured is not described with sufficient clarity. How exactly was the contour extracted? How many droplets were analyzed for each condition? Were the spectra obtained from single representative droplets or from an ensemble average? This is important because the main conclusions rely strongly on the fluctuation spectra.*

Response: We determine the interfacial tension of large droplets by fitting their side-view profiles, as shown in the Supplementary Information, where we now include representative images, the measurement procedure, and the corresponding fitting quality (see Fig. S1 in the Supplementary Information). We measured 8 independent droplets and obtained an interfacial tension of 7.5 ± 1.7 μN/m.

The fluctuation spectra are measured for individual droplets, rather than ensemble-averaged over different droplets, because the spectra depend on droplet radius and therefore vary from droplet to droplet. Accordingly, our reported spectra are obtained by time averaging for each single droplet. In the experiments, we measured about 30 droplets with different radii and selected four representative droplets of different sizes for display in the main text. In Fig. 2c, each curve corresponds to data from one individual droplet. For each droplet, one long experimental acquisition was divided into 30 statistically independent segments. The fluctuation spectrum was first calculated separately for each segment, and the mean spectrum was then obtained by averaging over these 30 segment-resolved spectra. The standard deviation among the 30 spectra was used to estimate the segment-to-segment fluctuation at each wave number.

Revise: In the main text, we now report the measured interfacial tension as 7.5 ± 1.7 μN/m. In SI Sec. S1C, we provide a more detailed description of the surface-tension measurement, including the side-view imaging, contour extraction, and profile-fitting procedure. In SI Sec. S3A, we clarify how the fluctuation spectra were calculated and state the number of droplets analyzed. We also explain that each spectrum shown in the main text corresponds to an individual droplet, with temporal averaging performed over independent segments of the same droplet trajectory.

Comment 2.7 *I am not fully convinced by the statement that the scale-dependent spectra demonstrate a fundamentally nonequilibrium redistribution of interfacial energy, in contrast to equilibrium-like equipartition. The large-k scaling appears more like an equilibrium, Helfrich-like decay. If bacterial activity indeed enhances interfacial*

fluctuations, I would instead expect the stronger effect to appear at low wave numbers or low mode numbers, similar to what has been reported for bacteria encapsulated inside GUVs (see refs. 19 and 20). This point should be discussed much more carefully.

Response: We thank the Reviewer for this important comment. In equilibrium membrane systems such as thermally fluctuating GUVs, a crossover from a tension-dominated k^{-2} regime at low wavenumber to a bending-dominated k^{-4} regime at higher wavenumber can occur while equipartition still holds. Thus, the existence of a spectral crossover alone should not be taken as sufficient evidence for nonequilibrium activity.

Our system differs from such membrane systems in two important ways. First, the boundary in our experiments is a liquid–liquid interface, not an elastic membrane. **It has interfacial tension but no membrane bending rigidity.** Therefore, an equilibrium Helfrich-like bending spectrum is not expected. For a passive liquid–liquid interface, the equilibrium capillary spectrum would be primarily tension-controlled, $S(k) \sim k^{-2}$, apart from gravity, finite-size effects, or experimental resolution limits. Second, **the thermal capillary fluctuations are expected to be very small under our experimental conditions**. Using the measured interfacial tension $\gamma_0 \sim 7.5 \pm 1.7$ μN/m at T≃20◦C, the thermal capillary length scale is $\sqrt{\frac{k_B T}{\gamma_0}} \sim 23$ nm, which is far below the micrometer-scale deformations observed in active droplets and below our experimental resolution. Consistently, bacteria-free droplets of comparable size show negligible interfacial fluctuations. Therefore, the measurable interfacial fluctuations in bacteria-containing droplets are dominated by active forcing, rather than by an equilibrium thermal spectrum that is simply enhanced by activity.

This distinction is important for interpreting the large-k regime. In bacteria-filled GUVs, activity can enhance low mode numbers while higher modes may remain closer to the passive thermal membrane spectrum. In our liquid–liquid droplets, however, the large-k, k^{-4} decay should not be interpreted as a return to an equilibrium Helfrich-like bending regime, because the interface has no bending elasticity and the passive thermal spectrum is not experimentally resolved. Instead, we interpret both scaling regimes as part of an activity-dominated interfacial fluctuation spectrum, with the crossover controlled by the internal active-flow length scale.

Consistent with this interpretation, in both experiments and simulations the crossover occurs near $k\ell_v \sim 1$, where ℓ_v is the measured velocity correlation length of the internal bacterial flow. Modes with wavelengths larger than ℓ_v are driven by coherent collective active flows and show a shallower spectrum, whereas modes below the active-flow scale are increasingly suppressed by capillary relaxation and short-wavelength damping, leading to a steeper decay. The dynamic structure factor further supports this picture, since propagating modes are observed only below a corresponding cutoff. We therefore interpret the crossover as reflecting the scale-dependent coupling

of active bacterial flows to the interface, rather than a passive tension-to-bending crossover.

Revision: In the first paragraph on page 2, we now report the measured interfacial tension, $\gamma_0 \sim 7.5 \pm 1.7$ μN/m, and note that the corresponding thermal capillary amplitude scale, $\sqrt{\frac{k_B T}{\gamma_0}} \sim 23$ nm, is far below the micrometer-scale interfacial deformations resolved in the active droplets.

In paragraph 2 on page 3, we added:" This activity-dominated crossover is distinct from passive equilibrium crossovers: unlike vesicle membranes, the liquid-liquid interface has surface tension but no bending rigidity, and thermal fluctuations are negligible compared with the activity-driven interfacial motion observed here."

In Sec. S3A of the Supplementary Information, we further emphasize this distinction and note that vesicle spectra can already show an equilibrium tension-to-bending crossover while satisfying equipartition. These revisions clarify why the crossover observed in our droplets should not be interpreted as a passive membrane-mechanics crossover, but rather as an activity-dominated crossover associated with the internal collective-flow length scale.

__**Comment 2.8**__ *The continuum model may reproduce some of the phenomenology, but the connection to the experiment remains somewhat indirect. Important ingredients such as bacterial tumbling, discrete steric effects, near-wall hydrodynamics, and local ordering are not explicitly resolved. I think the authors should be more careful not to overinterpret the model as establishing the mechanism.*

Response: We thank the Reviewer for this important comment. In the revised manuscript, we will therefore clarify that the simulations are used as a minimal phenomenological, mesoscale description of the coupling between phase separation, interfacial tension, and internally generated active flows, rather than as a complete microscopic model of the bacterial suspension.

The TTSH model is a well-established coarse-grained framework for dense bacterial suspensions and active turbulence, but it does not explicitly resolve bacterial tumbling, discrete steric interactions, near-wall hydrodynamics, or local orientational ordering. We therefore use the simulations to show that a coarse-grained active-passive hydrodynamic description is sufficient to reproduce several mesoscopic features of the experiments, including collective internal flows, low-activity interfacial waves, and large-scale deformations.

Revision: We have softened the words in paragraph 2 on page 3: To further investigate the spatiotemporal dynamics of the active droplets, we simulate a minimal coarse-grained continuum model of an active–passive fluid mixture that reproduces the key qualitative features of the experiments.

We also softened the words in section “Numerical simulation” in Method and claim: In this minimal model, the droplet is treated as a closed liquid-liquid interface, while substrate-induced hydrodynamic and contact-line effects are not explicitly included.

Comment 2.9 *The most striking observations in the paper, namely the strong deformations and tether-like protrusions at high bacterial concentration, are also the least mechanistically explained. What sets the width, lifetime, and frequency of these protrusions? Are they true stable structures, or transient interfacial failures? Since the bacteria appear aligned along the tube axis, does local curvature promote accumulation and alignment, or do aligned bacteria generate the protrusions? This feedback is interesting, but not sufficiently discussed.*

Response: We thank the Reviewer for this insightful comment. The first type consists of strong deformations with widths of order several tens of micrometers, comparable to the correlation length of the collective bacterial flow (See Fig. 4a, S29, S31 and Movie S5-S6). We interpret these structures as a hydrodynamic response of the liquid–liquid interface to collective jet-like or vortical bacterial flows, governed by the competition between active normal stresses and capillary relaxation. Similar activity–capillarity competition has been discussed in simulations of active nematic droplets [Yeomans et.al, PRX 11, 021001 (2021)], although our system is an experimental bacterial-turbulence droplet rather than an explicitly modeled active nematic continuum system.

The second type consists of tether-like thin filaments, whose widths are comparable to one or a few bacterial cell widths (See Fig. 4d, S36 and Movie S8). Bacteria inside these filaments are locally aligned along the filament axis, suggesting that local packing, steric confinement, and orientational alignment are important. These filaments are distinct from membrane tubes observed in bacteria-filled vesicles ([Takatori et.al, Phys. Rev. Lett. 124, 158102 (2020)], [Nagard et al., PNAS 119, e2206096119 (2022)]): a membrane has bending elasticity and membrane tension that can stabilize slender tubular geometries, whereas our liquid–liquid interface has no bending rigidity. Thus, the observed filaments should not be viewed as equilibrium-stable tubes, but as transient nonequilibrium interfacial structures sustained by local bacterial forcing against capillary retraction.

Regarding the lifetime and occurrence frequency of protrusions, we note that both strong deformations and thin filaments are transient nonequilibrium structures. For strong deformations, Fig. 4c and Movie S6 show that highly elongated states, with aspect ratios exceeding π, can persist for several to tens of seconds before relaxing. The apparent occurrence frequency also depends on droplet size. Small droplets often alternate between elongated and nearly spherical shapes, as shown in Figs. S29, S34 and Movie S6, whereas larger droplets, with a larger interfacial area, can develop protrusions successively or simultaneously at different locations and therefore remain persistently deformed over the observation window, as shown in Figs. S33, S34 and Movie S7. For thin filaments, representative events shown in Movie S8 and Fig. S35

persist on the order of ten seconds before retracting or detaching from the main droplet body. Their occurrence is more heterogeneous from droplet to droplet, likely reflecting small variations in interfacial tension and bacterial volume fraction among different droplets.

Finally, we suggest the following tentative scenario to explain the formation of the thin filaments: orientation fluctuations of bacteria near the interface may generate a local curvature perturbation, which could then favor further bacterial accumulation and alignment, thereby reinforcing protrusion growth.

Revision: We have revised Sec. S5 of the Supplementary Information to clarify the characteristic widths, time scales, and occurrence behaviors of the two types of protrusions. In Secs. S5A and S5B, we added further discussion of their possible physical mechanisms. We also added a new Sec. S5C, together with Fig. S37, to compare the relationship and distinction between the strong deformations and the thin filaments. In particular, Fig. S37 shows the width distributions of the two structures, highlighting that they correspond to different length scales and likely arise from different activity-driven processes.

Comment 2.10 *Since much of the later dynamics is observed after sedimentation to the substrate, wall effects likely play an important role. The enhanced near-wall motility and faster coarsening may not only reflect active droplet physics, but also wall-induced effects and asymmetric confinement. This limitation should be discussed much more explicitly.*

Response: We thank the Reviewer for this important comment. We agree that wall effects and asymmetric confinement are important in the later-stage dynamics and should be stated more explicitly. In our experiments, bacteria-laden droplets sediment to the bottom substrate within about 10 min. Therefore, the subsequent motility, contact-line deformation, and coarsening should be interpreted as near-wall active-droplet dynamics rather than purely bulk behavior.

The later-stage behavior should thus be understood as the result of an interplay between bacterial activity and near-wall confinement. For passive droplets, sedimentation to the substrate leads to a stable contact line and essentially pinned droplets. By contrast, bacteria-laden droplets remain internally driven after sedimentation. The bacterial flows can deform the contact line, promote lateral motion along the substrate, and, at high bacterial concentration, even partially detach the droplet from the contact line. In this near-wall geometry, activity can therefore help droplets overcome substrate-induced pinning, increase droplet encounters, and accelerate coarsening.

We have revised the manuscript to clarify this limitation and interpretation. The enhanced motility and faster coarsening in Fig. 5 should not be interpreted as purely bulk active-droplet behavior. Rather, they reflect active stresses operating in the

presence of a nearby wall, where wall-induced confinement and contact-line effects are integral to the observed dynamics.

Revision: In paragraph 3 on page 6, we now explicitly emphasize the role of wall effects in the coarsening dynamics: "To quantify **this substrate-mediated coarsening** ... a final state dominated by a few large droplets (Fig. 1d, Fig. 5h)."

We further clarify this physical picture in the Discussion in paragraph 2 on page 7: " Our system further involves gravitational sedimentation and **a bottom substrate.** In the bulk, ... On the substrate, passive droplets tend to be pinned by stable contact lines, whereas high bacterial concentration droplets remain internally driven, allowing contact-line deformation, detachment from the substrate and lateral motion. Together, these effects provide an activity-assisted pathway for accelerated coarsening under gravity and confinement."

Comment 2.11 *Several important experimental details are missing or too brief. These include: the number of droplets analyzed, the number of independent experiments, uncertainty in bacterial volume fraction, temporal stability of motility, and whether oxygen depletion or cell viability were controlled. These controls are important, especially because the conclusions mainly rely on comparing only two concentrations.*

Response: We thank the reviewer for pointing out these important experimental details. We have now included additional information on the following points in the revised manuscript:

1. **The number of droplets and independent experiments:** We conducted nine independent experiments under the low-bacterial-density condition and analyzed approximately thirty droplets with different radii. We also conducted five independent experiments under the high-bacterial-density condition and measured the surface areas of eleven droplets, including six small droplets undergoing global elongation and five larger droplets exhibiting multiple protrusions. The widths of the six globally elongated droplets were measured in Fig. S37. Thin-filament widths were measured from five different droplets in Fig. S37. In three of these droplets, the bacterial orientations could be clearly resolved, allowing us to quantify the local orientational order.
2. **Uncertainty in bacterial volume fraction**: We analyzed five droplets for the low-bacterial-concentration condition and five droplets for the high-bacterial-concentration condition. From these measurements, we obtained a bacterial volume fraction of 21%±4% for the low-concentration state and 34%±3% for the high-concentration state, where the uncertainty represents the droplet-to-droplet variation across the measured samples.
3. **Cell motility and oxygen depletion**: We added glucose to the medium to support anaerobic respiration, ensuring that the bacteria remained viable and

motile even in the absence of oxygen. To keep bacteria alive in experiments, the samples were imaged within 1 h after preparation.

We believe these additional details will provide a more comprehensive understanding of the experimental controls and further strengthen the reliability of the conclusions drawn from the two concentration comparisons.

Revision: We have added several methodological clarifications and new analyses in the revised manuscript and Supplementary Information. In SI Sec. S1A, we explain that glucose can sustain bacterial motion even in the absence of oxygen. In SI Sec. S1D, we specify the imaging time window: To ensure sustained bacterial activity during imaging, the samples are imaged within 1 h after sample preparation. In the main text and SI Sec. S1F1, we describe how the bacterial volume fraction was measured and report the corresponding uncertainty. In SI Sec. S3A, we clarify the procedure for measuring the fluctuation spectra and state the number of droplets analyzed with low bacterial density. In SI Sec. S5A and B, we clarify the number of droplets analyzed with high bacterial density. In addition, we added Fig. S37 to show the width distributions of thick protrusions and thin filaments, and we provide the corresponding sample numbers in SI Sec. S5A.

Comment 2.12. *More broadly, the manuscript would benefit from a more careful discussion of previous work on active interfaces, active vesicles, and bacteria-driven deformations of soft boundaries. The distinction between a liquid–liquid interface and a membrane is important, but there are still conceptual overlaps that should be discussed more critically.*

Response: We thank the Reviewer for this helpful suggestion. First, active nematic liquid–liquid interfaces have demonstrated that activity can drive giant interfacial fluctuations and non-inertial propagating waves ([Adkins et al., Science 377, 768-772 (2022)],[Gulati et al., Soft Matter 20, 7703–7714 (2024)]). These studies are conceptually important for our work, but their mechanisms **are usually formulated in terms of coupling between interfacial shape and an active nematic director field**. Our system differs in both geometry and driving mechanism: the interface is a closed 3D droplet boundary rather than a flat or quasi-2D interface, and the driving comes from dense bacterial collective motion in the droplet interior. Accordingly, our low-activity wave dynamics are described by a hydrodynamic damping-compensation mechanism, in which active bacterial stresses produce an effective negative viscosity that can offset passive viscous damping.

Second, active vesicles and bacteria-filled vesicles have shown that internal active agents can deform soft boundaries and generate tether-like structures ([Takatori et al., Phys. Rev. Lett. 124, 158102 (2020)],[Vutukuri et al., Nature 586, 52–56(2020)],[Sciortino et al., Nature Physics 21, 954–962 (2025)],[Nagard et al., PNAS 119, e2206096119 (2022)]). This literature is conceptually relevant, but the boundary mechanics are different. **Vesicle membranes have bending elasticity and thermal**

membrane fluctuations, so activity often acts together with membrane bending modes and thermal-driven shape fluctuations. In contrast, our liquid–liquid interface has surface tension but no membrane bending elasticity, and thermally excited capillary fluctuations are negligible on the experimentally resolved length scales. Thus, the measured fluctuation spectra mainly reflect activity-induced interfacial dynamics, and the observed protrusions are transient liquid interfacial structures sustained by bacterial forcing rather than membrane tubes stabilized by bending elasticity.

Third, active-particle droplets, such as quasi-2D droplets enclosing Quincke rollers [Kokot et al., Communications Physics, 5, 91 (2022)] have shown nonequilibrium shape fluctuations, deformation, and self-propulsion. Compared with those systems, our droplets are fully liquid–liquid, three-dimensional compartments driven by biological active turbulence rather than by externally powered colloidal rollers. The quasi-2D geometry of Quincke-roller droplets also makes substrate friction and contact-line dynamics central to the shape dynamics. In our case, although wall effects are relevant after sedimentation, the interfacial fluctuations and shape dynamics arise from internally organized bacterial flows and their coupling to a capillary liquid–liquid boundary.

These discussions allow us to state the research gap more precisely. Our work addresses how collective bacterial turbulence inside a closed 3D liquid–liquid droplet couples to a purely capillary interface, producing hydrodynamic interfacial waves, multiscale protrusions, and activity-suppressed capillary breakup. This connects active-interface physics, active rheology, and capillary instability in a setting that is distinct from, but conceptually related to, previous work on active nematic interfaces, active-particle droplets, and active vesicles.

Revision: We revised the manuscript to emphasize that our system is a bacterial-turbulence-driven liquid-liquid interface. This point is now highlighted in the title, and the Abstract and Introduction now explicitly frame the central question as how a closed, three-dimensional liquid-liquid interface responds when driven internally by active turbulence.

In paragraph 2 on page 3, we added: “This activity-dominated crossover is distinct from passive equilibrium crossovers: unlike vesicle membranes, the liquid–liquid interface has surface tension but no bending rigidity, and thermal fluctuations are negligible compared with the activity-driven interfacial motion observed here.”

We revised the Discussion in paragraph 4 on page 6: “Interfacial waves in active nematics are typically attributed to nonreciprocal coupling between the nematic director and the interface. Traveling membrane deformations in active vesicles, on the other hand, occur on elastic boundaries, where membrane bending contributes to the restoring force. In contrast, our results suggest a continuum-level hydrodynamic mechanism in which activity-induced viscosity reduction in the bacterial phase compensates viscous damping from the passive phase.”

We have also expanded the Supplementary Information to clarify how our system differs from previous active-interface systems. In Sec. S3A2, we discuss the distinction between our fluctuation spectra and those reported in active vesicles and active droplets. In Sec. S3E, we provide a broader comparison between our experimental platform and other active-interface experimental systems. In Sec. S4G, we further compare the wave mechanism identified here with previously reported active-interface wave mechanisms.

Minor points 2.13 *Error bars and droplet-to-droplet variability are largely missing in the main text. Are the reported spectra based on single droplets, averaged over several droplets, or averaged over repeated experiments?*

Response: We thank the reviewer for pointing this out. In Fig. 2c, the reported spectra are not obtained by averaging over different droplets. Instead, each curve corresponds to one individual droplet. In total, we measured approximately 30 droplets with different radii and selected four representative droplets of different sizes for display in the main text. This choice was made because the fluctuation spectrum depends on droplet size, and averaging droplets with different radii would obscure this size dependence.

For each displayed droplet, one long experimental acquisition was divided into 30 statistically independent temporal segments. The fluctuation spectrum was calculated separately for each segment, and the mean spectrum was then obtained by averaging over these 30 segment-resolved spectra. The standard deviation among the 30 spectra is used to estimate the segment-to-segment fluctuation at each wave number.

Revision: We have added error bars in Fig. 2c and g. We also added the procedure for measuring the fluctuation spectra and error bars In SI Sec. S3A.

Minor points 2.14 *The discussion of the high-concentration regime would benefit from more quantitative analysis of local bacterial alignment inside the protrusions.*

Response and revision: We thank the Reviewer for this comment. We have added a quantitative analysis of the local bacterial alignment inside the protrusions in Sec. S5B. Specifically, we manually identified both the bacterial orientations and the local protrusion axis and then analyzed the angular relation between them. We examined 11 bacteria from three different protrusions and found that the angle between the bacterial orientation and the local protrusion direction is typically within $8\pm5°$. This result indicates that the bacteria inside the protrusions exhibit clear nematic alignment along the protrusion axis. We have now included this analysis in the revised manuscript and Supplementary Information.

Comment 2.15: *The manuscript also lacks a clear data and code availability statement.*

Response and revision: We have added Data availability and Code availability statements after the Methods section. We have also prepared a Source Data file containing the data underlying the plots in the main figures and Supplementary figures.

Reply to Reviewer #3:

Summary:

The manuscript presents experimental data and simulations that show waves and large deformations on the surfaces of fluid droplets due to bacterial motility induced active stresses. The work is well-executed, and the manuscript is overall interesting to read. However, there are major points outlined below that need to be addressed before the work can be published.

Comment 3.1 *As the manuscripts stands right now, most of the phenomena (effective non-inertial propagating waves and large dynamic protrusions) observed by them and even the analysis methodology can be found in several publications, many of which are cited in the manuscript. Therefore, it is crucial that authors state exactly what is the research gap that their study fills in and why that is non-trivial/important. For example, it is not enough to say that few experiments have realized closed liquid-liquid droplets that are actively deformed from within and that the presented experiment adds to this list. Title and abstract should reflect this level of detail.*

Response: We thank the reviewer for this comment. Active stresses can reshape soft biological boundaries, including cell membranes and liquid interfaces in biomolecular condensates. Previous studies have investigated active nematic coupled to flat liquid-liquid interfaces ([Adkins et al., Science 377, 768-772 (2022)], [Gulati et al., Soft Matter 20, 7703–7714 (2024)]), quasi-two-dimensional droplets enclosing Quincke rollers [Kokot et al., Commun. Phys. 5, 91 (2022)], as well as active agents encapsulated inside vesicles ([Vutukuri et al., Nature 586, 52-56 (2020)], [Sciortino et al., Nat. Phys. 21, 954-961 (2025)]). By contrast, how **a closed, three-dimensional liquid-liquid interface responds when driven from within by an active turbulent fluid** remains much less explored experimentally.

This gap is nontrivial for four reasons. **First, a liquid–liquid interface differs mechanically and thermally from a vesicle or membrane boundary** [Vutukuri et al., Nature 586, 52-56 (2020)],[Sciortino et al., Nat. Phys. 21, 954-961 (2025)]**.** It is governed by surface tension but has no membrane bending elasticity. Therefore, slender protrusions or filament-like structures are not stabilized by membrane elasticity and should remain susceptible to capillary retraction and breakup. Moreover, unlike vesicles, where thermal membrane fluctuations can be directly resolved, thermally excited capillary fluctuations in our liquid--liquid droplets are negligible on the length scales resolved in our experiments, as indicated by the absence of detectable shape fluctuations in passive droplets. Thus, the measured fluctuation spectra mainly reflect activity-induced interfacial dynamics rather than equilibrium thermal fluctuations.

Second, bacterial suspensions differ from active nematic interfaces in their relevant coarse-grained variables and rheology ([Adkins et al., Science 377, 768-772 (2022)], [Zhao et al., PNAS 121, e2410345121 (2024)],[Gulati et al., Soft Matter 20, 7703–7714 (2024)])**.** In such systems, interfacial deformation arises from the coupling between the interface and the nematic director field. In our system, the dense bacterial phase instead drives the interface through internally organized collective flows and active stresses. At the continuum level, this effect can be represented as an activity-induced reduction of viscous damping. The resulting wave mechanism therefore has a hydrodynamic origin, rather than relying on nonreciprocal coupling between the interface and an explicit nematic director field. **Third, our system also differs from quasi-two-dimensional active droplets enclosing Quincke rollers** [Kokot et al., Communications Physics 5, 91 (2022)]. In Quincke-roller droplets, the active units are electrically driven colloidal rollers confined in a quasi-two-dimensional geometry. In contrast, our droplets are closed, three-dimensional liquid-liquid compartments driven by dense bacterial collective motion. Thus, the two systems differ both in dimensionality and in the physical origin of activity. **Fourth, our system further differs from the above examples because it involves a population of coalescence-capable liquid-liquid droplets, rather than an isolated active boundary or a single confined active droplet.** In this setting, activity not only drives interfacial waves and protrusions on individual droplets, but also promotes droplet motion, encounters, fusion, and coarsening. This population-level evolution is absent in flat-interface geometries and strongly constrained in vesicles by membrane topology. Therefore, our work connects active interfacial dynamics at the level of single droplets to the collective evolution of an active liquid-liquid phase-separated system.

Accordingly, we now frame the novelty of the work around three connected findings. **First, at low bacterial concentration**, we identify a hydrodynamic route to active interfacial waves in which active bacterial stresses compensate viscous damping from the surrounding passive phase. The novelty is therefore not simply the existence of propagating waves, but the identification of a damping-compensation mechanism associated with bacterial active rheology in a closed liquid–liquid droplet. **Second, at high bacterial concentration**, we observe multiscale giant interfacial deformations of a fully fluid interface: broad protrusions associated with collective bacterial flows coexist with much thinner liquid filaments associated with local bacterial alignment and particulate effects. **Third, our results show that bacterial activity accelerates droplet coarsening by helping droplets escape near-wall confinement.** Weakly active or passive droplets remain more strongly constrained by the bottom surface, whereas highly active droplets deform, move, and encounter neighboring droplets more readily. This promotes fusion and enhances coarsening, linking microscopic bacterial collective motion to the mesoscale evolution of the droplet population.

We have revised the title, abstract, introduction, and discussion to reflect this more precise framing. The revised manuscript no longer presents the work as simply another example of activity-induced droplet deformation. Instead, it emphasizes the coupling

between bacterial turbulence, active rheology, and capillary interfacial mechanics in a closed three-dimensional liquid–liquid droplet.

Revision: We have revised the manuscript to more clearly emphasize the distinct physical setting of our work. The title has been changed to "Bacterial turbulence drives interfacial waves and shape dynamics in phase-separated droplets."

In the Abstract, we added the statement, "Yet how internally generated active stresses are transmitted to capillary interfaces to reshape three-dimensional droplet dynamics remains poorly understood. Here, we encapsulate dense suspensions of motile bacteria inside phase-separated aqueous droplets, creating a closed droplet whose interface is driven from within by bacterial turbulence."

In the Introduction, we similarly clarified the central question by stating that "However, it remains experimentally unclear how a closed, three-dimensional liquid – liquid interface responds when driven internally by active turbulence".

We have also expanded the Supplementary Information to clarify how our system differs from previous active-interface systems. In Sec. S3A.2, we discuss the distinction between our fluctuation spectra and those reported in active vesicles and active droplets. In Sec. S3E, we provide a broader comparison between our experimental platform and other active-interface experimental systems. In Sec. S4G, we further compare the wave mechanism identified here with previously reported active-interface wave mechanisms.

Comment 3.2 *The discussion section states "Whereas interfacial waves in active nematics are typically attributed to the nonreciprocal coupling between the nematic director and the interface [16, 58], our results reveal a hydrodynamic mechanism based on viscosity matching between active and passive fluids." I agree with the authors that their theory based on viscosity mismatch explains the presented data well. But the negative viscosity of the active fluid in their model arises primarily due to the formation of an active nematic phase. So, how are the authors so sure that a mechanism based on non-reciprocal coupling between the nematic director and the interface will not be good enough to explain the data. In other words, multiple models can explain the same set of data in which case it becomes important to address the physical considerations to rule out some of them.*

Response: We thank the Reviewer for this thoughtful comment. We agree that orientational correlations in the bacterial suspension may contribute microscopically to the negative effective viscosity used in our coarse-grained model. We therefore do not intend to claim that director-based physics is irrelevant, nor that the present data uniquely rule out all possible models based on nonreciprocal coupling between orientational order and the interface. Our conclusion is more limited: the observed low-amplitude interfacial waves are quantitatively consistent with, and can be

parsimoniously described by, a hydrodynamic damping-compensation mechanism without introducing an explicit interfacial nematic director field.

There are several physical reasons for this modeling choice. First, the interfacial waves analyzed here occur in the low-bacterial-volume-fraction regime, where the droplets remain approximately spherical and we do not observe persistent nematic ordering localized at the droplet interface within our imaging resolution. The clearest bacterial alignment appears instead in the high-bacterial-volume-fraction thin-filament regime, where bacteria align along the filament axis. We interpret this alignment as a localized confinement-induced effect associated with bacterial-scale protrusions, rather than as the defining dynamical variable of the low-amplitude wave state.

Second, the damping-compensation picture explains several observations within a single framework. It accounts for the appearance of propagating modes mainly at long wavelengths, the disappearance of wave peaks above a cutoff wavenumber k_c, the relation between this cutoff and the active-flow correlation length ℓ_v, and the occurrence of similar wave dynamics in both droplet and flat-interface simulations. In this description, collective bacterial motion modifies the effective viscous response of the droplet phase. When the active contribution nearly compensates the viscous damping from the surrounding passive phase, selected long-wavelength interfacial modes become effectively underdamped even in the low-Reynolds-number regime.

At the same time, our interpretation does not exclude orientational physics at the microscopic level. For extensile bacteria, local orientational correlations and shear-induced alignment can generate active stresses that oppose passive viscous stresses. After coarse graining, these effects can appear as an activity-dependent reduction of the effective viscosity. Thus, orientational correlations may contribute to the negative effective viscosity in the TTSH-type description, but in our minimal model they enter through the bulk active rheology of the bacterial suspension rather than through an explicitly modeled nonreciprocal coupling between an interfacial nematic director and the interface.

Revision: In the revised manuscript, we have softened this statement in paragraph 5 on page 6: "our results suggest a hydrodynamic mechanism, at the continuum level, based on viscosity matching between active and passive fluids."

In paragraph 2 on page 3, we added:"This activity-dominated crossover is distinct from passive equilibrium crossovers: unlike vesicle membranes, the liquid–liquid interface has surface tension but no bending rigidity, and thermal fluctuations are negligible compared with the activity-driven interfacial motion observed here."

We revised the Discussion in paragraph 4 on page 6: "Interfacial waves in active nematics are typically attributed to nonreciprocal coupling between the nematic director and the interface. Traveling membrane deformations in active vesicles, on the other hand, occur on elastic boundaries, where membrane bending contributes to the restoring

force. In contrast, our results suggest a continuum-level hydrodynamic mechanism in which activity-induced viscosity reduction in the bacterial phase compensates viscous damping from the passive phase."

We have also added a new subsection in the Supplementary Information Sec. S4G, "Comparison with other active-interface wave mechanisms", to clarify this distinction. The revised text explains that our model does not rule out microscopic orientational correlations but treats their effect at the coarse-grained level through bulk active rheology and damping compensation, rather than through an explicit interfacial director field.

Comment 3.3 *Fig. 2c,g show that S(k) scales as k^{-2} for $k < k_c$ and k^{-4} for $k > k_c$. Usually, one associates k^{-2} scaling as equilibrium capillary waves (Science, 304, 847 (2004)). So it is non-intuitive to associate the whole spectrum regardless of scaling laws as associated with waves generated by active stresses. Other works on droplets with active particles inside them have only seen k^{-4} scaling (Communications Physics, 5, 91 (2022)). Can the authors comment on this?*

Response: We thank the Reviewer for this comment. We agree that a k^{-2} scaling alone should not be taken as direct evidence for active waves, since the same scaling can also appear in equilibrium capillary-wave spectra. In our system, the surface tension $\gamma_0 \sim 7.5 \pm 1.7$ μN/m at T≃20°C, therefore the thermal capillary length scale is $\sqrt{\frac{k_B T}{\gamma_0}} \sim 23$ nm, which is far below the micrometer-scale deformations observed in active droplets and below our experimental resolution. Therefore, the measurable interfacial fluctuations in bacteria-containing droplets are dominated by active stresses rather than by thermal capillary fluctuations.

Our results suggest that the crossover in the static spectrum occurs at the active-flow length scale, $k\ell_v \sim 1$. Modes with $k\ell_v < 1$, corresponding to wavelengths larger than the bacterial-flow correlation length, show the k^{-2} scaling. Modes with $k\ell_v > 1$, corresponding to wavelengths smaller than the active-flow scale, show a steeper k^{-4} decay. This interpretation also provides a possible way to understand why such a crossover was not resolved in the active-particle droplet study in [Kokot et al., Communications Physics, 5, 91 (2022)]. In that quasi-two-dimensional Quincke-roller droplet, the reported parameters suggest $\ell_v/R_0 \approx 0.3 - 0.75$ depending on the dynamical state. . Since the lowest nontrivial interfacial mode is of order $\mathrm{k} \approx 2/R_0$, the accessible spectrum would start near $\mathrm{k}_{\mathrm{min}}\ell_v \approx 0.6 - 1.5$, i.e. close to or already on the high-k side of the putative crossover. Thus, we speculate that if a similar system could be realized with larger droplets, or more generally with a smaller ratio ℓ_v/R_0, lower-k modes with $k\ell_v < 1$ might become accessible and a scaling transition could potentially be observed. We note, however, that this comparison should be made cautiously, because that system is a quasi-two-dimensional Quincke-roller droplet affected by substrate friction, contact-line motion, and possible pinning, whereas our

system is a three-dimensional liquid-liquid droplet driven by dense bacterial collective motion.

Revision: We added comment in SI Sec. S3A2 to state the system size appears to be comparable to the correlation length of the internal active flow, which may limit the accessible scale separation between the droplet size and the collective-flow length scale in [Kokot et al., Communications Physics, 5, 91 (2022)].

Comment 3.4 *The authors mention: "As k increases, Sn(k, ω) no longer displays distinct wave peaks. This transition occurs at a kc , marked by the dashed line in Fig. 2d, which approximately corresponds to the scale of the velocity correlation length ℓv." This suggests that when the correlation length is lower (higher k), these wave peaks would extend even more (FIG. S18). But a lower correlation length implies weaker collective behaviour. Since the authors claim that the scale-dependent fluctuations arise from collective bacterial flows, this seems like a contradiction. Is collective behaviour the sole cause of the propagating waves?*
a) If there were no collective behaviour but random active stresses, will there be any enhanced fluctuations or propagating waves?
b) Can the authors clarify what would happen if the correlation length were very low (lower than shown in FIG S18)?
c) In FIG. S18, it would be appropriate to include snapshots of the flow fields.

Response and revision: We thank the Reviewer for raising this important point. In our interpretation, ℓ_v sets **the spatial coherence scale** of the collective bacterial flow, but it is not by itself sufficient to generate propagating interfacial modes. The appearance of wave peaks in $S_n(k,\omega)$ requires two ingredients: spatially and temporally coherent active stresses generated by collective bacterial flows, and sufficiently strong negative effective damping to compensate passive viscous dissipation. In the TTSH description of bacterial turbulence, the fourth-order damping term suppresses the negative-viscosity contribution at short wavelengths, such that, the active phase can compensate passive damping only over a finite range of wavenumbers, with an approximate cutoff $k_c \sim 1/\ell_v$.

Thus, a smaller ℓ_v should not be interpreted simply as weaker collective behavior. It means that the coherent active-flow structures occur on a smaller length scale, which shifts the cutoff toward larger k. As shown in Fig. S25, when ℓ_v is varied while the system remains in the collective active-flow regime, the wave-bearing range shifts approximately with $1/\ell_v$. This does not imply that weaker collective behavior produces stronger waves; rather, it shows that ℓ_v controls the wavelength range over which active damping compensation can operate.

To further address the Reviewer's question, we added two control simulations to the Supplementary Information. First, we replaced the coherent collective active flow by random active forcing. As shown in Fig. S26, the dynamic structural factor shows a peak at ω=0, indicating there are no propagating wave signals. This confirms that

collective behavior is not merely a source of stronger noise but provides the spatial-temporal coherence needed for propagating modes.

Second, we performed simulations with very small velocity correlation length. As shown in Fig. S24, reducing ℓ_v alone does not necessarily eliminate the waves; instead, it shifts the cutoff to higher k, as expected from $k_c \sim 1/\ell_v$, provided that the active-flow amplitude and negative effective damping remain sufficiently strong. However, we also clarify that this interpretation has a physical limit. If ℓ_v becomes comparable to the bacterial length scale, the interfacial thickness, or the numerical grid scale, the continuum TTSH model should no longer be interpreted literally. In real bacterial suspensions, an extremely small ℓ_v may also be accompanied by weaker orientational correlations or a reduced active-stress amplitude, in which case the wave peaks would weaken or disappear.

Following the Reviewer's suggestion, we have also added representative snapshots of the instantaneous flow fields to Fig. S24 (S18 in previous version). These snapshots directly show that changing ℓ_v changes the characteristic size of the coherent vortical structures: smaller ℓ_v corresponds to smaller active-flow structures and therefore shifts the wave cutoff toward larger k. Importantly, the flow fields remain collective and coherent in these simulations; thus, the change in ℓ_v should be interpreted as a change in the spatial scale of collective active forcing, not simply as a reduction of collective behavior.

Comment 3.5 *The authors have mentioned that they used the pre-separated PEG–dextran phases (PEG rich and dextran rich phases) and then mixed them with the bacterial suspensions. Can they shed some light on why this is being done, instead of directly mixing PEG, dextran and bacterial cells?*

Response: We used pre-separated PEG-rich and dextran-rich phases primarily to control the volume ratio between the PEG-rich phase, the dextran-rich phase, and the bacterial suspension. Once the PEG–dextran system had fully phase separated, the two equilibrated phases were collected separately. We could then prepare samples by mixing prescribed volumes of PEG-rich phase, dextran-rich phase, and bacterial suspension. This procedure gives direct control over the initial sample composition and improves reproducibility. Directly mixing PEG, dextran, and bacterial cells would couple the final phase volumes to the phase separating process, making the composition more sensitive to mixing history and equilibration time.

Revision: In Sec. S1D, we add: We generate active, phase-separated droplets by introducing the dense bacterial suspension into the pre-separated PEG-dextran phases. This procedure allows us to prescribe the volume ratio of PEG-rich phase, dextran-rich phase, and bacterial suspension directly, improving the reproducibility of the initial sample composition compared with directly mixing PEG, dextran, and bacteria before phase separation.

Minor Comment 3.6 *The labels in the insets of Figs. 2(g) and 3(c) are too faint to read.*

Response and Revision: The labels in the insets of Figs. 2(g) and 3(c) have been enlarged and darkened for better visibility.

Minor Comment 3.7 *Figure 2 caption for panel (h) states R = 32. Are the units missing or was this some dimensionless radius being specified?*

Response: We thank the Reviewer for pointing this out. The radius R=32 in Fig. 2(h) denotes a dimensionless simulation radius, not a physical length with missing units. This scaling procedure follows the spirit of [Dunkel et al. PRL 110, 228102 (2013)], where simulation units are related to experiments by matching the characteristic velocity-correlation length and collective-flow speed.

Revision: We have revised the caption of Fig. 2 to explicitly state that the simulation radii and velocities are given in nondimensional simulation units. We have also revised the Supplementary Information to clarify the unit mapping used for simulations. In the SI Sec. S2, we state explicitly that simulation units are related to experiments by matching the characteristic velocity-correlation length and collective-flow speed.

Reply to Reviewer #4:

I co-reviewed this manuscript with one of the reviewers who provided the listed reports. This is part of the Nature Communications initiative to facilitate training in peer review and to provide appropriate recogniΘon for Early Career Researchers who co-review manuscripts.

Response: We thank the Reviewer for co-reviewing the manuscript and for the participation in the review process.
