# Supplementary material for: Bacterial turbulence drives interfacial waves and shape dynamics in phase-separated droplets
Source: Nat Commun. 2026 Jul 14;17:8661. doi: 10.1038/s41467-026-75497-5 (PMC13490374; doi:10.1038/s41467-026-75497-5)
Supplement: Supplementary file 3 [file 41467_2026_75497_MOESM1_ESM.pdf]

Bacterial turbulence drives interfacial waves and shape dynamics in phase-separated droplets

Kan Chang,[1,*] Yulin Li,[2,*] Ming Yuan,[1] Masaki Sano,[1,3] Zhihong You,[2,†] and H. P. Zhang[1,‡]

[1]*School of Physics and Astronomy, Institute of Natural Sciences and MOE-LSC, Shanghai Jiao Tong University, Shanghai 200240, China*

[2]*Fujian Provincial Key Laboratory for Soft Functional Materials Research, Research Institute for Biomimetics and Soft Matter, Department of Physics, Xiamen University, Xiamen, Fujian 361005, China*

[3]*Universal Biology Institute, Graduate School of Science, The University of Tokyo, Tokyo 113-0033, Japan*

(Dated: June 30, 2026)

CONTENTS

Section S1. Experimental Methods 4

A. Bacterial cultivation 4

B. Preparation of the phase-separated system 4

C. Physical properties of the phase-separated system 4

D. Formation of active droplets 6

E. Confocal imaging and data acquisition 7

F. Volume fraction measurement and physical states of bacterial droplets at two concentrations 7

1. Estimation of bacterial volume fraction 7
2. Shapes of bacterial droplets at two concentrations 8
3. Velocity distribution of bacterial motion at different bacterial volume fractions 9

Section S2. Continuum model and numerical implementation 11

* These authors contributed equally

† Corresponding author: zhyou@xmu.edu.cn

‡ Corresponding author: Hepeng_zhang@sjtu.edu.cn

A. Continuum model for phase separation and active flows 11
B. Numerical implementation 12
C. Parameter selection 13

Section S3. Quantification of droplet dynamics 14
A. Fluctuation spectra 15
1. Extraction of droplet interface and contour 15
2. Calculation of static fluctuation spectra 16
3. Spherical harmonic decomposition of 3D droplet shape 19
4. Calculation of dynamic structure factor 20
B. 3D morphological analysis 22
C. Quantification of droplet–substrate interaction 23
D. Quantification of coarsening dynamics 23
1. Definition of bulk-droplet volume fraction 23
2. Measurement of correlation length during coarsening 25
3. Characterizing the role of bacterial concentration on coarsening dynamics 25
E. Comparison with previous active-interface experimental systems 28

Section S4. Supplementary results for droplets with low bacterial concentration 29
A. Flow field in low-bacterial-density droplets 29
B. Mode analysis under initial perturbations 30
C. Effect of activity on the dynamic structure factor 31
D. Effect of correlation length on the dynamic structure factor 34
E. Control simulations with Ornstein-Uhlenbeck active stresses 35
F. Theory of activity-induced propagating waves on a fluctuating interface 36
G. Comparison with other active-interface wave mechanisms 42

Section S5. Supplementary results for droplets with high bacterial concentration 43
A. Global and local deformations of droplets with high bacterial concentration 44
B. Formation and dynamics of thin filaments 46
C. Comparison of the two protrusive structures 48

Section S6. List of symbols and parameters 51

References 54

Section S1. EXPERIMENTAL METHODS

This section describes the experimental system used in this study, including bacterial preparation, PEG–dextran phase separation, droplet formation, and confocal imaging. Each subsection provides the parameters required to reproduce the experiment and the physical interpretations behind them.

A. Bacterial cultivation

E. coli BW25113 is used as the active particle. The bacteria are cultured overnight at 37°C in Terrific Broth (TB), which contains tryptone (1.2% w/v), yeast extract (2.4% w/v), and glycerol (0.4% v/v). The saturated culture is diluted 1:100 in fresh TB medium and incubated at 30°C for 5 hours. After incubation, 3 mL of culture is harvested by centrifugation at 800 g for 5 minutes at room temperature. The cells are then washed twice with motility buffer, which contains 0.01 M potassium phosphate, 0.067 M NaCl, 10^{-4} M EDTA, 0.5% glucose, and pH 7.0. Glucose can sustain bacterial motion even in the absence of oxygen [1]. The final bacterial suspension is obtained after removing the supernatant.

B. Preparation of the phase-separated system

We prepare aqueous two-phase mixtures by dissolving dextran (DEX; Mw 20 kDa, Adamas) and polyethylene glycol (PEG; Mw 8 kDa, Macklin) in motility buffer at equal initial concentrations ($c_{\text{PEG}} = c_{\text{DEX}} = 7.5\%$ w/w). To visualize the dextran-rich phase, we add fluorescein-labeled dextran (D1844, 40 kDa, ThermoFisher Scientific) at a concentration below 0.1% (w/w). After thorough mixing, we centrifuge the solution at 800 g for 5 min to initiate phase separation, and we leave it undisturbed for 24 h to reach equilibrium. We then extract the upper PEG-rich phase and the lower dextran-rich phase and store them separately for subsequent use.

C. Physical properties of the phase-separated system

We characterize the physical properties of the separated phases to establish the passive fluid environment of the droplets. At 20 °C, the PEG-rich and dextran-rich phases have

densities of 1.037 g/mL and 1.074 g/mL, respectively. Their viscosities are 6.7 mPa s for the PEG-rich phase and 14.3 mPa s for the dextran-rich phase. The interfacial tension of the droplet is determined by balancing interfacial and hydrostatic forces. For an axisymmetric liquid droplet, the pressure difference Δp across the interface at height z is governed by the Young-Laplace equation:

$$\gamma_0 \left(\frac{d\theta}{ds} + \frac{\sin\theta}{r} \right) = \Delta p_0 + \Delta\rho g z, \tag{1}$$

where s is the arc length, r is the radial distance from the symmetry axis, and θ is the local tangent angle of the interface. Here z is measured downward from the droplet apex. Following the ADSA notation of del Río and Neumann [2], b denotes the curvature at the droplet apex, rather than the apex radius of curvature. Thus, the apex pressure jump is $\Delta p_0 = 2\gamma_0 b$.

Using the capillary length $L_c = \sqrt{\frac{\gamma_0}{\Delta\rho g}}$, the Young-Laplace equation can be rewritten as

$$\frac{d\theta}{ds} = 2b + \frac{z}{L_c^2} - \frac{\sin\theta}{r}. \tag{2}$$

Together with $\frac{dr}{ds} = \cos\theta$, $\frac{dz}{ds} = \sin\theta$, these equations define the theoretical axisymmetric droplet profile.

We numerically integrate the equations and optimize the fitting parameters b and L_c to minimize the deviation between the theoretical profile and the experimentally extracted droplet contour, following the Axisymmetric Drop Shape Analysis (ADSA) framework established by del Río and Neumann [2]. Once L_c is determined from the best-fit model, the interfacial tension is calculated as

$$\gamma_0 = L_c^2 \Delta\rho g. \tag{3}$$

In the experiment, we determine the interfacial tension of large droplets by fitting their side-view profiles, as shown in Supplementary Figure 1. The interfacial tension is obtained from fits to the droplet contours, and a representative fitting result is shown as the red line in Supplementary Figure 1. In total, we measure eight different droplets and obtain an interfacial tension of 7.5 ± 1.7 μN/m. Therefore, at room temperature, the thermal capillary fluctuation scale can be estimated as

$$\sqrt{\frac{k_{\mathrm{B}}T}{\gamma_0}} \simeq 23 \text{ nm}.$$

This length scale is far below the micrometer-scale deformations observed in active droplets and also below our experimental resolution. Consistently, bacteria-free droplets of comparable size show negligible interfacial fluctuations.

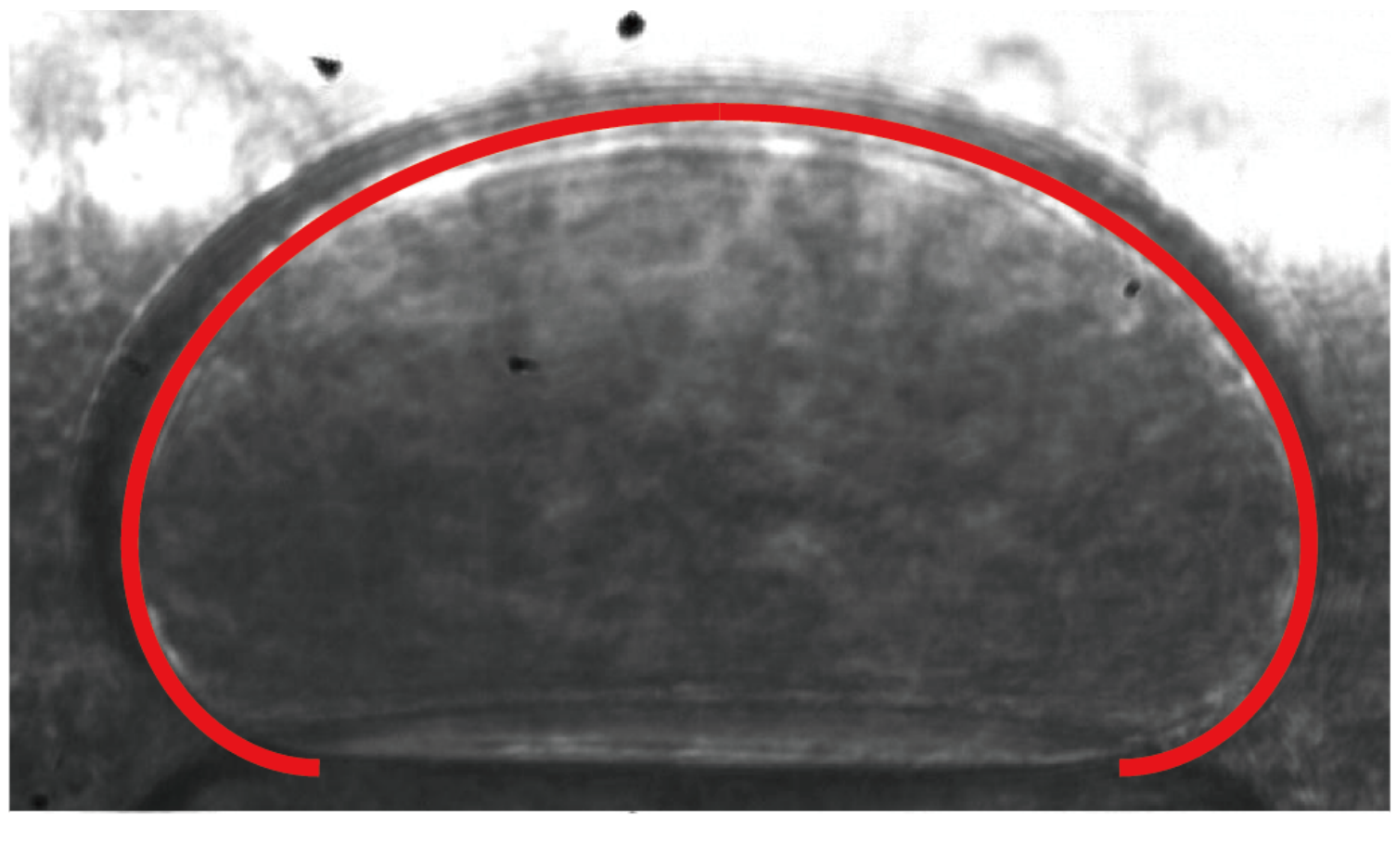

Supplementary Figure 1. Measurement of the interfacial tension. Side-view image of a droplet and the corresponding contour fit used to determine the interfacial tension (red line). The scale bar corresponds to 100 μm.

D. Formation of active droplets

We generate active, phase-separated droplets by introducing the dense bacterial suspension into the pre-separated PEG-dextran phases. This procedure allows us to prescribe the volume ratio of PEG-rich phase, dextran-rich phase, and bacterial suspension directly, improving the reproducibility of the initial sample composition compared with directly mixing PEG, dextran, and bacteria before phase separation. Each mixture contains 200 μL of PEG-rich phase, 10 μL of dextran-rich phase, and either 5 μL (low volume fraction) or 8 μL(high volume fraction) of concentrated *E. coli* suspension. We shake the mixture for 1 min to facilitate complete partitioning of bacteria into the dextran-rich phase.

We seal the active mixtures in a circular chamber (radius 2.5 mm) formed by double-sided adhesive tape between two glass coverslips. For droplets smaller than 100 μm, we use a chamber height of 200 μm. For droplets larger than 100 μm, we increase the chamber height to 600 μm to prevent the droplets from contacting the upper glass surface. To ensure sustained bacterial activity during imaging, the samples are imaged within 1 h after sample

preparation.

E. Confocal imaging and data acquisition

We image all samples using a Nikon CSU-W1 spinning-disk confocal microscope. Flow fields and droplet contours of droplets with radii below 100 μm are acquired with a 60$\times$ water-immersion objective, whereas droplets larger than 100 μm are imaged with a 20$\times$ objective.

Three-dimensional reconstructions of individual droplets are obtained using the 60$\times$ water-immersion objective. For 1200×1200-pixel frames, the scanning speed is 25 μm/s per frame, for 704×704-pixel frames, the scanning speed increases to 50 μm/s.

Coarsening measurements are performed using a 40$\times$ water-immersion objective. Each full 3D scan requires approximately 10 s, and the sample is scanned twice per minute to resolve the temporal evolution.

All images are preprocessed using the `denoise.ai` module in NIS-Elements to enhance signal quality. In all 3D renderings, scale bars correspond to the spatial scale of the image plane closest to the viewing direction. Grayscale intensity reflects dextran-488 fluorescence, marking the dextran-rich phase.

F. Volume fraction measurement and physical states of bacterial droplets at two concentrations

1. Estimation of bacterial volume fraction

To measure the bacterial volume fraction inside droplets under different conditions, volume fractions are estimated from figures acquired in the 240$\times$ SORA mode by manual counting. Due to the relatively high magnification, bacteria and droplets can be clearly distinguished.

We show two representative droplets in Supplementary Figure 2(a-b), the dextran phase appears bright, while the bacteria are relatively dark. We manually identify all pixels corresponding to bacteria and label them in each confocal scanned picture (Supplementary Figure 2(a-b)), allowing us to calculate the volume of bacteria.

We measure five independent droplets in each case and find that, for low bacteria concentration, the volume fraction is $21\% \pm 4\%$, while for high bacteria concentration, the volume fraction is $34\% \pm 3\%$.

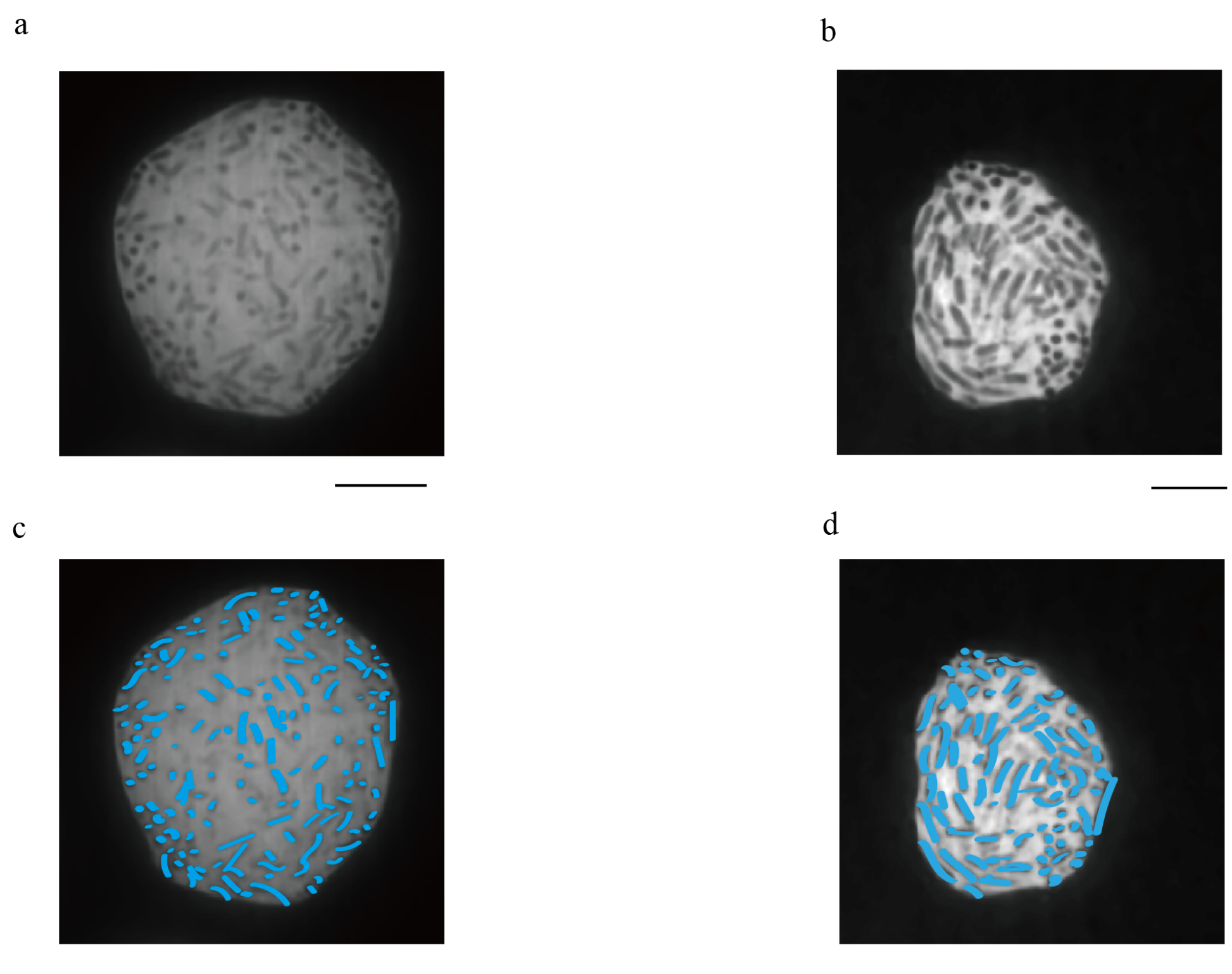

Supplementary Figure 2. (a,b) Images of bacterial droplets acquired in the $240\times$ SORA mode, with (a) low bacterial concentration and (b) high bacterial concentration. The bright region corresponds to the droplet, while the slightly darker regions inside the droplet correspond to bacteria. The scale bar is 10μm. (c,d) Manually identified bacterial regions.

2. Shapes of bacterial droplets at two concentrations

In Supplementary Figure 3, we present droplets at different bacterial concentrations. As shown in Supplementary Figure 3(a), droplets at low bacterial concentration remain mostly close to spherical. In contrast, at high bacterial concentration, collective bacterial motion drives strong droplet deformations. These include (b) the elongation of a small droplet, (c) a larger droplet displaying multiple protrusions, and (d) slender filaments extending from the droplet body. The left panels show top-view 3D fluorescence images.

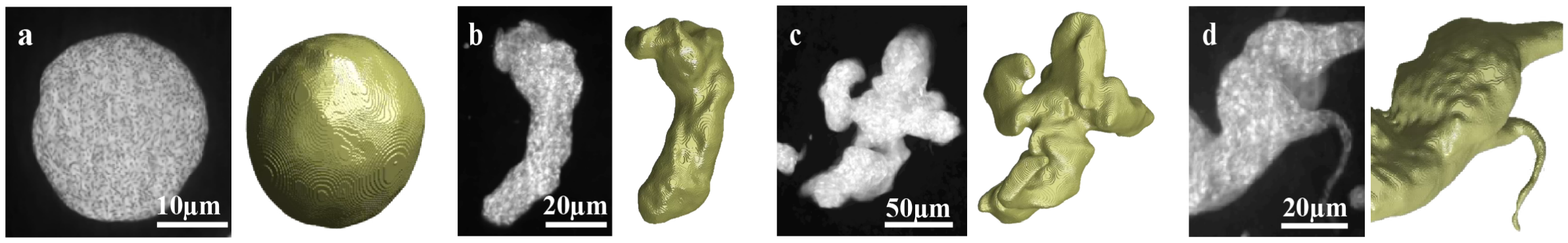

Supplementary Figure 3. Morphologies of droplets under different bacterial concentrations. (a) A low-bacterial-concentration droplet. Left: 2D equatorial cross-section showing a nearly spherical morphology. Right: 3D isosurface rendering. (b–d) High-bacterial-concentration droplets. (b) A small droplet exhibiting global elongation; (c) A larger droplet displaying multiple protrusions; (d) Slender filaments extending from the droplet body. Left: top-view 3D fluorescence image. Right: corresponding 3D isosurface rendering. In all fluorescence panels, grayscale intensity reflects dextran-488 fluorescence, indicating the dextran-rich phase.

3. Velocity distribution of bacterial motion at different bacterial volume fractions

In both the low- and high-bacterial-concentration states, the droplets exhibit persistent collective bacterial motion with vortical and jet-like structures, which we refer to as a coarse-grained bacterial-turbulence-like state. To quantify these internal dynamics, we perform PIV analysis with the open-source software *Fluere*.

As shown in Supplementary Figure 4, we measure the speed distributions over 20 s for droplets at low and high bacterial volume fractions. At the lower bacterial volume fraction, the speeds remain relatively moderate, with a mean speed of approximately 3 μm/s. At the higher bacterial volume fraction, the mean speed increases to approximately 4 μm/s, and the distribution develops a stronger high-speed tail, with maximum speeds reaching 15-20 μm/s. These high-speed events are associated with jet-like collective motion and pronounced protrusions during large droplet deformations, as shown in Fig. 4a of the main text.

As a reference, we also measure the swimming-speed distribution of individual bacteria in the dilute limit in the DEX phase. More than 900 single-cell trajectories are analyzed, and the resulting distribution is shown in the inset of Supplementary Figure 4. The mean single-cell swimming speed is approximately 2.5 μm/s, lower than the mean collective-flow speed in the bacterial droplets. Both the individual and collective speeds are relatively low

because the bacteria swim in a polymer-rich aqueous medium, whose viscosity is higher than that of standard motility buffer.

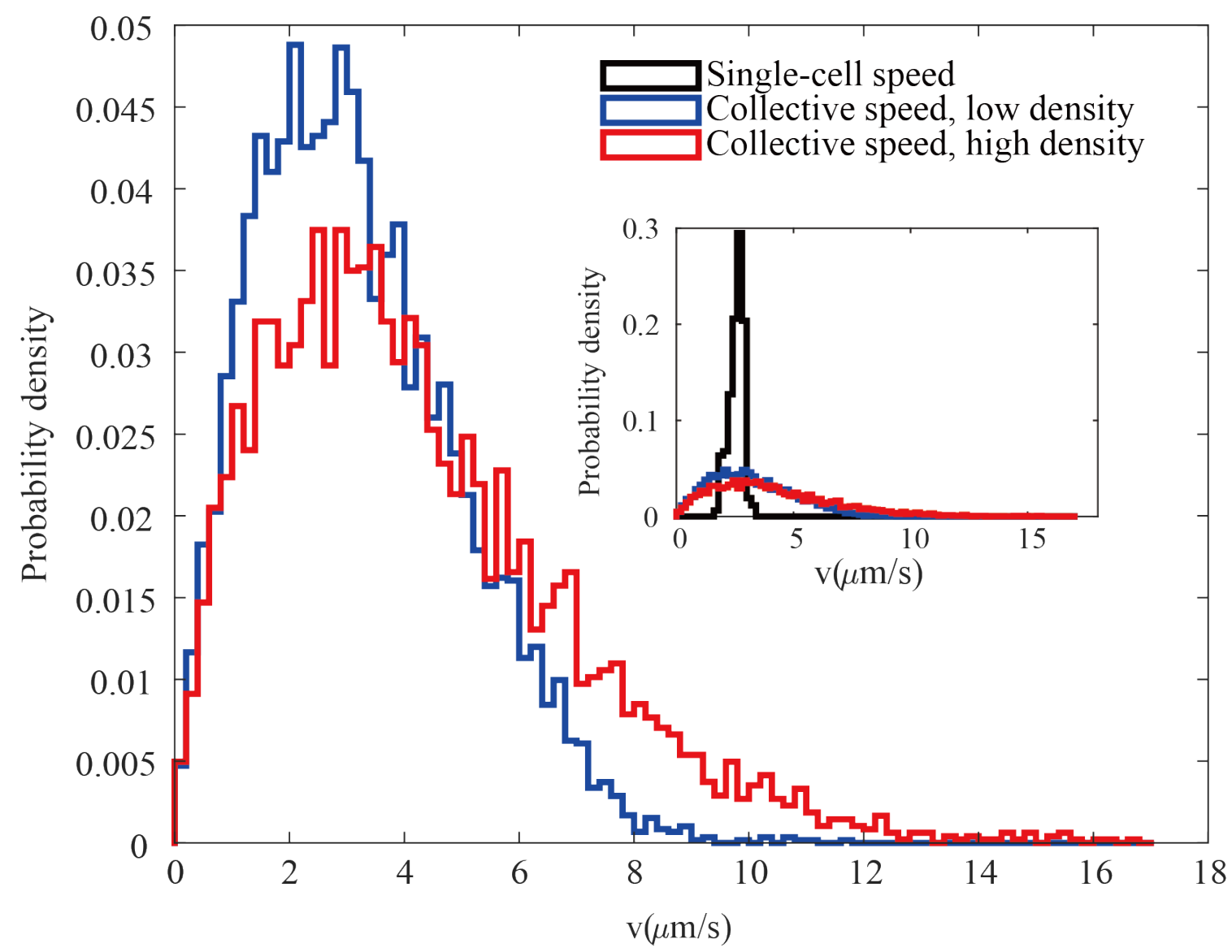

Supplementary Figure 4. Speed distributions of collective bacterial motion at different bacterial concentrations. Inset: speed distribution of individual swimming bacteria, shown for comparison with the collective-flow speeds.

We emphasize that bacterial concentration should not be interpreted as the swimming activity of an individual bacterium but rather as the coarse-grained active stress. In a coarse-grained description, this can be expressed schematically as $\sigma^a_{ij} \sim -\phi_b S Q_{ij}$, S is the stresslet strength of an individual swimmer, and Q_{ij} represents the coarse-grained local orientational order tensor. Thus, even if the single-bacterium swimming speed does not increase, the collective active stress exerted on the liquid–liquid interface can increase with bacterial loading.

In the numerical model, we therefore use the terms low-activity and high-activity droplets as an effective coarse-grained description of the two bacterial-loading conditions. Here, "activity" refers to the collective active stress or energy-injection density of the bacterial suspension as a whole, rather than solely to the swimming speed or motor activity of an individual bacterium. Operationally, the low- and high-activity cases in the model correspond to the low- and high-bacterial-volume-fraction droplets in the experiments, respectively.

Notably, even in the high-bacterial-volume-fraction condition, the droplet interior does not exhibit partial kinetic arrest. The high value used in our experiments, $\phi_b \simeq 34\%$, is still

far below the packing fractions associated with glass-like or jammed states where transitions have been reported near $\phi \simeq 0.85$-0.88 [3].

Section S2. CONTINUUM MODEL AND NUMERICAL IMPLEMENTATION

This section introduces the continuum description of the active–passive mixture, including the model formulation, parameter values, and implementation details for both 2D and 3D simulations.

A. Continuum model for phase separation and active flows

We model the active mixture as a phase-separating liquid mixture described by a phase field ϕ and a velocity field $\mathbf{v}$. The phase field has values between $\phi = 0$ (passive phase) and $\phi = 1$ (active phase), separated by interfaces where ϕ varies continuously from 0 to 1. The dynamics of ϕ is governed by the Cahn-Hilliard equation with advection [4]

$$\partial_t \phi + \mathbf{v} \cdot \nabla \phi = \Gamma_\phi \nabla^2 \mu. \tag{4}$$

The right-hand side captures thermodynamic relaxation at a rate Γ_ϕ, driven by the gradient of chemical potential $\mu \equiv \delta F_\phi / \delta \phi$ associated with the classic Ginzburg-Landau free energy,

$$F_\phi = \int_{\mathbf{r}} \left[\frac{a_\phi}{4} \phi^2 \left(1 - \phi\right)^2 + \frac{k_\phi}{2} |\nabla \phi|^2 \right] d\mathbf{r}. \tag{5}$$

Here, a_ϕ represents the characteristic energy associated with phase separation and k_ϕ penalizes composition gradients. Together, they set the characteristic relaxation time $\tau_\phi = k_\phi / \Gamma_\phi a_\phi^2$, the interface width $\ell_\phi = \sqrt{k_\phi / a_\phi}$, and interfacial energy density $\epsilon_\phi = \sqrt{a_\phi k_\phi}$.

Fluid flow is modeled using the Toner–Tu–Swift–Hohenberg (TTSH) equation [5–8]

$$\partial_t \mathbf{v} + \lambda_0 \, \mathbf{v} \cdot \nabla \mathbf{v} = -\nabla p^* - \alpha \left(\phi - \frac{|\mathbf{v}|^2}{v_0^2} \right) \mathbf{v} + \nu \nabla^2 \mathbf{v} - \zeta \nabla^4 \mathbf{v} + \mu \nabla \phi, \tag{6}$$

in addition to the condition of incompressibility $\nabla \cdot \mathbf{v} = 0$.

To distinguish between active and passive flows, we introduce ϕ-dependent coefficients into the TTSH equations. Specifically, the advection coefficient is taken as $\lambda_0 = 1 - S_v \phi$, so that $\lambda_0 = 1$ in the passive phase and $\lambda_0 = 1 - S_v$ in the bacterial phase. This term captures the self-advection associated with bacterial motility. Here S_v characterizes the

hydrodynamic nature of the swimmers and is positive for pusher-like bacteria, as in our experiments. The modified pressure is written as $p^* = p - \phi S_v d^{-1}\mathbf{v}^2$, where d is the spatial dimension. This expression includes an isotropic active pressure contribution in the bacterial phase.

The parameters α and v_0 jointly control the local speed saturation: the passive phase is driven toward $v \simeq 0$, whereas the active phase is driven toward a characteristic collective speed $v \simeq v_0$. The effective viscosity coefficient is defined as $\nu = \nu_1(1-\phi) + \nu_2\phi$, where $\nu_1 > 0$ is the viscosity-like damping coefficient in the passive phase, while $\nu_2 < 0$ is the active viscosity-like coefficient in the bacterial phase. The negative sign of ν_2 represents coarse-grained active energy injection by bacterial motility and destabilizes long-wavelength velocity fluctuations. This instability is regularized by the higher-order viscosity coefficient $\zeta > 0$, which suppresses short-wavelength modes. Together, ν_2 and ζ determine the intrinsic active-flow length scale, $\ell_\mathrm{v} \sim \sqrt{\zeta/|\nu_2|}$. Consistently, in both 2D and 3D simulations, the velocity correlation length extracted from the simulated flow increases approximately linearly with $\sqrt{\zeta/|\nu_2|}$, as shown in Supplementary Figure 5. In addition to setting the flow length scale, ν_2 and ζ also define the characteristic active-flow velocity scale $\sqrt{-\nu_2^3/\zeta}$. In the simulations, v_0 is chosen to be of the same order as the intrinsic active-flow velocity scale $\sqrt{-\nu_2^3/\zeta}$, so that the nonlinear speed saturation and the active-flow instability are controlled by comparable velocity scales. Therefore, the choice of ν_2 is coupled to both the intrinsic active-flow length scale and the characteristic collective-flow speed scale of the model. The last term, $\mu\nabla\phi$, represents the capillary force density associated with interfacial tension [9–11].

To make the model dimensionless, we rescale the length by the characteristic interface width $\ell_\phi = \sqrt{k_\phi/a_\phi} = 1$ in both 3D and 2D simulations, time by the typical relaxation time $\tau_\phi = \Gamma_\phi a_\phi^2/k_\phi = 1$ in both 3D and 2D simulations, and energy by interfacial energy per unit length $\epsilon_\phi = \sqrt{a_\phi k_\phi}$ (1 in 3D and 50 in 2D simulations), which gives the interfacial tension $\gamma_0 = \sqrt{2a_\phi k_\phi}/3$ [12]. Unless otherwise specified, all results from the simulation will be presented in the dimensionless units.

B. Numerical implementation

We solve Eqs. (4) and (6) using the finite-difference method. The simulation box is discretized into a uniform grid with spacing $h = 1$, and spatial derivatives are evaluated

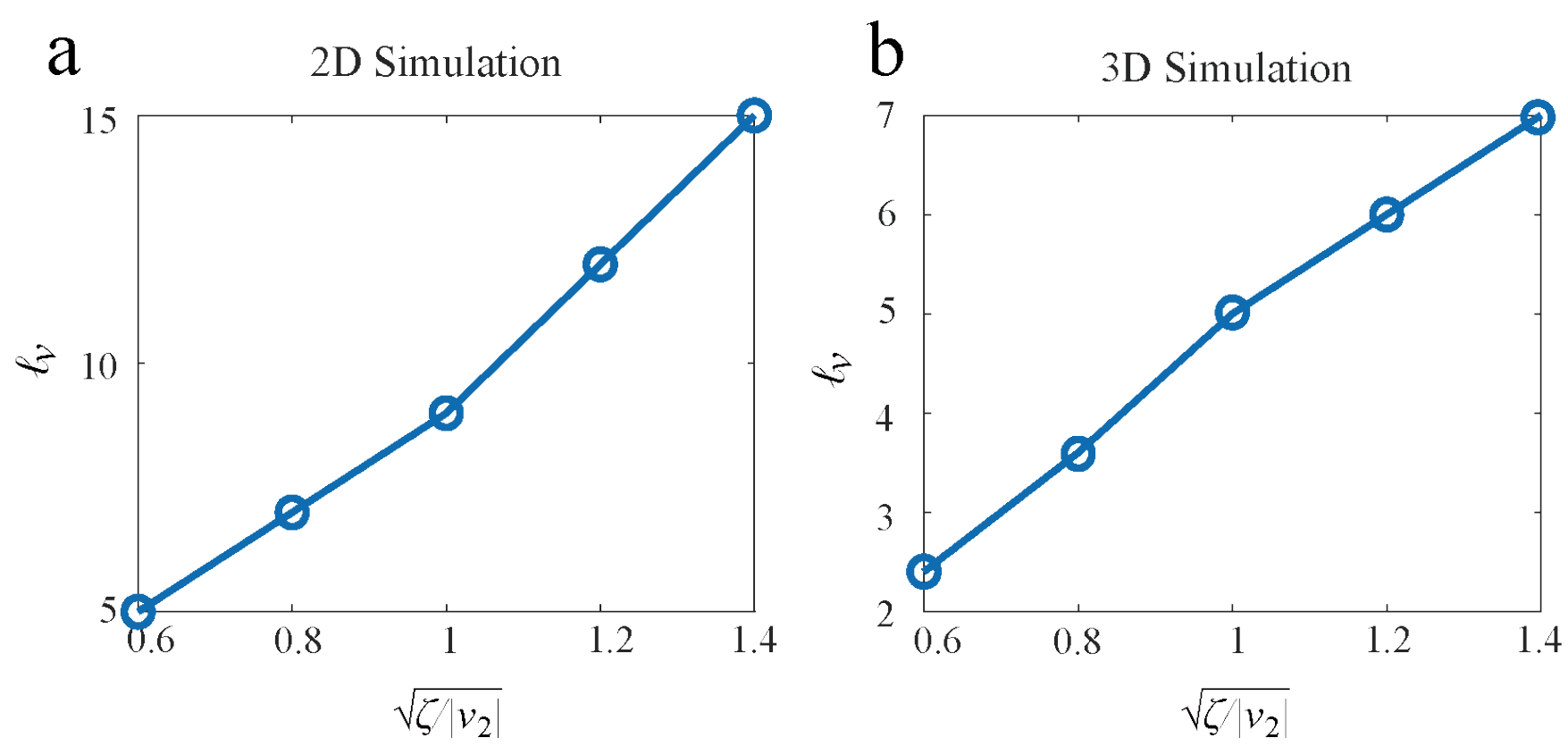

Supplementary Figure 5. Relation between the correlation length of the flow field ℓ_{v} and simulation parameter $\sqrt{\zeta/|\nu_2|}$ in both (a) 2D and (b) 3D system. The default values of ℓ_{v} used in 2D and 3D simulations both are related to $\sqrt{\zeta/|\nu_2|} = 1$.

using a second-order isotropic central-difference stencil. Time integration is performed using a forward Euler scheme with a timestep $\Delta t = 0.002$. Incompressibility of the velocity field is enforced through a projection method (see Ref. [13] for the CUDA implementation).

Simulations of the 3D bacterial droplet are performed in a cubic box whose size is $L \times L \times L$ with periodic boundary conditions. Simulations are initiated with isotropic randomly chosen field $\mathbf{v}(\boldsymbol{x}, 0)$ and $\phi(\boldsymbol{x}, 0) = (\tanh(r_0 - \Delta r) + 1)/2$, which gives a spherical bacterial droplet with radius $r_0 = L/2$ and an interface width 1, where $\Delta r = |\boldsymbol{x} - \boldsymbol{x}_c|$ and $\boldsymbol{x}_c$ is the center of the box. In the 2D simulations, we use square periodic box of size $L \times L$. Simulations are initiated with isotropic randomly chosen field $\mathbf{v}(\boldsymbol{x}, 0)$ and $\phi(\boldsymbol{x}, 0) = (1 - \tanh(2(x - 3L/4)))/2 + (1 + \tanh(2(x - L/4)))/2 - 1$, which gives 2 flat interfaces at position $x = L/4$ and $x = 3L/4$ with interface width 1.

C. Parameter selection

All default parameters used in the simulations are listed in Supplementary Table 1. The simulations are performed in nondimensional units. Following the nondimensionalization strategy of Ref. [5], the simulation units are related to experiments by matching two experimentally measurable quantities: the velocity-correlation length and the collective-flow speed.

In the model, the active-flow length is controlled by $\sqrt{\zeta/|\nu_2|}$. The measured velocity-

correlation length ℓ_v scales with this intrinsic length, $\ell_v \sim \sqrt{\zeta/|\nu_2|}$ (Supplementary Figure 5). In the default simulations, $\sqrt{\zeta/|\nu_2|} = 1$ gives $\ell_v \simeq 5$ in simulation units. The simulation length scale is therefore calibrated by matching ℓ_v to the experimental value. Because the experimentally measured ℓ_v varies with droplet size (See Supplementary Figure 17), the simulation-to-physical length conversion is determined separately for each droplet by matching its measured ℓ_v, rather than by using a single global conversion factor.

For the droplet in Fig. 2a, $\ell_v \simeq 20$ μm, corresponding to 1 simulation unit $\simeq 4$ μm. For the droplet in Fig. 2b, $\ell_v \simeq 50$ μm, corresponding to 1 simulation unit $\simeq 10$ μm. When we vary the active-flow correlation length in the simulations, we change $\sqrt{\zeta/|\nu_2|}$ accordingly.

The magnitude of the active flow is controlled by the v_0. For the default simulations, we choose the parameters $v_0 = 0.1$ in simulation units. This velocity scale corresponds to an experimental collective-flow speed of approximately 3-4 μm/s.

It should be noted that the activity strength in the simulations is subject to a practical lower limit (approximately $\nu_2 > -0.5$), which corresponds to the range of activity that yields stable macroscopic stretching of droplets in experiments. Beyond this threshold, the active droplets in simulations undergo "melting" due to hydrodynamic instabilities, losing their cohesive structure. This melting occurs because high activity drives strong interfacial deformation, creating regions of high curvature. In simulations, the fixed interfacial thickness cannot resolve these excessive curvatures, leading to the eventual dissolution of the droplet. In contrast, higher-activity morphologies can still be observed in the experimental system. Therefore, it is difficult to reproduce the multi-protrusion droplets and thin filaments observed in experiments in Sec. S5.

Section S3. QUANTIFICATION OF DROPLET DYNAMICS

This section outlines the quantitative procedures used to analyze droplet shape fluctuations, 3D morphology, contact-line dynamics, and coarsening. It includes interface extraction, fluctuation-spectrum calculations, spherical harmonic decomposition, and dynamic structure factor measurements in both experiment and simulation.

Parameter	Rescaled value (3D)	Rescaled value (2D)
α	-0.005	-0.01
v_0	0.1	0.15
ν_1	1	0.5
ν_2	-0.1	-0.2
ζ	0.1	0.2
S_v	2.5	1.7
a_ϕ	1	50
k_ϕ	1	50
Γ_ϕ	1	1

Supplementary Table 1. Summary of key parameters and their typical values in 3D and 2D simulations. Unless otherwise specified, the parameters in the table are the default simulation parameters.

A. Fluctuation spectra

1. Extraction of droplet interface and contour

To compute fluctuation spectra, we first extract the contour line of the droplet at the equator. In experiments, the contour is extracted using the MATLAB function 'edge', which identifies pixels with the maximum intensity gradient. In simulation, we first binarize the phase field ϕ. The contour is defined by the isocontour $\phi = 0.5$. After obtaining the contour, we compute the center of mass (x_c, y_c) of the equatorial cross-section and locate all boundary pixels (x_i, y_i). These positions are then transformed into polar coordinates (r_i, φ_i), with the origin at (x_c, y_c) and the polar axis aligned along the x-direction. Because the simulated interface consists of a finite number of discrete grid points, the resulting (r_i, φ_i) are interpolated to reconstruct a continuous radial profile $r(\varphi)$ (Supplementary Figure 6).

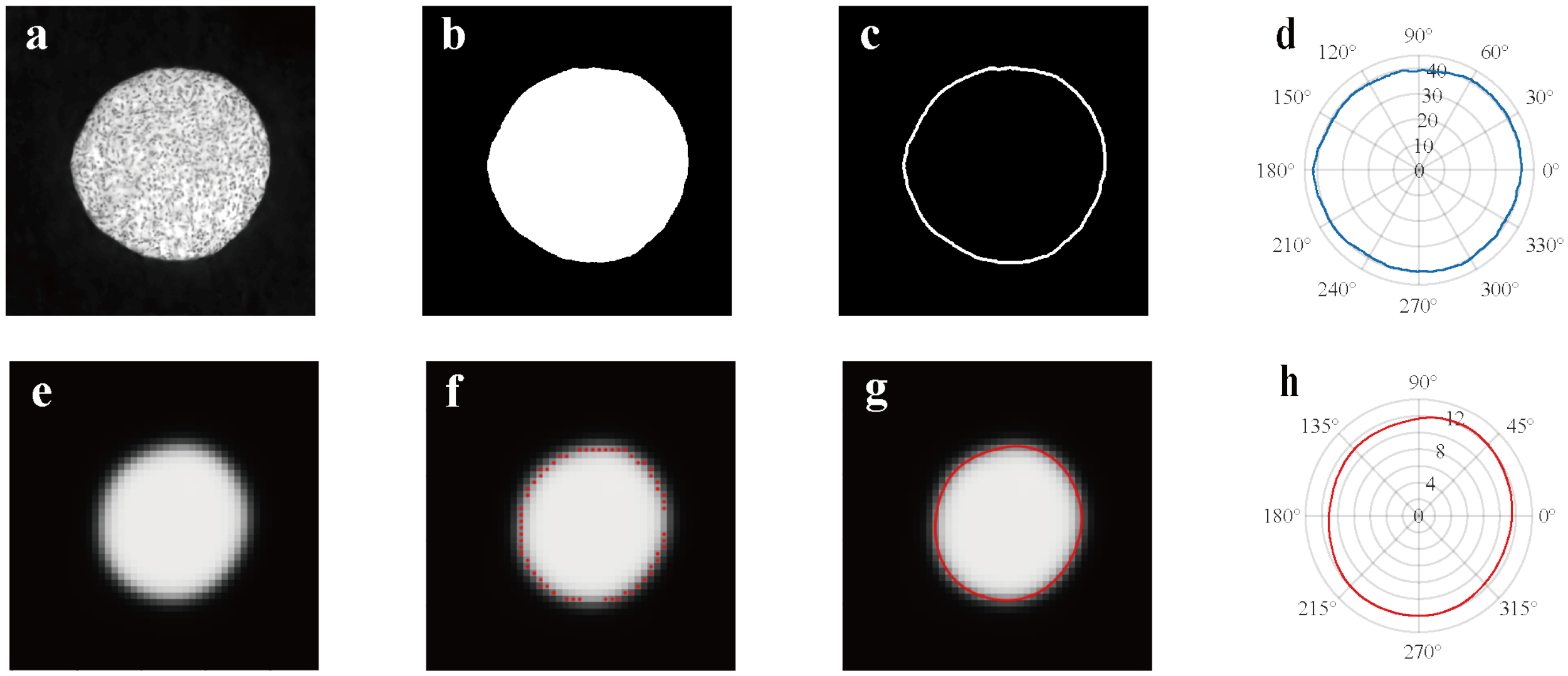

Supplementary Figure 6. Contour analysis of a droplet cross-section. (a–d) Experimental contour extraction of a droplet. (a) Cross-section of a droplet. (b) Binarized image of (a). (c) Extracted contour from (b). (d) Radial distance function $r(\varphi)$ calculated from the contour in (c). (e–h) Contour analysis in simulation. (e) Equatorial phase field. (f) Red dots indicate the grid points where $\phi = 0.5$. (g) Interpolated contour from (f). (h) Radial distance function $r(\varphi)$ obtained from the contour in (g).

2. *Calculation of static fluctuation spectra*

The static fluctuation spectrum is defined as

$$S_k = \langle |\hat{u}_k|^2 \rangle_t, \tag{7}$$

where

$$\hat{u}_k(t) = \frac{1}{2\pi R} \int_0^{2\pi} r(\varphi, t)\, e^{-ik\varphi}\, d\varphi \tag{8}$$

is the Fourier component of the equatorial contour at azimuthal mode number n_k, corresponding to wave number $k = n_k/R$. In experiments, $\hat{u}_k(t)$ is computed using a non-uniform discrete Fourier transform following [14], which accounts for non-uniform azimuthal sampling.

In the experiments, we conduct nine independent experiments under the low-bacterial-density condition and analyze approximately thirty droplets with different radii. We select four representative droplets of different sizes for display in the main text. In Fig. 2c, each curve corresponds to the fluctuation spectrum obtained from one individual droplet. For

each droplet, one long experimental acquisition is divided into 30 statistically independent segments. The fluctuation spectrum is first calculated separately for each segment, and the mean spectrum is then obtained by averaging over these 30 segment-resolved spectra. The standard deviation among the 30 spectra is used to estimate the segment-to-segment fluctuation at each wave number.

Because the data are plotted on logarithmic axes, we report symmetric error bars in logarithmic space. Specifically, for a measured quantity $S(k)$, the logarithmic error is estimated as $\frac{1}{\ln 10}\frac{\sigma(S)}{\mu(S)} \simeq 0.434\frac{\sigma(S)}{\mu(S)}$, where $\mu(S)$ and $\sigma(S)$ denote the mean and standard deviation of S over the 30 independent segments, respectively. The error bars shown in Fig. 2c therefore correspond to

$$\log_{10} S \pm 0.434\frac{\sigma(S)}{\mu(S)}.$$

This procedure follows the method described in Ref. [15] and allows the uncertainty to be displayed symmetrically on a logarithmic scale. The same procedure is applied in simulations. However, the diffuse interface spans multiple grid points, resulting in a smoother contour and deviations in the high-k regime compared to experiments.

Since $n_k = 0$ corresponds to the mean radius R, and $n_k = 1$ represents center-of-mass motion, the lowest nontrivial mode is $n_k = 2$, Hence, we present $S(k)$ starting from $n_k = 2$. At equilibrium, interfacial fluctuations obey the equipartition theorem. For a liquid-liquid interface governed by surface tension, this gives $S(k) \sim k^{-2}$, whereas vesicle membranes can exhibit a crossover from $S(k) \sim k^{-1}$ at small k to $S(k) \sim k^{-3}$ at large k, reflecting the crossover from tension-dominated to bending-dominated membrane fluctuations [14, 16]. In active systems, continuous energy injection breaks this equilibrium balance and can produce steeper fluctuation spectra, with reported power-law exponents between k^{-3} and k^{-4} in active droplets and vesicles [15, 17–20].

Most previous studies focused on active vesicles [15, 17, 19, 20], where membrane tension, bending elasticity, and thermal membrane fluctuations all contribute to the observed spectra. By contrast, a liquid-liquid droplet interface has no membrane bending. The interfacial tension of liquid-liquid phase-separated systems is typically 0.1-10 μN/m, it is large enough that equilibrium thermal capillary fluctuations are far below the micrometer-scale deformations resolved in our experiments. Thus, in liquid-liquid phase separating systems, the measured spectra mainly reflect activity-induced interfacial dynamics rather than a preexisting thermal fluctuation background.

Another important distinction concerns the nature and length scale of the active forcing. In several previous active-vesicle systems, the encapsulated active components do not exhibit collective motion [15, 17, 20]. Dense bacterial suspensions confined in vesicles [19] and collective Quincke rollers inside quasi-two-dimensional droplets [18] provide closer analogues to our system because the internal active medium can generate collective flows. However, in these cases the system size appears to be comparable to the correlation length of the internal active flow, which may limit the scale separation needed to resolve a clear crossover associated with the collective-flow length scale. In addition, in quasi-two-dimensional Quincke-roller droplets, contact-line motion might be also considered as one possible dissipation channel for interfacial relaxation, although the observed dynamics also reflect the activity-driven flows generated by the rollers. Our bacteria-filled droplets provide a geometry in which the droplet size and the internal velocity correlation length can be separated, allowing this crossover to be probed more directly.

Active deformation of a flat liquid-liquid interface provides a useful comparison. When collective microtubule motion drives a flat interface, the fluctuation spectrum exhibits a pronounced feature at the energy-injection scale [21]. In that work, the spectrum is reported for the local tangent angle, $S_\theta(k) = \langle|\theta_k|^2\rangle$, with $S_\theta(k) \sim k^2$ at small k and $S_\theta(k) \sim k^{-3}$ at large k. When expressed in terms of the interfacial deformation field, the corresponding scaling becomes k^1 at small k and k^{-6} at large k. For consistency with other studies, in Supplementary Table 2 we adopt the definition based on the Fourier transform of the interfacial height field.

Ref	Active system	Collective motion	Scaling of $S(k)$
[17]	Vesicle + Janus particles	No	small k: k^{-4}; large k: k^{-3}
[18]	2D Droplet + Quincke rollers	Yes	k^{-4}
[15]	Vesicle + bacteria	No	k^{-3}
[19]	Vesicle + bacteria	Yes	small k: k^{-4}; large k: k^{-1}
[20]	Vesicle + microtubules	No	k^{-3}
[21]	2D liquid–liquid interface + microtubules	Yes	small k: k^1; large k: k^{-6}

Supplementary Table 2. Reported scaling exponents of interfacial fluctuation spectra in various active systems.

3. Spherical harmonic decomposition of 3D droplet shape

Simulations provide full three-dimensional information of the droplet interface, enabling a spherical harmonic decomposition of the droplet shape.

$$r\left(\theta,\varphi,t\right)=R\left(1+\sum_{\ell=0}^{\ell_{max}}\sum_{m=-\ell}^{\ell}f_{\ell m}\left(t\right)Y_{\ell m}\left(\theta,\varphi\right)\right). \tag{9}$$

The spherical harmonics $Y_{\ell m}$ are defined by:

$$Y_{\ell m}=n_{\ell,m}P_{\ell}^{m}(\cos\theta)e^{im\varphi}, n_{\ell,m}=\sqrt{\frac{(2\ell+1)(\ell-m)!}{4\pi(\ell+m)!}}. \tag{10}$$

where θ and φ are the polar and azimuthal angles, and P_{ℓ}^{m} are the associated Legendre functions of degree ℓ and order m. In terms of the spherical harmonic mode amplitudes, we calculate the fluctuation spectrum for each mode $A_{\ell}=\langle|f_{\ell m}|^2\rangle_m$ for droplets with different sizes (Supplementary Figure 7a). We further calculate the energy of each ℓ [22], which is given by:

$$E_{\ell}=\langle E_{\ell,m}\rangle_m=\frac{1}{2}\left(\ell+2\right)\left(\ell-1\right)R^2\gamma_0A_{\ell}. \tag{11}$$

Energy for droplets of different sizes collapse onto a single curve (Supplementary Figure 7b). At small ℓ, the energy grows slowly, reaches a maximum around $\ell\sim\ell_{\mathrm{v}}^{-1}$, and decays at higher ℓ. This peak identifies the characteristic scale at which active turbulence injects energy into the interface.

To connect the 3D decomposition to the equatorial contour, we express the contour as:

$$r\left(\varphi,t\right)=R\sum_{n_k=-\ell_{max}}^{\ell_{max}}\hat{u}_k\left(t\right)e^{in_k\varphi}=r\left(\pi/2,\varphi,t\right). \tag{12}$$

Where n_k is the mode number with $k=n_k/R$. The Fourier coefficient for the n_k-th mode is then given by,

$$\hat{u}_k\left(t\right)=\frac{1}{2\pi R}\int_0^{2\pi}r\left(\varphi,t\right)e^{-in_k\varphi}d\varphi=\sum_{\ell=n_k}^{\ell_{max}}f_{\ell n_k}\left(t\right)n_{\ell n_k}P_{\ell n_k}\left(0\right). \tag{13}$$

Then the mean squared amplitude of $\hat{u}_k$ projected by spherical harmonic modes is given by:

$$\langle |\hat{u}_k|^2 \rangle = \sum_{\ell=n_k}^{\ell_{max}} \langle |f_{\ell n_k}|^2 \rangle n_{\ell n_k}^2 |P_{\ell n_k}(0)|^2. \tag{14}$$

Eq. (14) represents the 2D fluctuation spectrum projected from the 3D fluctuation spectrum. Because the summation range narrows as k increases, $\langle |\hat{u}_k|^2 \rangle$ decreases monotonically with k. Thus the projected 2D spectrum does not exhibit a sharp peak but instead shows a transition in slope. The transition point corresponds to the peak of the 3D energy spectrum at $k \simeq 1/\ell_\mathrm{v}$ (Supplementary Figure 7c).

To confirm the robustness of this mechanism, we vary the flow correlation length ℓ_v in simulations. As shown in Supplementary Figure 8, the scaling transition consistently occurs at $k \simeq 1/\ell_\mathrm{v}$. For comparison, we also compute the fluctuation spectra of a flat 2D interface with height profile

$$h(x) = \frac{1}{\sqrt{L}} \sum_k \hat{h}_k e^{ikx}, \tag{15}$$

whose energy spectrum

$$E_k = \frac{1}{2} \gamma_0 k^2 \langle |\hat{h}_k|^2 \rangle \tag{16}$$

likewise exhibits a transition at $k = 1/\ell_\mathrm{v}$ (Fig. 3c).

4. *Calculation of dynamic structure factor*

To obtain the dynamical structure factor, we first construct kymographs of the deformation field $r(\varphi, t)$. At each time t, the boundary points (r_i, φ_i) are interpolated onto a uniform grid in the azimuthal angle with spacing $\Delta\varphi = \pi/100$ over the range $[0, 2\pi)$. The resulting kymographs display inclined stripe patterns characteristic of propagating modes (Supplementary Figure 9a and Supplementary Figure 10a).

The dynamic structure factor (DSF), $S(k, \omega)$, is computed as the squared modulus of the spatiotemporal Fourier transform of the interfacial fluctuation $r(\varphi, t)$. Specifically, we first calculate $\tilde{u}_{k,\omega} = \mathcal{F}\{r(\varphi, t)\}$ using a two-dimensional fast Fourier transform (`fft2`) in MATLAB, and then define $S(k, \omega) = |\tilde{u}_{k,\omega}|^2$. Prior to the transform, a Kaiser window is applied to each time segment of duration ΔT. A sliding-window procedure is employed: for each start time t_0, $r(\varphi, t)$ is windowed over the interval $[t_0,\, t_0 + \Delta T]$, transformed, and the corresponding $S_{t_0}(k, \omega)$ is computed. The final DSF is obtained by averaging over all overlapping windows.

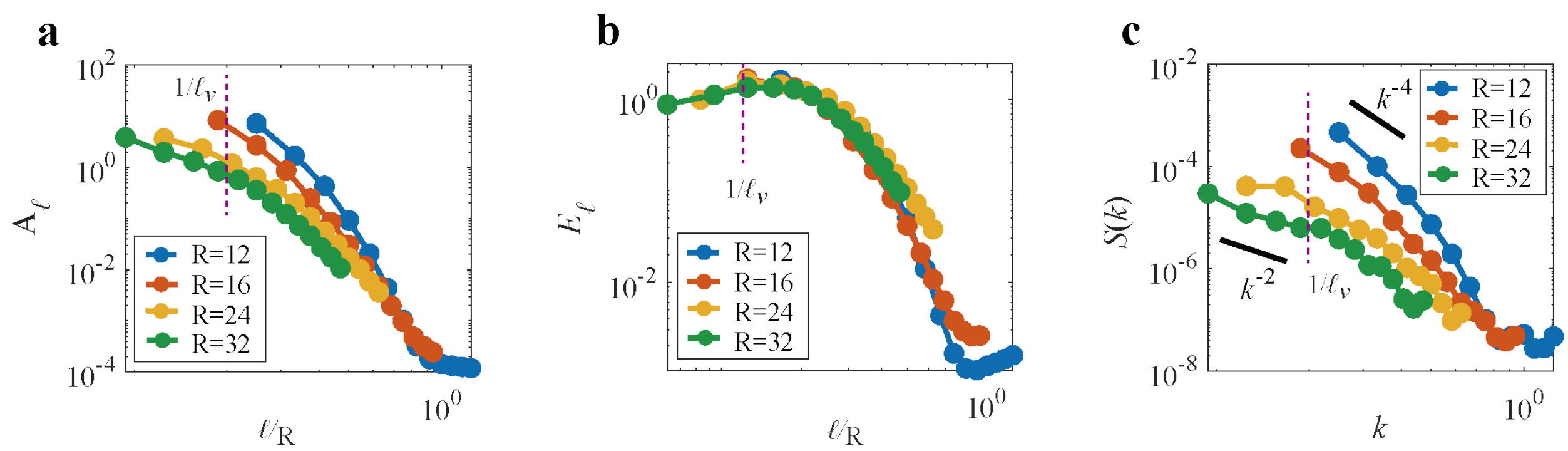

Supplementary Figure 7. Contour analysis using the spherical harmonic method. (a) Normalized spherical harmonic components for each ℓ, defined as $A_\ell = \langle |f_{\ell,m}|^2 \rangle_m$. (b) Energy $E_\ell = \langle E_{\ell,m} \rangle_m$. (c) Fluctuation spectrum $S(k) = \langle |\hat{u}_k|^2 \rangle$ as a function of k. The curves in (c) are calculated from 3D information through Eq. (14). This projection from 3D to 2D introduces minor computational deviations, resulting in quantitative differences between the curves in (c) and those obtained directly from the 2D cross-section (Fig. 2 g). Nevertheless, the overall spectral shape remains consistent. The dashed line indicates $1/\ell_{\mathrm{v}}$, where ℓ_{v} is the correlation length of the flow field. Simulation parameters are listed in Supplementary Table 1.

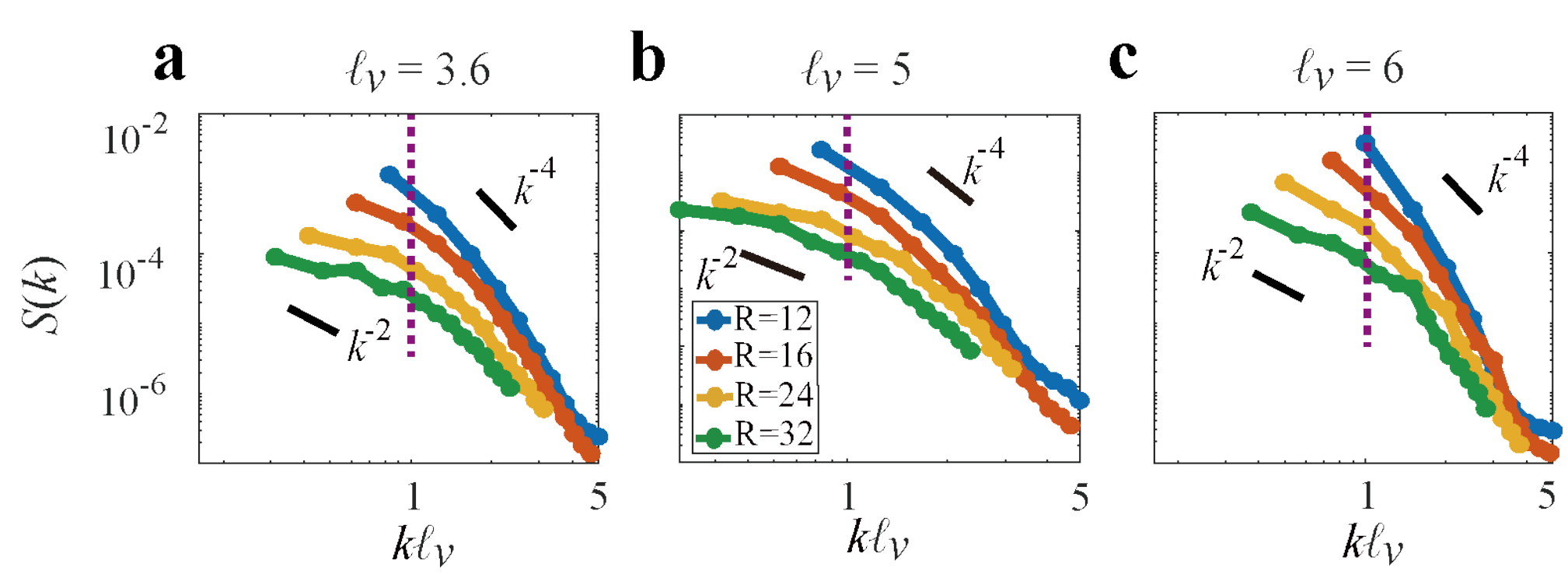

Supplementary Figure 8. Scaling transition of fluctuation spectra with different correlation length ℓ_{v}. (a) $\ell_{\mathrm{v}} = 3.6$, (b) $\ell_{\mathrm{v}} = 5$ and (c) $\ell_{\mathrm{v}} = 6$. The horizontal axis is normalized by the corresponding flow correlation lengths ℓ_{v}; the dashed line marks $k\ell_{\mathrm{v}} = 1$. In the simulation, ν_2 is fixed at -0.1, and $\ell_{\mathrm{v}} = 3.6, 5, 6$ are obtained by setting $\zeta = 0.064, 0.1, 0.144$ respectively. The remaining parameters are set to their default values.

Because the contour-fluctuation amplitude decays with increasing k, the DSF is dominated by the lowest spatial modes (Supplementary Figure 9b and Supplementary Fig-

ure 10b). To better resolve the dispersion relation, each spatial mode is normalized by its maximum value:

$$S_n(k_i, \omega) = \frac{S(k_i, \omega)}{\max_\omega S(k_i, \omega)}. \tag{17}$$

The normalized DSFs for both experiments and simulations are shown in Fig. 2(d,h) of the main text and in Supplementary Figure 9c and Supplementary Figure 10c.

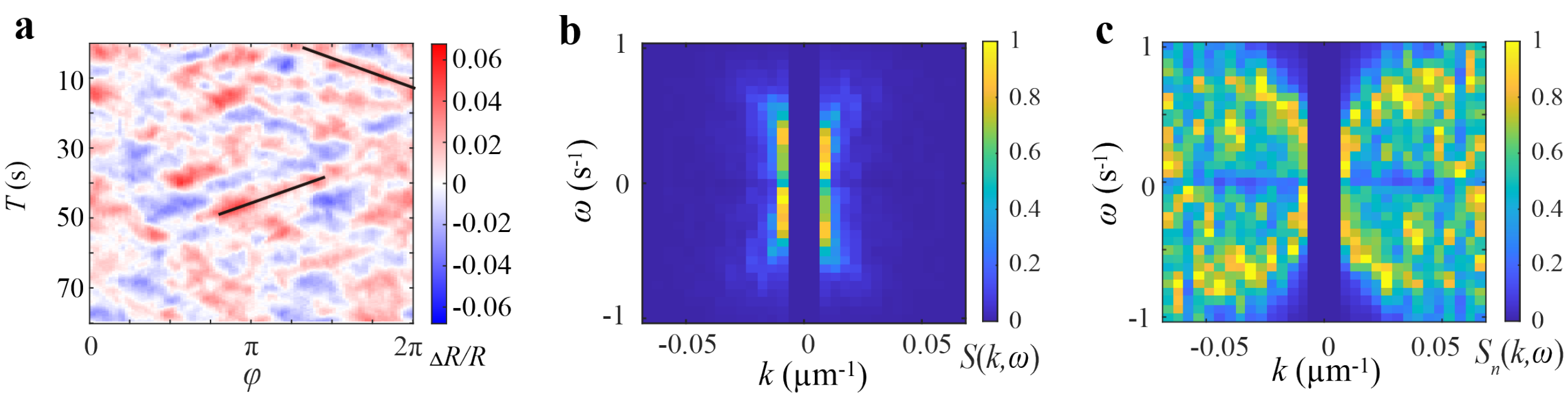

Supplementary Figure 9. (a) Kymographs of a droplet with $R = 226$ μm. The colorbar represents $\Delta R/R$, where $\Delta R = r(\varphi_i, t) - R$ denotes the deviation from the mean radius. (b) The DSF $S(k, \omega)$ of the droplet in (a). (c) The normalized DSF $S_n(k, \omega)$ of (b) at each k.

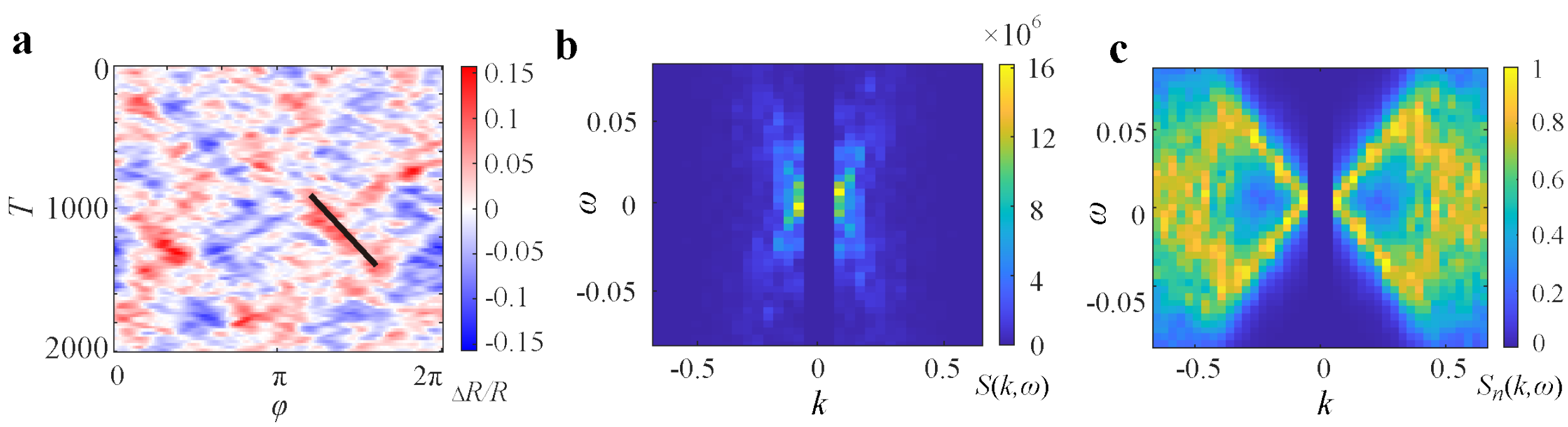

Supplementary Figure 10. (a) Kymographs of a droplet with $R = 32$. The colorbar represents $\Delta R/R$, where $\Delta R = r(\varphi_i, t) - R$ denotes the deviation from the mean radius. (b) The DSF $S(k, \omega)$ of the droplet in (a). (c) The normalized DSF $S_n(k, \omega)$ of (b) at each k. Simulation parameters are listed in Supplementary Table 1.

B. 3D morphological analysis

To quantify the 3D morphology of high-activity droplets, we measure their surface areas and aspect ratios. The experimental 3D confocal image stacks of active droplets are first

binarized to extract droplet contours. The resulting binary masks are smoothed with a Gaussian filter to reduce voxel-level pixelation and enhance surface continuity.

Morphological properties—including surface area, volume, and aspect ratio—are calculated using MATLAB's `regionprops3` function. The aspect ratio is defined as the ratio between the major and minor axes of the best-fit ellipsoid obtained from the normalized second central moments.

The surface area S is normalized by the surface area of a sphere with the same volume, $S_0 = 4\pi R_{\mathbf{eff}}^2$, where $R_{\mathbf{eff}} = \left(\frac{3V}{4\pi}\right)^{1/3}$ is the radius of a sphere with volume V. We define the relative area as $\Delta \equiv \frac{S}{S_0} - 1$.

C. Quantification of droplet–substrate interaction

The contact angle of passive droplets is measured from side-view images, yielding a value of 150° (Supplementary Figure 11a). Low-bacterial-density droplets display similar contact angles (Supplementary Figure 11b), whereas high-bacterial-density droplets exhibit strong interfacial fluctuations that make a static contact-angle definition impossible.

The contact line is extracted from the first bright slice in the confocal z-stack, i.e., the lowest optical plane in which the droplet appears. This slice is binarized to obtain the footprint contour. For low-bacterial-density droplets, activity leads to deformation of contact line (Supplementary Figure 12). In contrast, high-bacterial-density droplets display complex and dynamic contact lines, indicating substantial deformation and altered substrate interactions [Supplementary Figure 11c, Supplementary Figure 13].

D. Quantification of coarsening dynamics

1. Definition of bulk-droplet volume fraction

We define Φ_B as the volume fraction of the droplets in the bulk. A droplet is classified as "in the bulk" if the vertical distance between its center of mass and the substrate exceeds its effective radius $R_{\mathbf{eff}} = \left(\frac{3V}{4\pi}\right)^{1/3}$.

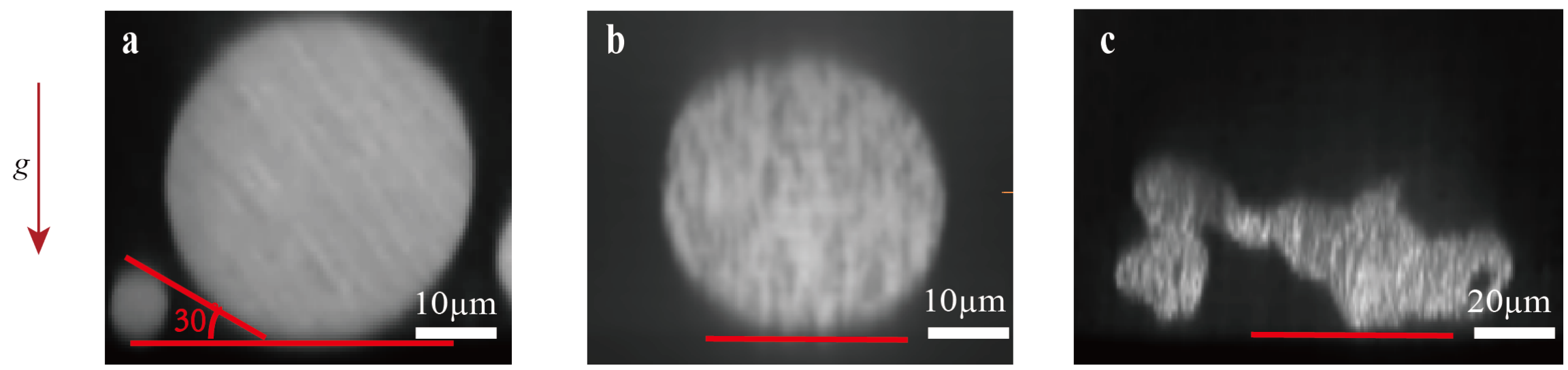

Supplementary Figure 11. Relationship between the droplet and the substrate. (a) The side view of a passive droplet. The contact angle is about 150°. (b) The side view of the droplet with low bacteria concentration in Fig. 4a-c. (c) The side view of the droplet with high bacterial concentration in Fig. 4d-f. The horizontal red line indicates the substrate position.

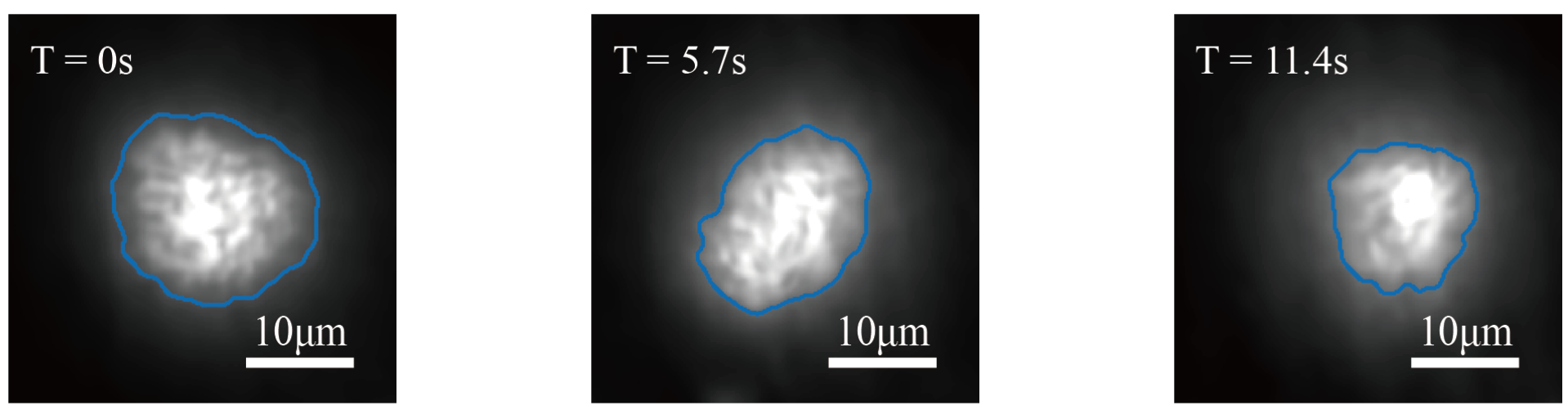

Supplementary Figure 12. Temporal evolution of the contact line in a droplet with low bacteria concentration. The blue line marks the contact line.

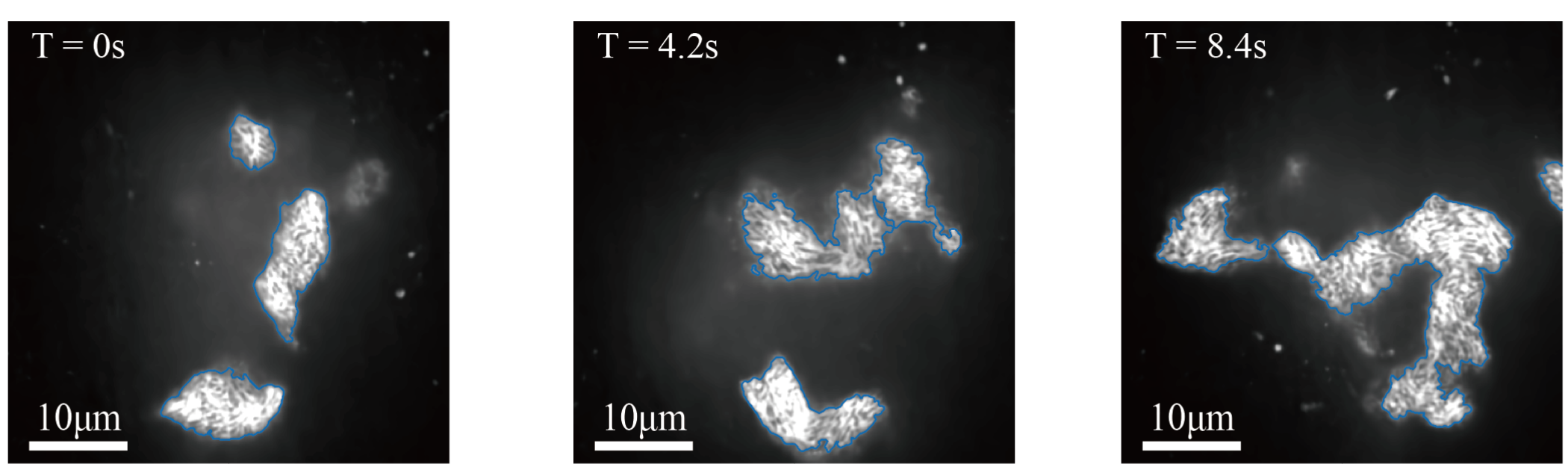

Supplementary Figure 13. Temporal evolution of the contact line in a droplet with high bacteria concentration. The blue line marks the contact line.

2. *Measurement of correlation length during coarsening*

To quantify domain growth during coarsening, we extract a three-dimensional phase field from the confocal stacks. The image stack is cropped to a 40 μm-thick region above the substrate, where droplets are well resolved. The dextran-rich phase is assigned $\phi = 1$ and the PEG-rich phase $\phi = 0$ by binarizing the fluorescence intensity. To quantify the spatial correlation, we compute the isotropic two-point correlation function and extract the characteristic correlation length. We first subtract its mean $\langle\phi\rangle$ to obtain zero-mean fluctuations $\delta\phi = \phi - \langle\phi\rangle$. The normalized autocorrelation function $C(\mathbf{r})$ is then computed via the Wiener-Khinchin theorem using fast Fourier transforms (FFT):

$$C(\mathbf{r}) = \frac{1}{C_0}\mathcal{F}^{-1}\left[|\mathcal{F}[\delta\phi]|^2\right], \tag{18}$$

where $\mathcal{F}$ and $\mathcal{F}^{-1}$ denote the 3D forward and inverse Fourier transforms, respectively, and $C_0 = \max(C)$ ensures normalization such that $C(\mathbf{0}) = 1$.

To obtain the radial (isotropic) correlation function $C(r)$, the 3D correlation values are averaged over spherical shells of radius r. Specifically, voxels with distances $r - \Delta r/2 < \|\mathbf{r}\| < r + \Delta r/2$ are grouped and averaged to yield $C(r)$. The resulting $C(r)$ is then fitted with an exponential decay function of the form:

$$C(r) \sim \exp\left(-\frac{r}{\xi}\right), \tag{19}$$

where ξ is the correlation length. The fitting is performed using a nonlinear least-squares routine, and ξ is taken as the characteristic length scale of spatial correlations in the system.

Phase separation initiates as soon as the PEG–dextran–bacteria mixture is prepared, and sedimentation-driven coarsening proceeds concurrently. Because mounting the sample and starting the confocal acquisition require approximately one minute, the droplets continue to coarsen during this interval. Consequently, the first recorded frame (at $T \sim 1$ min) already displays a substantially coarsened droplet distribution.

3. *Characterizing the role of bacterial concentration on coarsening dynamics*

Immediately after preparation, phase separation and sedimentation begin simultaneously. Coarsening proceeds predominantly through droplet-droplet coalescence rather than Ostwald

ripening. The effect of bacteria can be separated into a passive-loading contribution and a motility-driven active contribution.

Even as non-motile inclusions, bacteria can influence sedimentation and coarsening. Because the bacterial density is slightly higher than that of the dextran-rich phase, adding bacteria can enhance the sedimentation of dextran-rich droplets. To quantify this passive contribution, we use the non-motile bacterial strain RP1616 as a control and add it to the system at the same bacterial volume fraction. As shown in Supplementary Figure 14, non-motile bacteria accelerate the decrease of the bulk volume fraction, indicating that bacteria can promote sedimentation to some extent. They also speed up the apparent onset of phase separation, and the final droplet-size distribution is shifted toward larger droplets compared with the bacteria-free control, as shown in Supplementary Figure 15 and Supplementary Figure 16. These results show that passive bacterial loading alone can facilitate sedimentation and coalescence. However, motile bacteria induce substantially faster sedimentation and coarsening, especially at high bacterial density, demonstrating an additional activity-dependent contribution.

Motility-driven active flows further accelerate coarsening in several ways. First, they increase droplet mobility and thereby enhance the encounter rate between neighboring droplets. Second, activity-induced deformations enlarge the effective interaction cross-section, increasing the probability of physical contact and coalescence. Third, active flows stir the surrounding fluid and facilitate the transport, advection, and mixing of smaller droplets. A feedback mechanism can further amplify this effect: larger droplets sediment faster [23], which promotes additional coalescence with droplets below and accelerates the overall coarsening process.

Upon contacting the substrate, inactive droplets remain stationary and only grow by merging with droplets falling from above. Weakly active droplets perform local centroid motion, intermittently exploring the substrate and enhancing their likelihood of encountering neighboring droplets (Fig. 5c). At high bacterial density, droplets undergo strong shape fluctuations and active random displacements (Fig. 5f), sustaining coalescence events throughout the imaging period (Fig. 5g). Consequently, highly-bacterial-density samples reach a strongly coarsened state in which the majority of the mass resides within only a few large droplets (Figs. 1d and 5h).

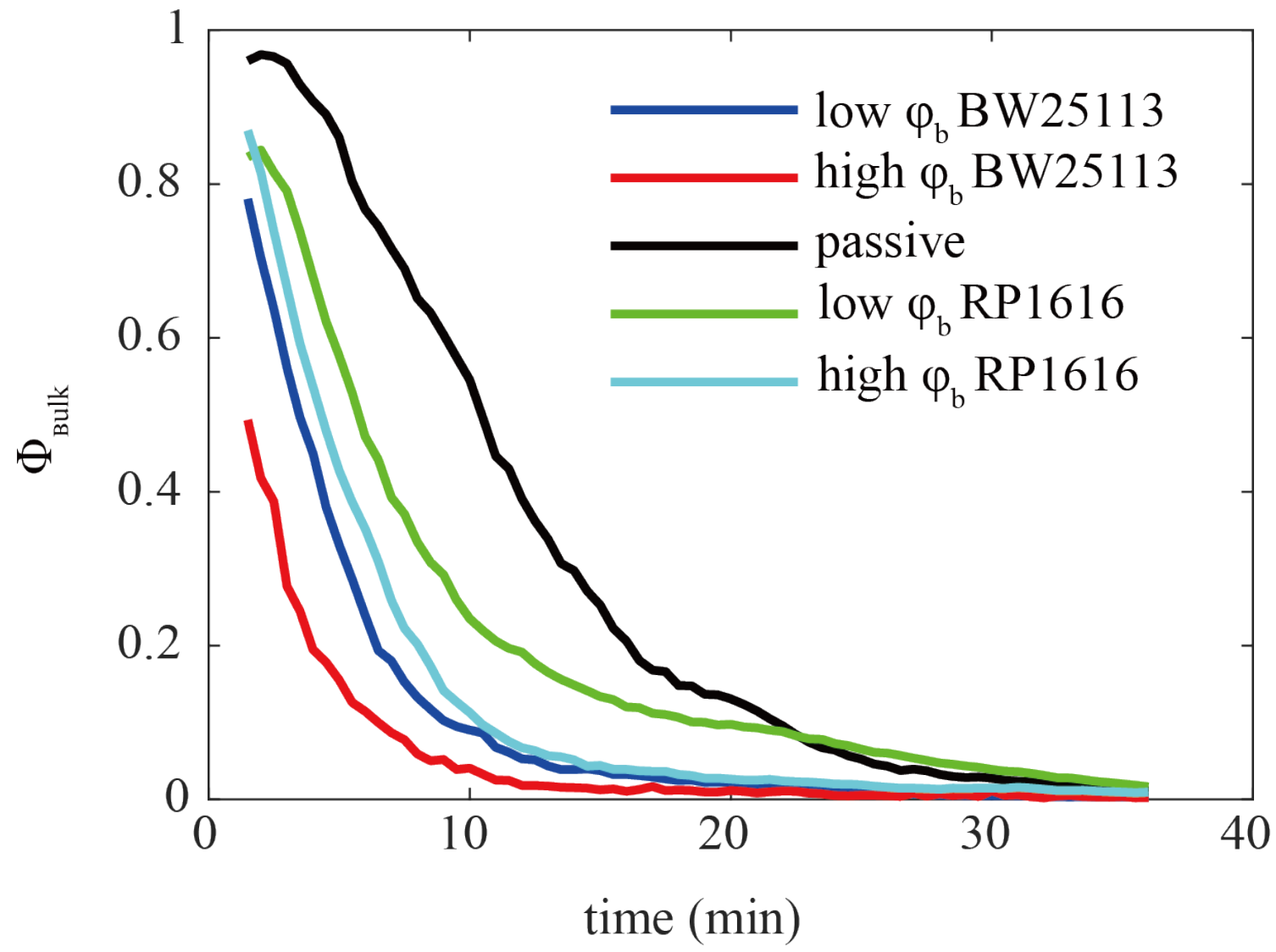

Supplementary Figure 14. Time evolution of Φ_{bulk}, for low and high bacterial concentrations in the presence of motile bacteria BW25113, non-motile bacteria RP1616, and under passive conditions.

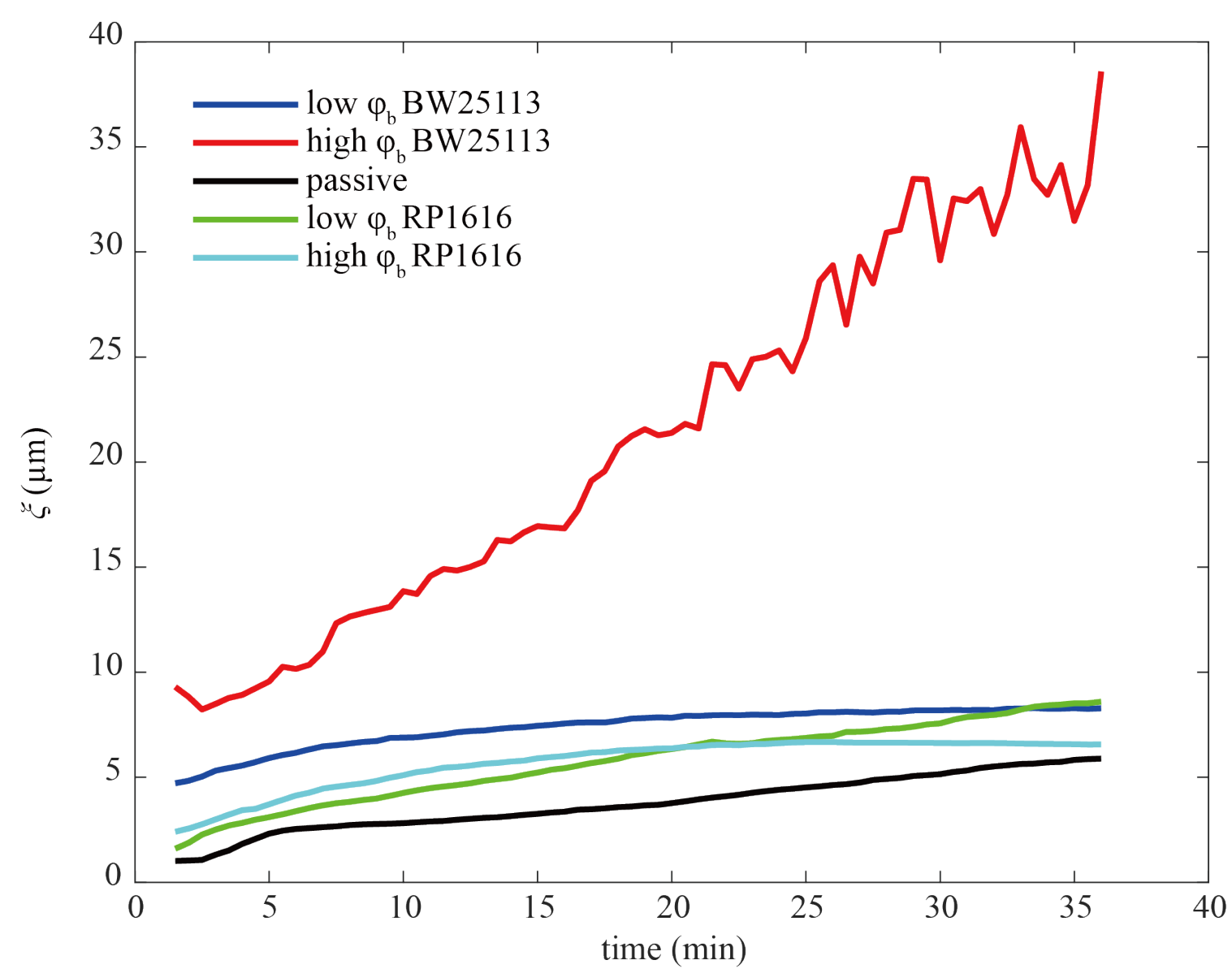

Supplementary Figure 15. Time evolution of the characteristic length of the dextran-rich phase, $\xi(t)$, measured within a 40 μm-thick region above the substrate, for low and high bacterial concentrations in the presence of motile bacteria BW25113, non-motile bacteria RP1616, and under passive conditions.

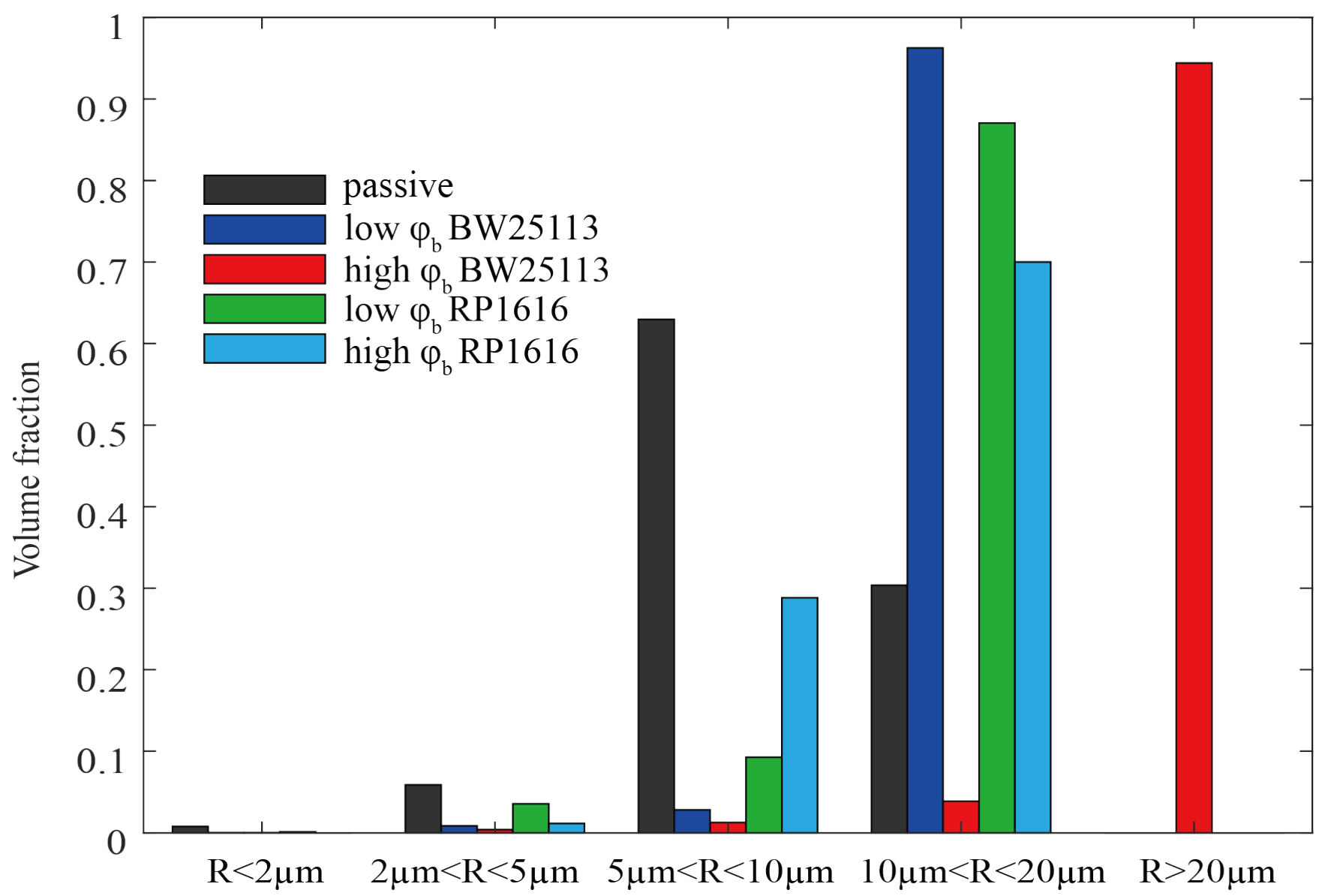

Supplementary Figure 16. Droplet size distributions at $T = 36\,\mathrm{min}$, for low and high bacterial concentrations in the presence of motile bacteria BW25113, non-motile bacteria RP1616, and under passive conditions.

E. Comparison with previous active-interface experimental systems

Previous experiments have explored several important classes of active soft interfaces. Active nematics coupled to flat liquid–liquid interfaces provide a planar interfacial setting for studying the coupling between interfacial shape, flow, and orientational order [21, 24]. In such systems, interfacial deformation arises from the coupling between the interface and the nematic director field. Our system differs in that the active phase is a dense bacterial suspension. The interface is driven by internally organized bacterial collective flows and active stresses, rather than by an explicit interfacial nematic director field.

Another related class of systems consists of active agents confined inside vesicles [15, 17, 19, 20]. Vesicle membranes possess bending elasticity, membrane tension, and thermally resolvable shape fluctuations [14]. By contrast, our droplets have liquid–liquid interfaces governed primarily by surface tension and have no membrane bending rigidity. Therefore, slender protrusions or filament-like structures are not stabilized by membrane elasticity and remain susceptible to capillary retraction and breakup. Moreover, thermal capillary fluctuations are negligible at the length scales resolved here, so the measured spectra mainly reflect activity-induced interfacial dynamics rather than equilibrium thermal fluctuations.

Our system is also distinct from active-particle droplets exemplified by quasi-two-dimensional Quincke-roller droplets [18]. In Quincke-roller droplets, the active units are electrically driven colloidal rollers confined in a quasi-two-dimensional geometry. In contrast, our droplets are three-dimensional liquid-liquid compartments containing dense bacterial suspensions, whose collective motion generates internally organized active flows. Thus, while both systems involve active matter coupled to a deformable capillary boundary, they differ in dimensionality and in the physical origin of active driving.

Section S4. SUPPLEMENTARY RESULTS FOR DROPLETS WITH LOW BACTERIAL CONCENTRATION

This section characterizes interfaces (both 3D droplet interfaces and 2D flat interfaces) in the low-activity regime, where interfacial deformations remain small and wave-like modes are present. Flow-field structure, correlation length scaling, perturbation response, and the dependence of DSF on activity are examined.

A. Flow field in low-bacterial-density droplets

To quantify the internal flow structures in low-activity droplets, we first measure the spatial velocity–velocity correlation function for droplets of different radii. The characteristic correlation length, defined as the distance at which the correlation decays to $1/e$, increases systematically with droplet size (Supplementary Figure 17a-b). This size dependence reflects a confinement effect: larger droplets accommodate longer-range flow structures, whereas smaller droplets restrict the development of vortical motion. However, even in the smallest droplets examined, where the droplet radius approaches the intrinsic vortex size, we do not observe the coherent large-scale circulation previously reported in rigidly confined systems [25, 26]. We further plot ℓ_{v} versus R in a log–log representation (Supplementary Figure 17b). A power-law fit yields $\ell_{\mathrm{v}} \sim R^{0.6}$, indicating that the correlation length grows sublinearly with droplet size.

We next analyze the power spectrum of the flow field on equatorial cross-sections for the same set of droplets, defined as $E(k) = \langle|\hat{v}(k)|^2\rangle$, where $\hat{v}(k)$ is the Fourier transform of the velocity. For droplets of radius ~ 80 μm, the spectrum follows a k^{-3} scaling (Supplementary Figure 17c), consistent with earlier observations of bacterial turbulence in quasi-two-

dimensional vesicles [19]. For much larger droplets, the spectrum displays a crossover: it approaches k^{-1} at small k and steepens toward k^{-4} at large k (Supplementary Figure 17d), consistent with the behavior of three-dimensional active turbulence [27].

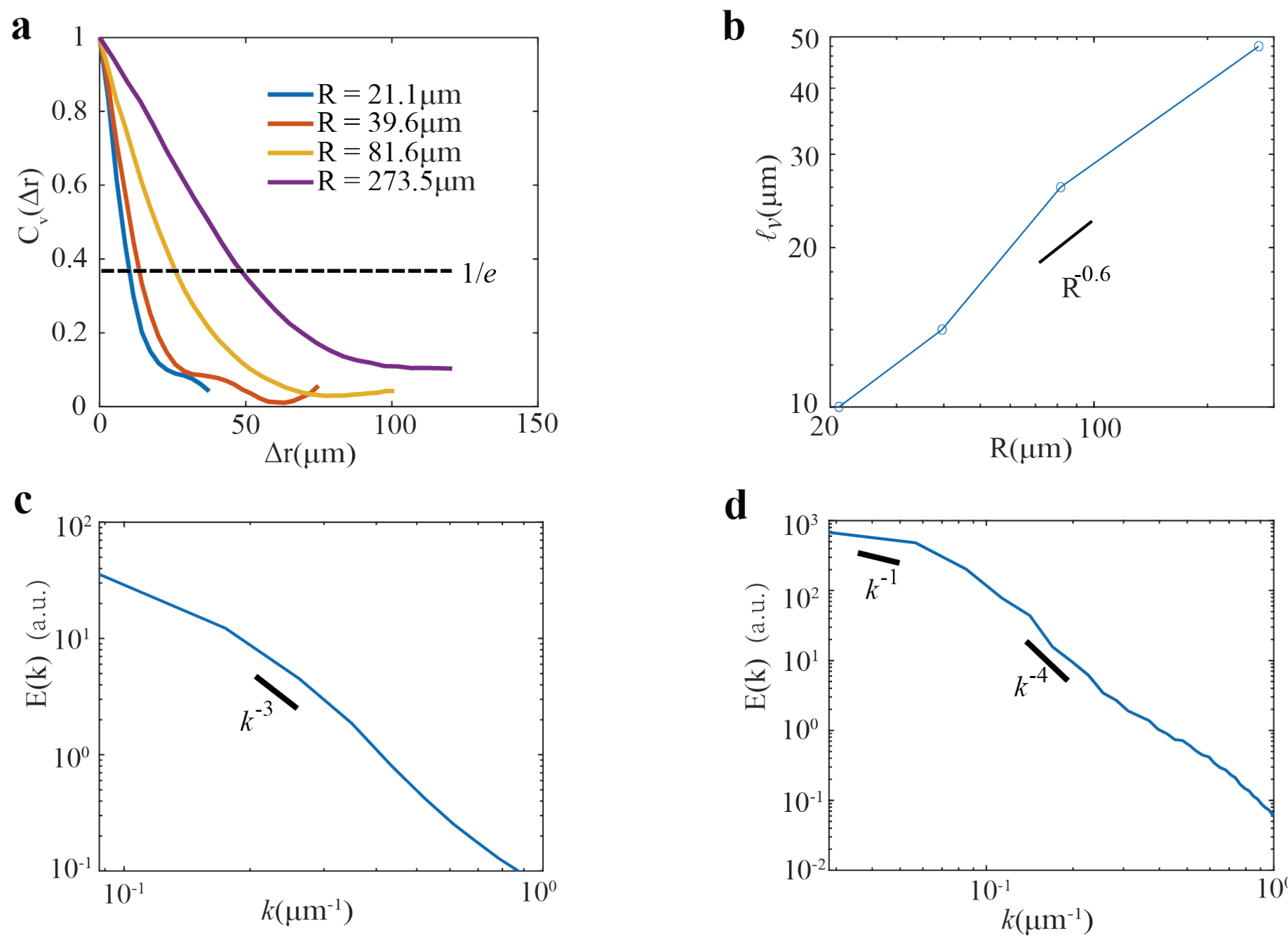

Supplementary Figure 17. (a) The spatial correlation of the flow field in droplets of different sizes. The dashed line corresponds to $1/e$.(b) The relation between $\ell_{\rm v}$ and the droplet radius R. (c) The power spectrum of the flow field on the cross section of a droplet with radius of 81.6μm. (d) The power spectrum of the flow field on the cross section of a droplet with radius of 273.5μm.

B. Mode analysis under initial perturbations

To investigate the mechanism of wave generation, we perturb spherical droplets with a prescribed spherical harmonic mode (ℓ, m) in simulations. We vary ℓ from 2 to 15 while fixing $m = 0$. A three-dimensional rendering and the corresponding cross-sectional profile are shown in Supplementary Figure 18. This axisymmetric initialization ensures that the induced interfacial deformation maps cleanly onto a two-dimensional mode with $k = \ell/R$, establishing a direct 3D–2D correspondence.

In contrast, for $m \neq 0$, the imposed non-axisymmetric perturbation leads to a more complex early-stage evolution. The initial deformation does not oscillate in a single, coherent

mode. Instead, it decomposes into a superposition of multiple modes with different phases (Supplementary Figure 19). For this reason, we focus on $m = 0$ in the main analysis to isolate the clean, single-mode behavior. Because the two-dimensional DSF measures the in-plane propagation of interfacial waves, the signals of waves passing through the cross-section fall within the envelope of the DSF. Therefore, the axisymmetric $m = 0$ mode, which oscillates within a single plane, corresponds to the outer edge of the DSF envelope.

To obtain the oscillation frequencies of different modes, we calculate the half-period duration of the corresponding mode amplitudes. For instance, under an initial perturbation with $\ell = 5, m = 0$, the half-period of the amplitude of mode $\ell = 5, m = 0$ is found to be $T = 160$, yielding an oscillation frequency $\omega = 0.02$ (Fig. 3b, Supplementary Figure 20c). By comparing the oscillation frequencies of specific modes with full simulations, we map the $\ell = 5, m = 0$ oscillation frequency onto the DSF. Applying the same calculation, we can obtain the single-mode oscillation frequencies for $\ell = 2$ to 15. The extracted frequencies of different modes coincide with the peaks of the DSF (Fig. 2h). When the initial ℓ is large, as shown in Supplementary Figure 20b, the perturbation decays to the unperturbed shape without oscillation. This corresponds to the diffusive mode whose frequency is peaked at 0 (Fig. 2h).

Similarly, in the 2D model we impose a sinusoidal perturbation onto a flat interface with different wave numbers. A perturbation with small k exhibits an oscillatory behavior, as shown in Supplementary Figure 20d and Supplementary Figure 20f , while a perturbation with larger k merely exhibits decaying, as seen in Supplementary Figure 20e and Supplementary Figure 20f. The extracted frequencies, plotted in Fig. 3d, show that the wave mechanism is robust to changes in both the dimensionality and geometry of the interface.

C. Effect of activity on the dynamic structure factor

We experimentally tune the activity of the droplets and examine its effects on the dynamic structure factor (DSF). To obtain droplets with lower activity, the active suspensions are rested in centrifuge tubes for one hour before sample preparation. This procedure reduces the maximum speed of bacterial turbulence from approximately 10 μm/s (control) to 5 μm/s. As shown in Supplementary Figure 21a, droplets with insufficient activity do not exhibit distinct propagating branches in the DSF. Conversely, at higher activity ($v_{\max} \approx 15$ μm/s),

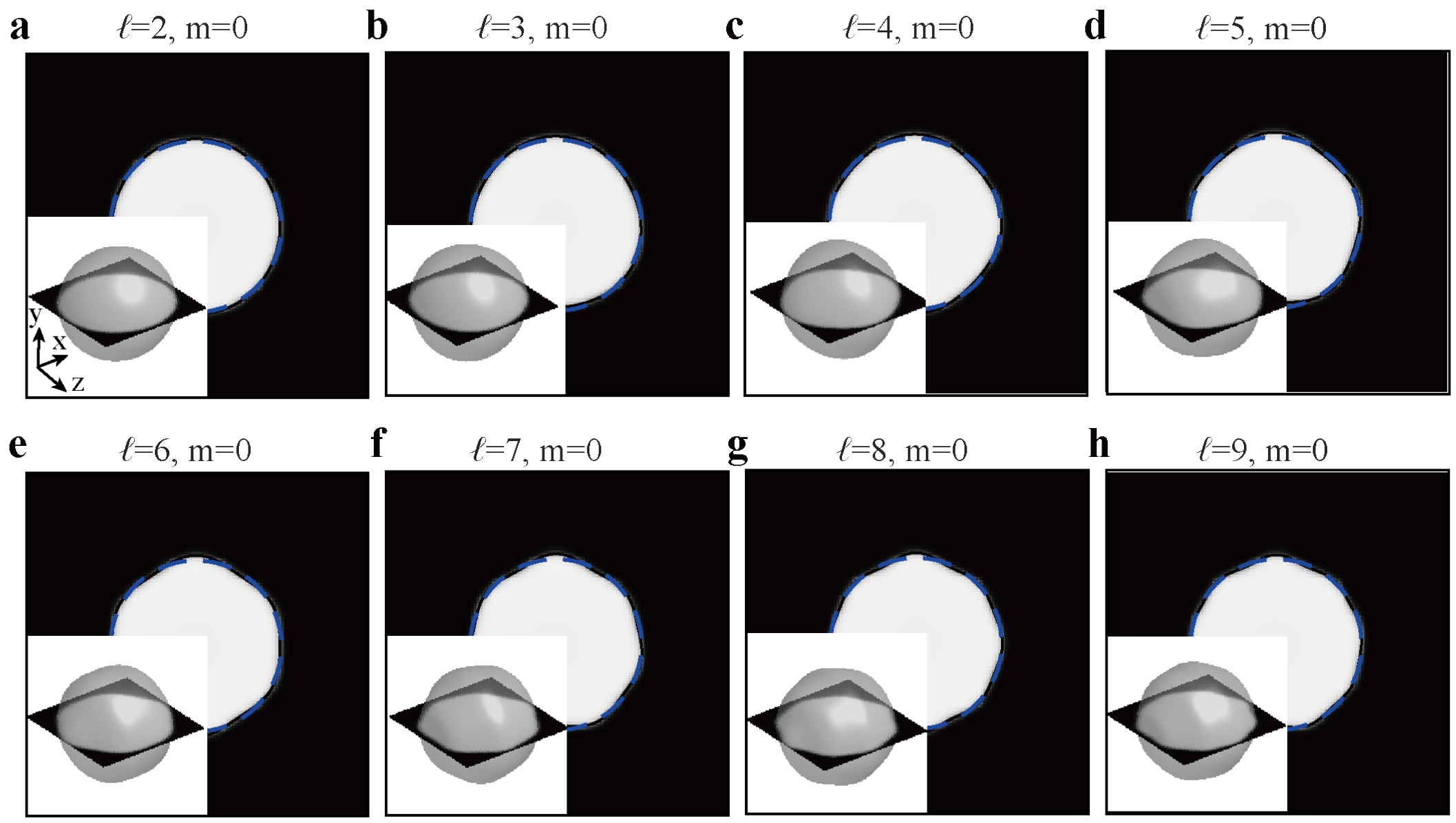

Supplementary Figure 18. Initial three-dimensional morphology of the perturbation and its observed cross-section in the analysis with (a) $\ell = 2, m = 0$, (b) $\ell = 3, m = 0$, (c) $\ell = 4, m = 0$, (d) $\ell = 5, m = 0$, (e) $\ell = 6, m = 0$, (f) $\ell = 7, m = 0$, (g) $\ell = 8, m = 0$, (h) $\ell = 9, m = 0$.

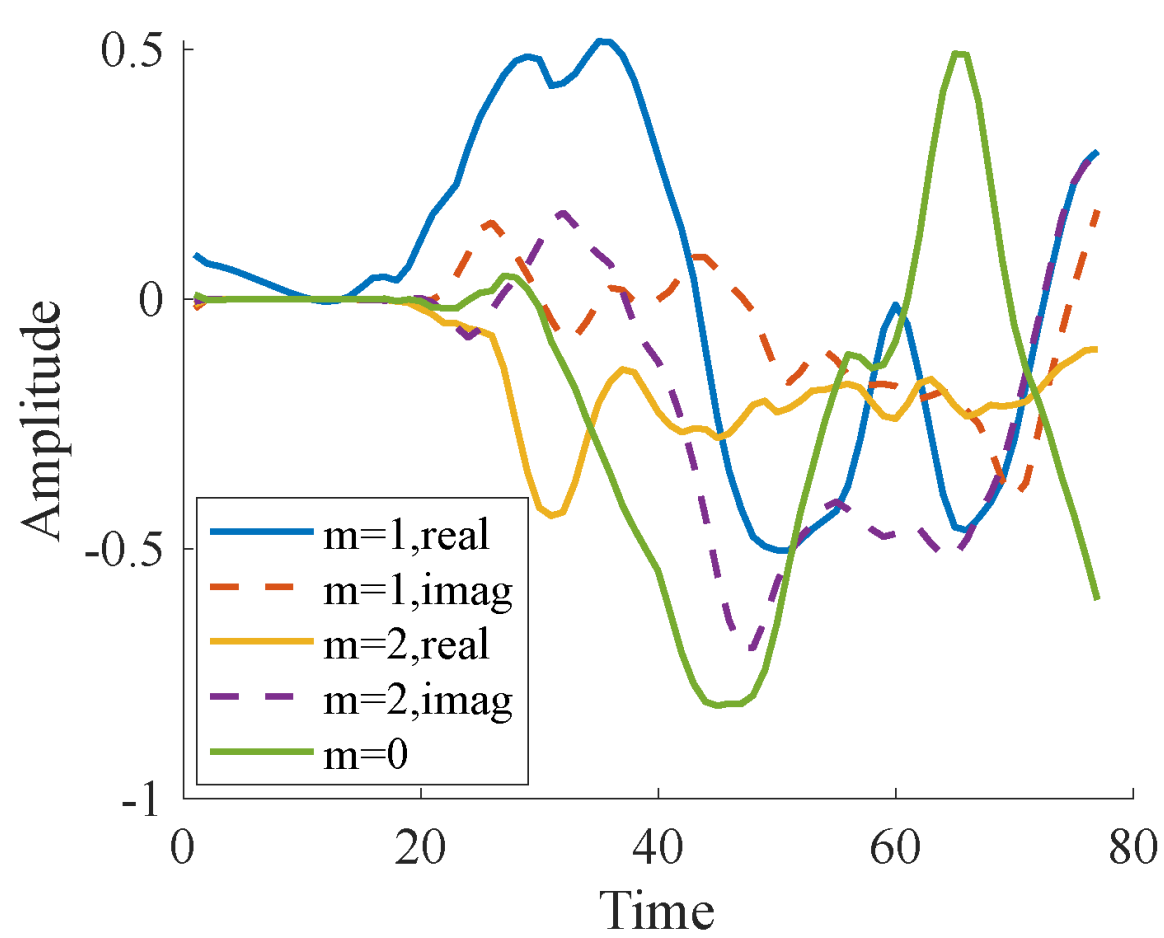

Supplementary Figure 19. Temporal evolution of mode amplitudes for $\ell = 3$ with different m components, initialized from the $(\ell, m) = (3, 1)$ perturbation. The simulation parameters are set to their default values.

the DSF (Supplementary Figure 21c) again lacks clear wave signatures, indicating that excessive activity suppresses coherent oscillations. Hence, propagating interfacial waves emerge only within an intermediate range of activity (Supplementary Figure 21b), where

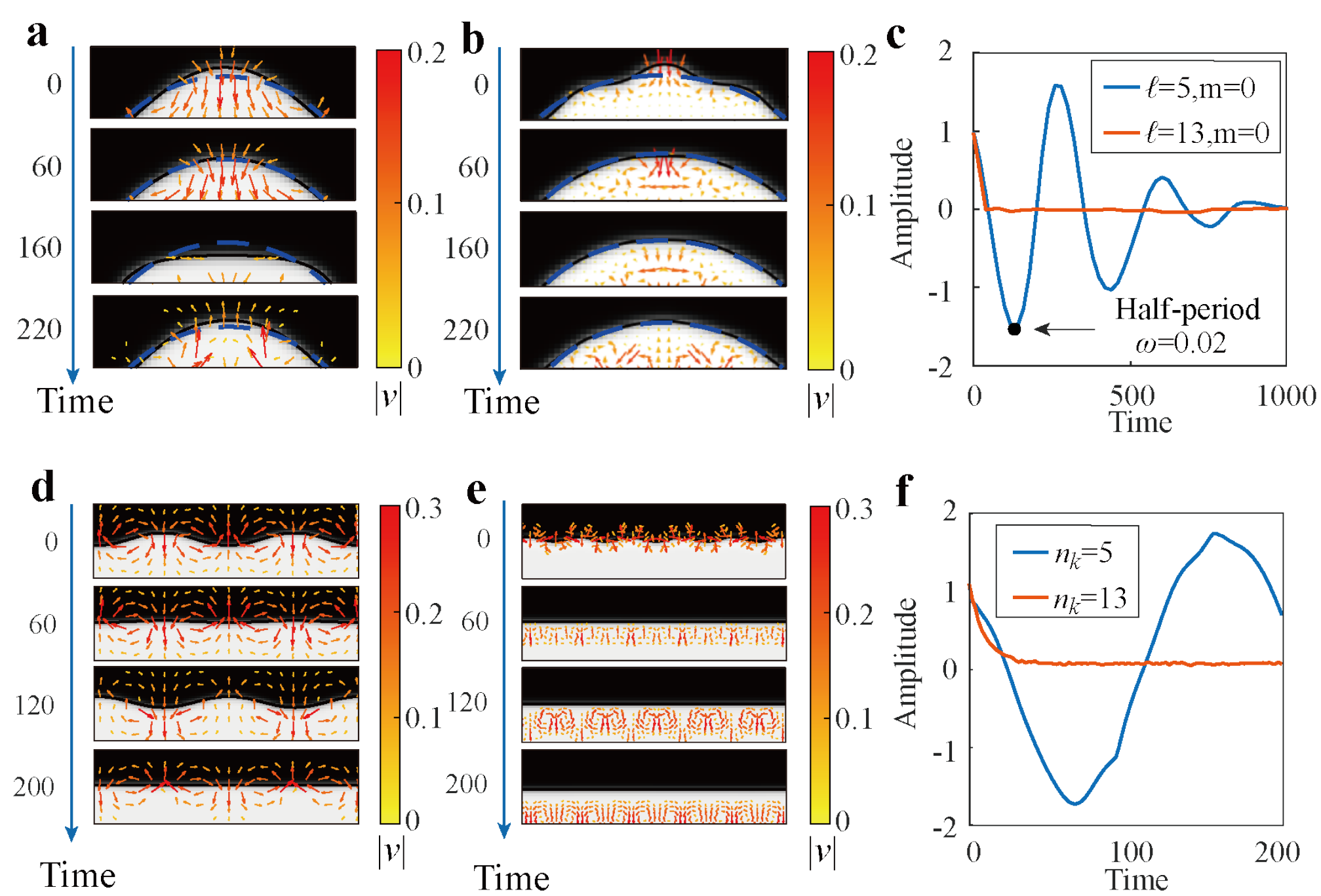

Supplementary Figure 20. (a) Interface evolution and associated flow field resulting from an initial perturbation with ($\ell = 5, m = 0$) on a spherical interface. The blue dashed line indicates the unperturbed shape. (b) Interface evolution and associated flow field resulting from an initial perturbation with ($\ell = 13, m = 0$) on a spherical interface. The blue dashed line indicates the unperturbed shape. (c) Temporal evolution of the amplitudes of the ($\ell = 5, m = 0$) and ($\ell = 13, m = 0$) modes. The black dot denotes the corresponding frequency appearing in Fig. 2h. For (a-c), the setup as the same as that shown in Fig. 2h and Fig. 3a-b in the main text. (d) Interface evolution and associated flow field resulting from an initial perturbation with ($n_k = 5$) on a flat interface. (e) Interface evolution and associated flow field resulting from an initial perturbation with ($n_k = 13$) on a flat interface. (f) Temporal evolution of the amplitudes of the ($n_k = 5$) and ($n_k = 13$) modes. For (d-f), the flat-interface setup is the same as that shown in Fig. 3d in the main text. The simulation parameters, both in 2D and 3D, are set to their default values.

the DSF shows a clear dispersive branch at small k that vanishes beyond a characteristic cutoff k_c (dashed line).

To further validate this nonmonotonic dependence, we perform both 3D and 2D simulations by varying the activity parameters $|\nu_2|$ and ζ, while keeping the flow correlation length ℓ_v fixed. At small $|\nu_2|$, the interface remains quiescent and the DSF shows no oscillatory

feature (Supplementary Figure 22a,d). For intermediate activity ($\nu_2 = -0.1$), propagating waves appear at small k, whereas the spectral power at larger k concentrates near zero frequency (Supplementary Figure 22b,e). When $|\nu_2|$ increases further, the interface becomes highly distorted, and the DSF signal broadens and loses coherence (Supplementary Figure 22c,f). A similar trend is reproduced in 2D simulations (Supplementary Figure 23), confirming that the wave-sustaining regime occurs only at intermediate activity, consistent with experiments.

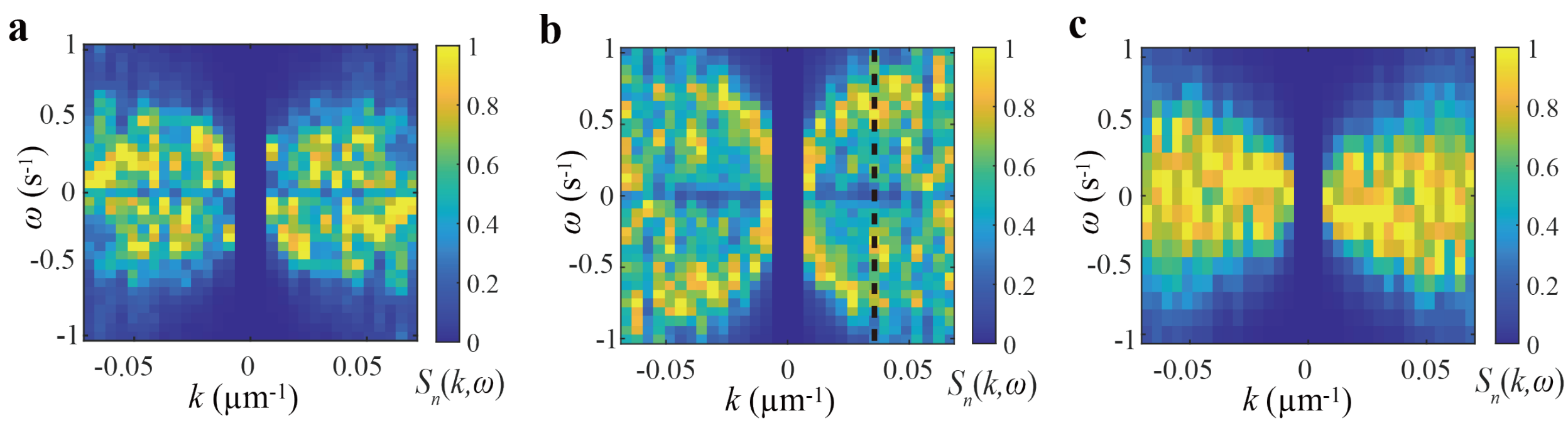

Supplementary Figure 21. Normalized dynamic structure factors (DSF) with varying activity in experimental droplets. (a) Droplet with a maximum speed of 5 μm/s. (b) Droplet with a maximum speed of 10 μm/s. The dashed line marks the critical wave vector k_c. (c) Droplet with a maximum speed of 15 μm/s.

D. Effect of correlation length on the dynamic structure factor

In both experiments and simulations, the transition from propagating to diffusive behavior occurs around a characteristic wavenumber k_c. For $k < k_c$, the DSF exhibits finite-frequency peaks corresponding to propagating waves, whereas for $k > k_c$, the fluctuations collapse to zero frequency. We find that k_c scales inversely with the flow correlation length $\ell_{\rm v}$, indicating that wave propagation is sustained only over length scales larger than the typical vortex size.

To test this relationship, we vary $\ell_{\rm v}$ in simulations by tuning ζ while fixing ν_2, thereby maintaining constant activity. In 3D simulations, k_c consistently remains on the order of $1/\ell_{\rm v}$ (Supplementary Figure 24). In 2D simulations, which allow a wider range of $\ell_{\rm v}$, k_c increases monotonically with $\ell_{\rm v}^{-1}$ (Supplementary Figure 25). Together, these results indicate that the

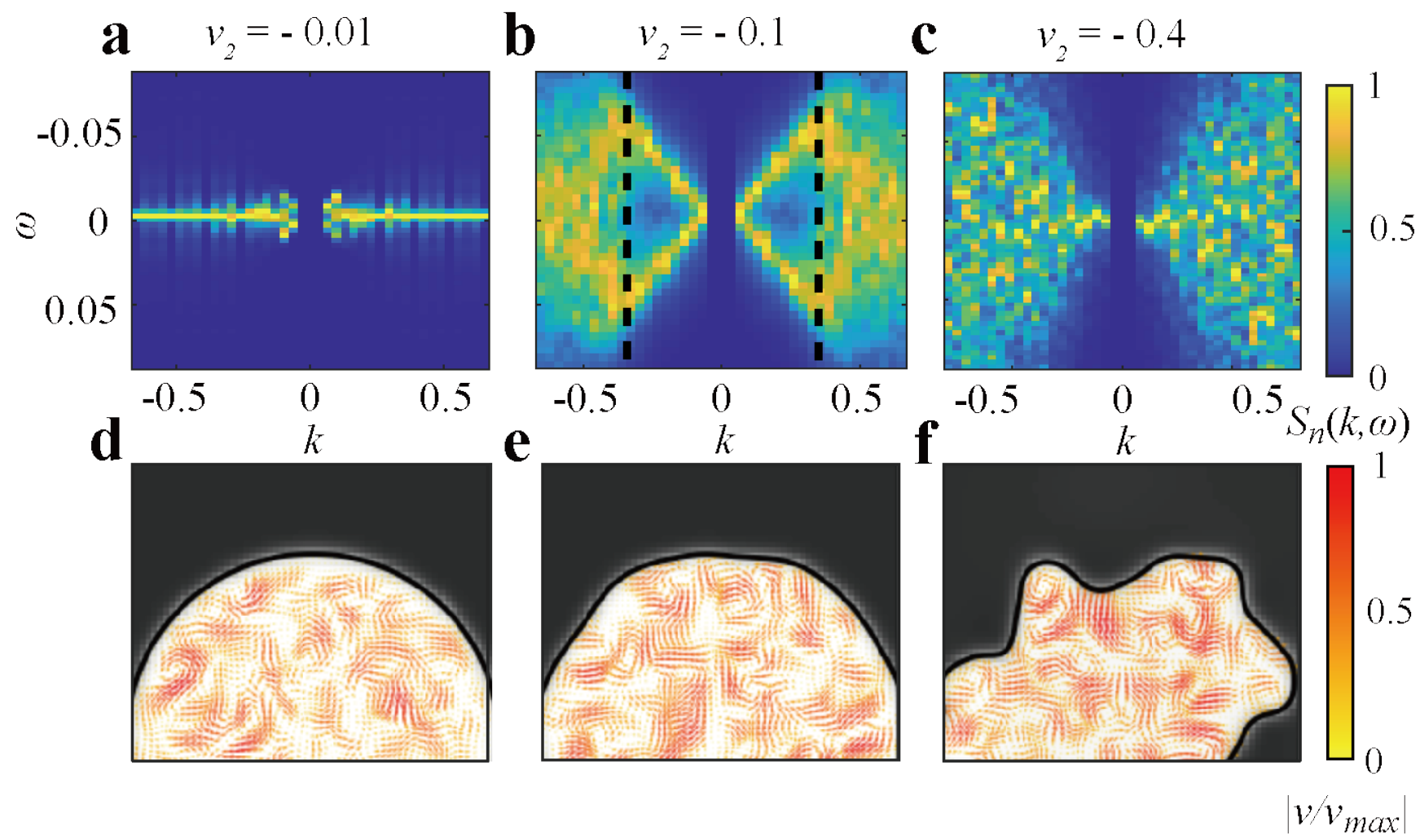

Supplementary Figure 22. Normalized dynamic structure factors (DSF) for droplets with varying ν_2 in 3D simulations. (a-c) Normalized DSF for droplets with different values of ν_2: (a) $\nu_2 = -0.01$, (b) $\nu_2 = -0.1$, and (c) $\nu_2 = -0.4$. The dashed line in (b) indicates the critical wave vector k_c. (d-f) Corresponding snapshots of the equatorial flow fields for each condition. Simulation parameters: Correlation length ζ is set to 0.01, 0.1, and 0.4 for (a), (b), and (c), respectively. Other parameters are set to default values. Color bars represent normalized velocity magnitude $|v|/v_{\max}$.

correlation length correlates with the upper bound of wave propagation, linking the spatial scale of turbulence to the spectral cutoff observed in the DSF.

E. Control simulations with Ornstein-Uhlenbeck active stresses

To test whether random active forcing alone is sufficient to generate the observed propagating modes, we perform simulations driven by Ornstein-Uhlenbeck stresses. As shown in Supplementary Figure 26, the dynamic structure factor does not display distinct propagating peaks modes, rather, it shows a peak at $\omega = 0$. This supports the interpretation that the propagating interfacial waves are not produced by generic random forcing, but arise from the negative-viscosity mechanism, which simultaneously selects coherent bulk vortical flow and weakly damped interfacial modes.

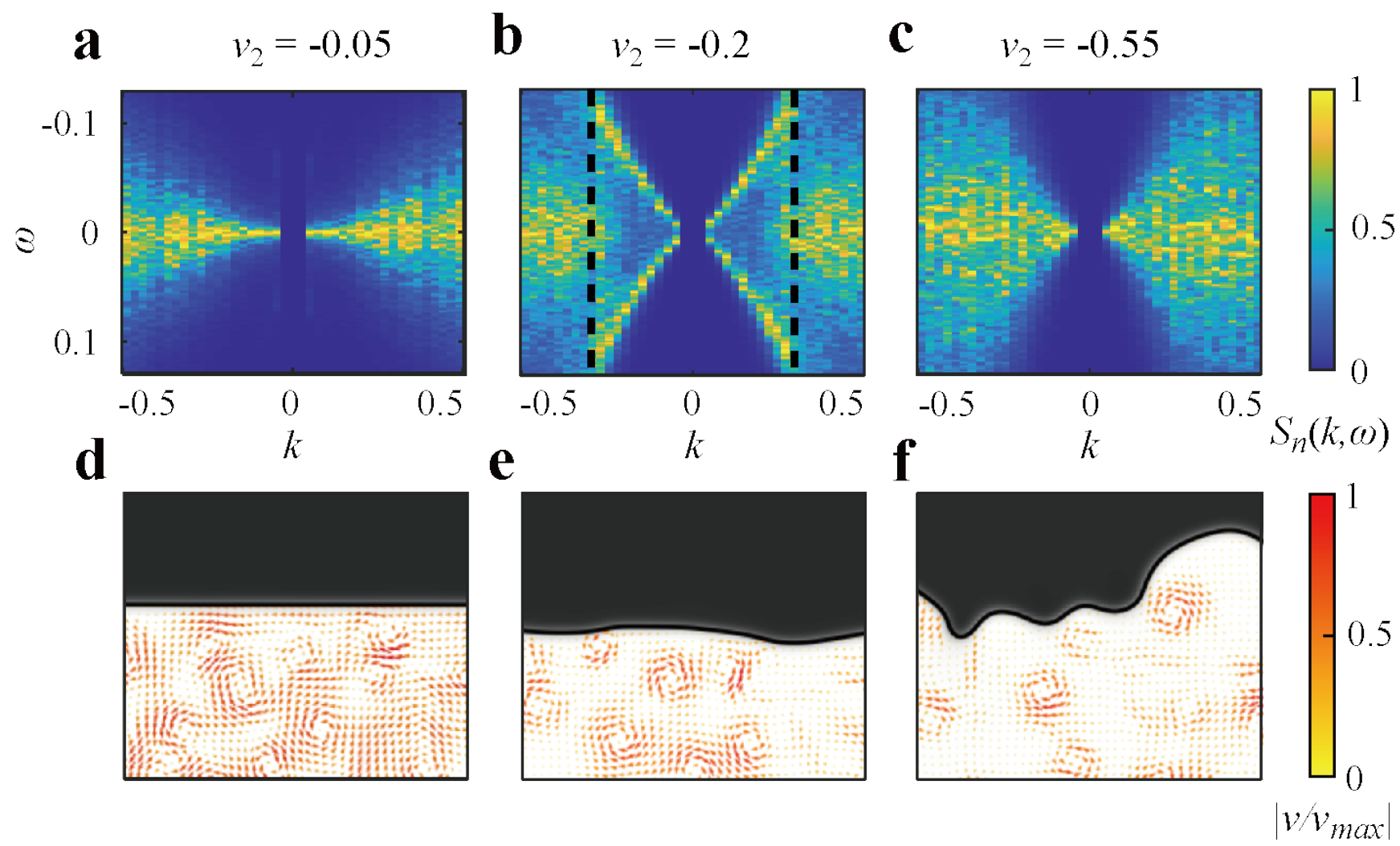

Supplementary Figure 23. Normalized dynamic structure factors (DSF) with varying ν_2 in 2D flat systems. (a-c) Normalized DSF for droplets with different values of ν_2: (a) $\nu_2 = -0.05$, (b) $\nu_2 = -0.2$, and (c) $\nu_2 = -0.55$. The dashed line in (b) indicates the critical wave vector k_c. (d-f) Corresponding snapshots of the flow fields at each ν_2. Simulation parameters: Correlation length ζ is set to 0.05, 0.2, and 0.55 for (a), (b), and (c), respectively. Other parameters are set to default values. Color bars represent normalized velocity magnitude $|v|/v_{\max}$.

F. Theory of activity-induced propagating waves on a fluctuating interface

We derive the dispersion relation of an active–passive interface and discuss how propagating and diffusive regimes emerge, as well as the role of higher-order viscosity in stabilizing the system.

We consider a two-dimensional geometry consisting of a passive fluid (region 1, $y > 0$) and an active fluid (region 2, $y < 0$) , separated by a nearly flat interface located at $y = 0$ with surface tension γ_0 (Supplementary Figure 27). This simplified geometry captures the essential physics of the interfacial waves observed in our simulations while allowing for an analytical treatment. We denote the local deformation of the interface by $h(x, t)$, which is assumed to be small compared with the system size so that linearization is valid.

To describe the flow fields in both fluids, we start from the linearized Toner–Tu–Swift–Hohenberg (TTSH) equations, neglecting the fourth-order viscous term for simplicity. The

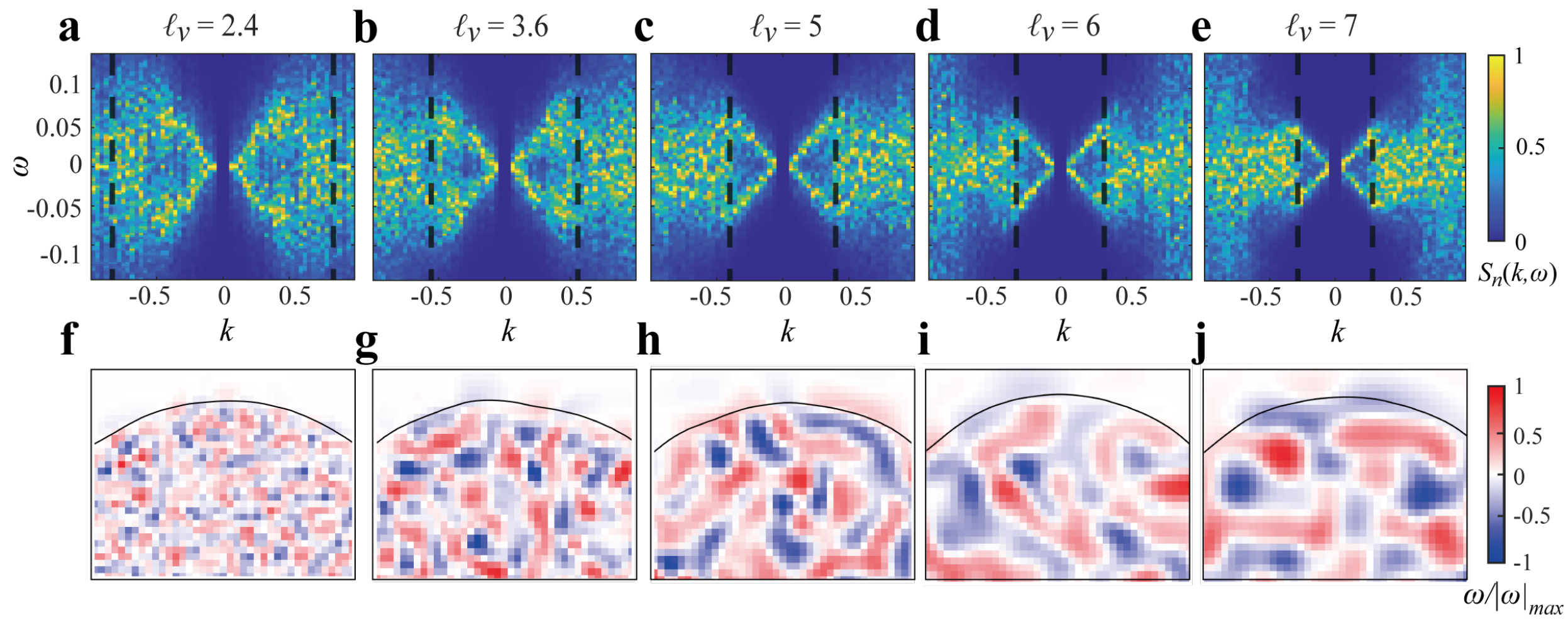

Supplementary Figure 24. Normalized dynamic structure factors (DSF) and the corresponding vorticity fields for different correlation lengths $\ell_{\rm v}$ in a droplet with $R = 32$. Top row, (a–e): normalized DSF $S_\eta(k,\omega)$ for $\ell_{\rm v} = 2.4, 3.6, 5, 6$, and 7, respectively. The dashed lines mark the cutoff wavevector k_c. Bottom row, (f–j): corresponding instantaneous vorticity fields $\omega/\omega_{\rm max}$, where the black solid line denotes the droplet interface. In the simulation, ν_2 is fixed at -0.1, and $\ell_{\rm v} = 2.4, 3.6, 5, 6, 7$ are obtained by setting $\zeta = 0.036, 0.064, 0.1, 0.144, 0.196$, respectively. The remaining parameters are set to their default values.

velocity in each fluid, labeled by $i = 1, 2$, satisfy

$$\partial_t \mathbf{v}^i = -\nabla P^i - \alpha_i \mathbf{v}^i + \nu_i \nabla^2 \mathbf{v}^i. \tag{20}$$

and the condition of incompressibility

$$\nabla \cdot \mathbf{v}^i = 0 \tag{21}$$

Here, P^i the pressure, α_i the linear friction or activity parameter and ν_i is the effective viscosity. The top fluid (region 1) is passive, so we set $\alpha_1 = 0$, whereas the bottom fluid (region 2) is active with $\alpha_2 = \alpha$. Across the interface, continuity of velocity and stress balance impose the following boundary conditions [21, 28–30]:

$$\begin{aligned} [\mathbf{v}]_0 &= 0, \\ [T_{xy}]_0 &= 0, \\ [T_{yy}]_0 &= -\gamma_0 \partial_x^2 h(x,t), \end{aligned} \tag{22}$$

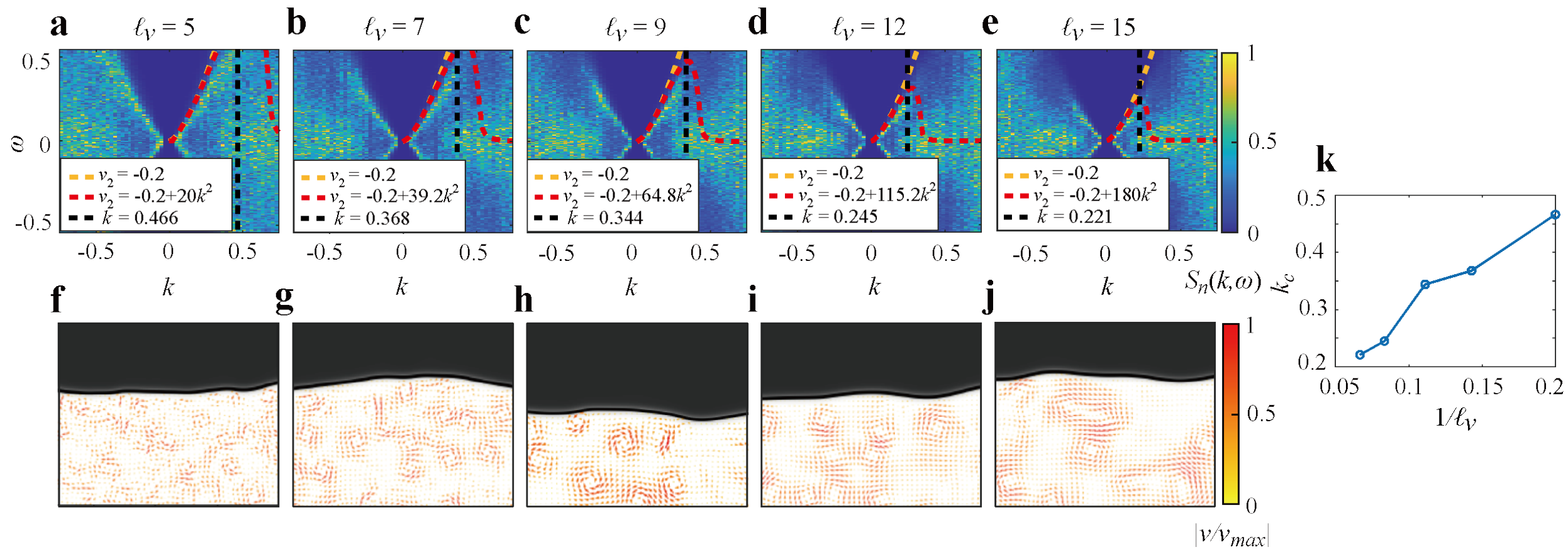

Supplementary Figure 25. Normalized dynamic structure factors (DSF) with varying correlation lengths ℓ_{v} in the 2D flat system and the corresponding transition wave vector (black dashed line). (a–e) Normalized dynamic structure factors (DSFs) with varying ℓ_{v}. (f–j) Snapshots of the equatorial flow fields corresponding to (a–e). (k) Relationship between the transition wavenumber k_c and the correlation length ℓ_{v}. The red and yellow dashed lines correspond to the analytical result with and without considering the higher-order viscosity. Parameters: $\nu_1 = 0.5$, $\nu_2 = -0.2$, $\gamma_0 = 23.6$; (a) $\ell_{\mathrm{v}} = 5$, (b) $\ell_{\mathrm{v}} = 7$, (c) $\ell_{\mathrm{v}} = 9$, (d) $\ell_{\mathrm{v}} = 12$, (e) $\ell_{\mathrm{v}} = 15$. In the simulation, ν_2 is fixed at -0.2, and $\ell_{\mathrm{v}} = 5, 7, 9, 12, 15$ are obtained by setting $\zeta = 0.072, 0.128, 0.2, 0.288, 0.392$, respectively. Other parameters are set to their default values. Color bar indicates normalized velocity magnitude $|v|/v_{\max}$.

where for any function $s(x, y)$ we define the jump across the interface as $[s(x)]_0 \equiv s(x, y = 0^-) - s(x, y = 0^+)$. Here, $\boldsymbol{T}^i$ is the stress tensor in each fluid, given by [21, 28–30]

$$\boldsymbol{T}^i = \nu_i \left(\nabla \mathbf{v}^i + \nabla {\mathbf{v}^i}^T \right) - P^i \boldsymbol{I}. \tag{23}$$

To linear order, the interface height is related to the local velocity through [21, 28–30]

$$\partial_t h(x, t) = v_y(x, y = 0, t) \tag{24}$$

Following Refs. [21, 28–30] , we Fourier transform Eqs. (20)-(21) in x, and eliminate $\hat{v}_x$ and $\hat{P}$ in favor of $\hat{v}_y$, thereby obtaining a closed equation for $\hat{v}_y(k, y, t)$

$$\left(\partial_y^2 - k^2 \right) \left(\partial_y^2 - \beta_i^2 k^2 \right) \hat{v}_y^i(k, y, t) = 0 \tag{25}$$

where $\beta_i = \sqrt{1 + \frac{i\widetilde{\omega} + \alpha_i}{\nu_i k^2}}$, $\widetilde{\omega}$ is the complex frequency. Similarly procedure allows elimination

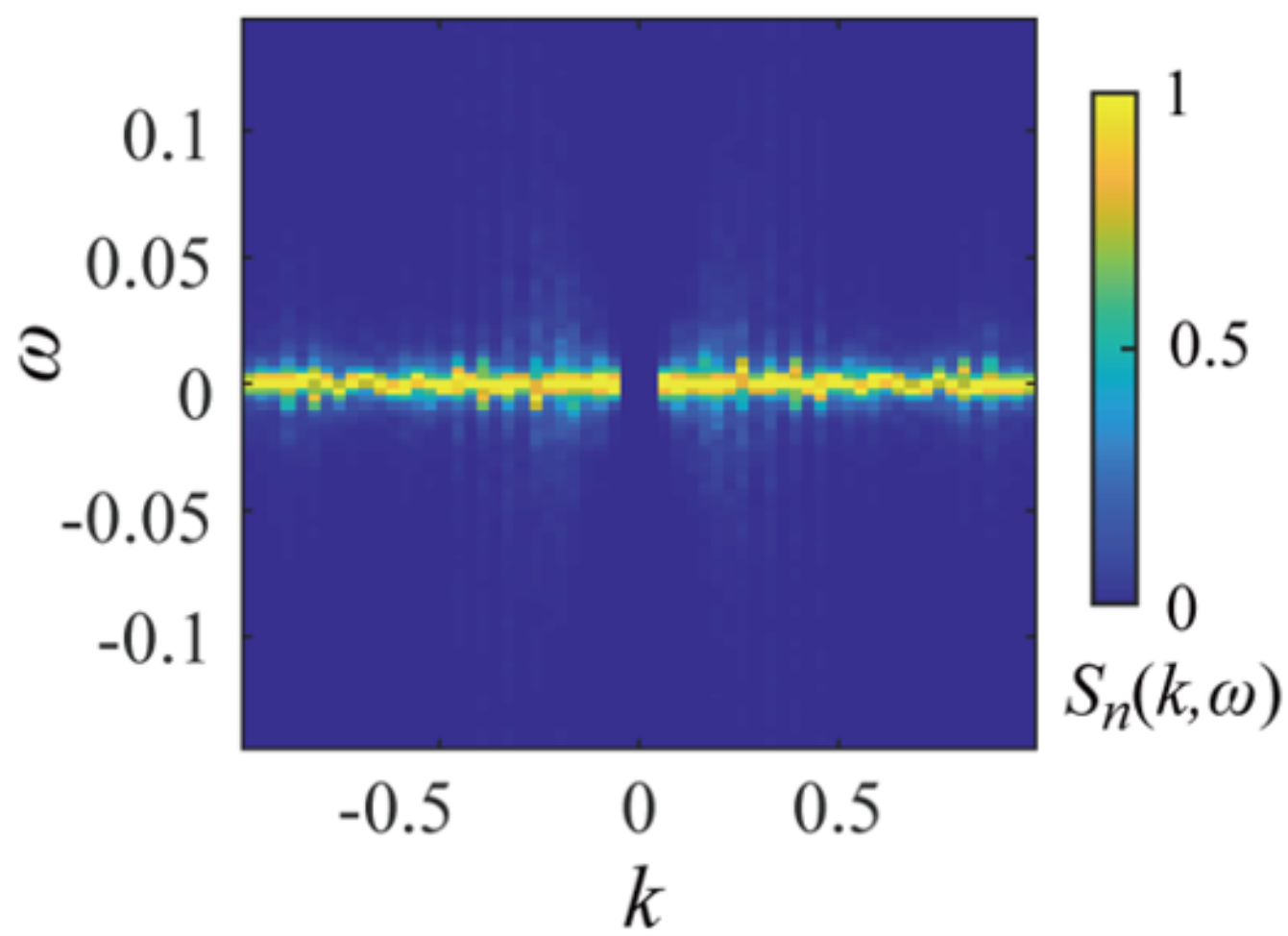

Supplementary Figure 26. Rescaled dynamic structure factor $S_n(k,\omega)$ from simulations with Ornstein-Uhlenbeck random active stresses.

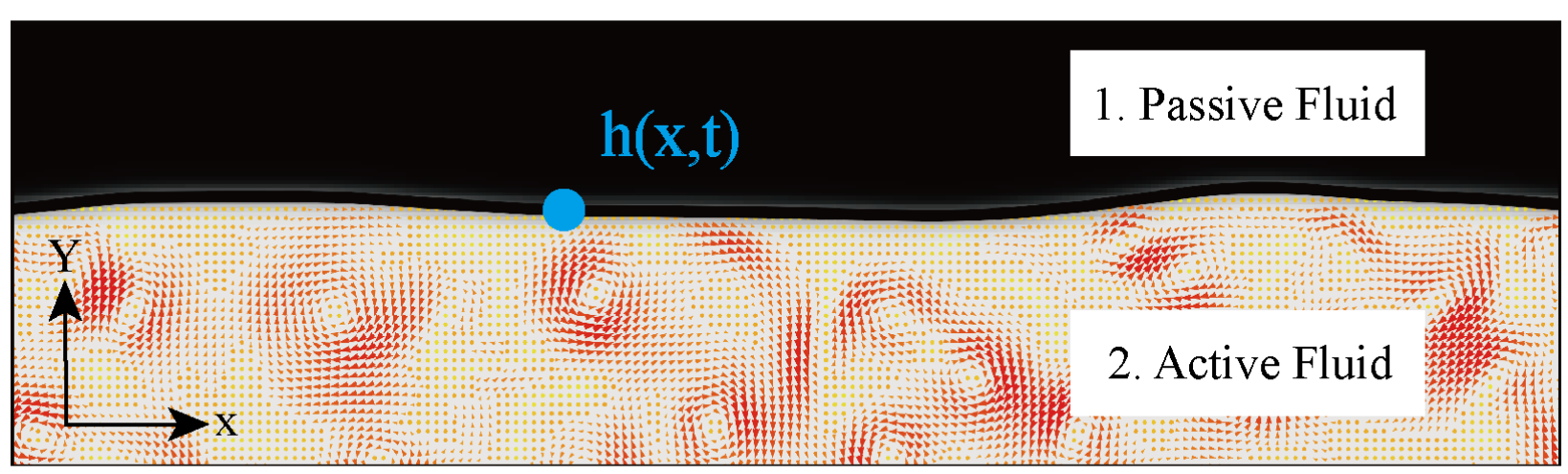

Supplementary Figure 27. A snap of 2D flat interface separating an upper passive and a bottom TTSH fluid.

of $\hat{v}_x$ and $\hat{P}$ in the boundary conditions Eq. (22), giving

$$\begin{aligned} [\hat{v}_y]_0 &= 0, \\ [\partial_y \hat{v}_y]_0 &= 0, \\ \left[\nu_i(\partial_y^2 + k^2)\hat{v}_y\right]_0 &= 0, \\ \left[\frac{\nu_i}{k^2}\left(\partial_y^2 - 2k^2 - \beta_i^2 k^2\right)\partial_y \hat{v}_y\right]_0 &= \gamma_0 k^2 \hat{h}. \end{aligned} \tag{26}$$

The solution to Eq. (25) with boundary conditions given by Eq. (26) is given by

$$\hat{v}_y(k,t) = -\frac{\left[\nu_1(1+\beta_1) + \nu_2(1+\beta_2)\right]\gamma_0\, k\hat{h}(x,t)}{f_1\nu_1^2 + f_2\nu_1\nu_2 + f_3\nu_2^2} \tag{27}$$

with

$$\begin{aligned} f_1 &= (-1 + 3\beta_1 + \beta_1^2 + \beta_1^3), \\ f_2 &= 2 - (\beta_1 + \beta_2) + \beta_1^2 + \beta_2^2 + 4\beta_1\beta_2 + \beta_1\beta_2(\beta_1 + \beta_2), \\ f_3 &= (-1 + 3\beta_2 + \beta_2^2 + \beta_2^3). \end{aligned} \tag{28}$$

Substituting Eq. (27) into Eq. (24), we obtain the dispersion relation of the interface shown in Supplementary Figure 28a yellow curve

$$i\tilde{\omega} = -\frac{[\nu_1(1+\beta_1) \; + \nu_2(1+\beta_2)]\,\gamma_0\, k}{f_1\nu_1^2 + f_2\nu_1\nu_2 + f_3\nu_2^2}. \tag{29}$$

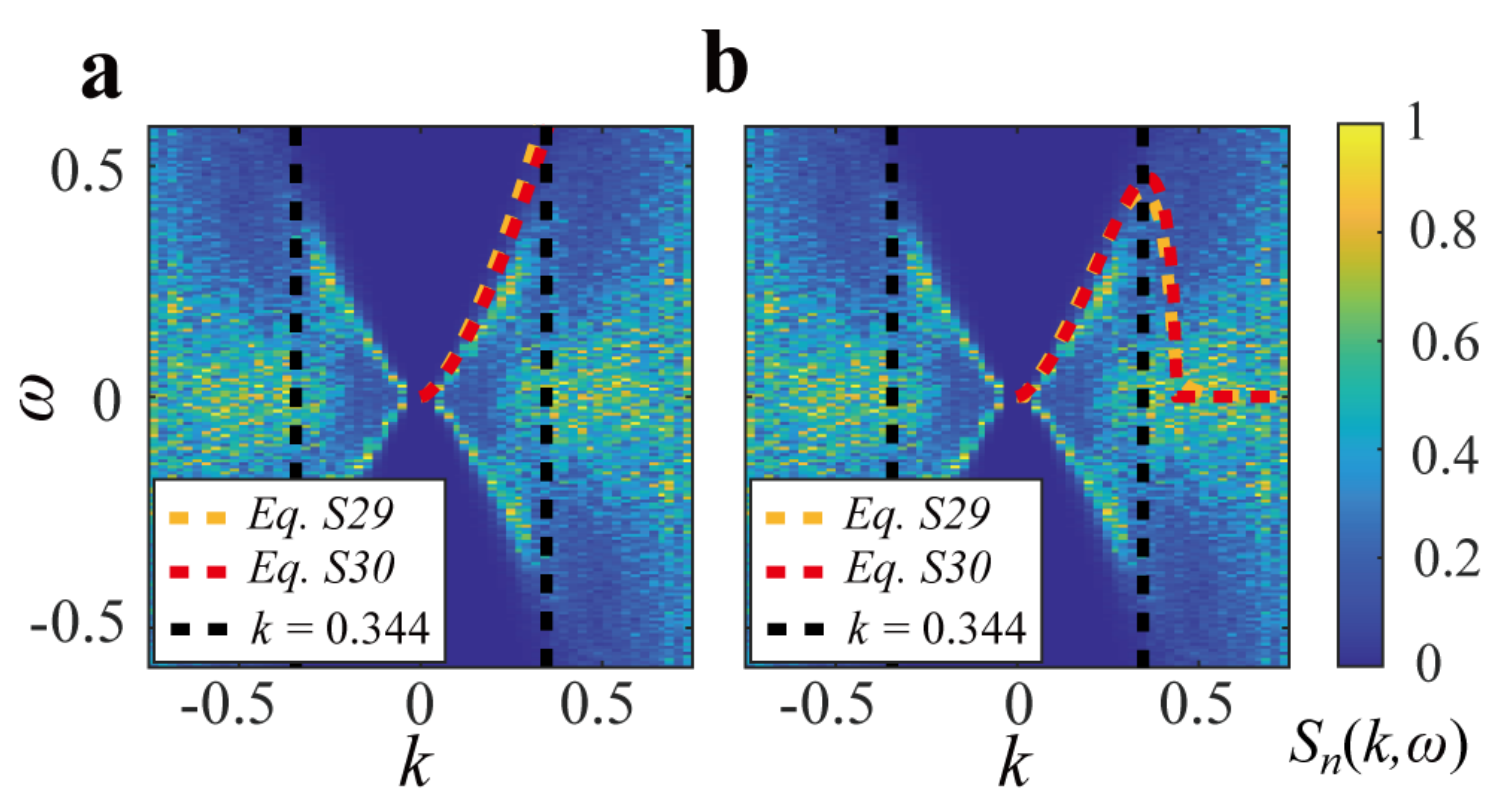

Supplementary Figure 28. Comparison between theoretical predictions and 2D simulation results of the normalized dynamic structure factor (DSF). The yellow and red dashed lines denote the analytical dispersion relations given by Eq. (29) and Eq. (30), respectively. The lines in (a) and (b) show the analytical dispersion relation without and with the inclusion of higher-order viscosity, respectively. The yellow line in (b) is obtained by substituting $\nu_2'(k) \equiv \nu_2(1 - 4\ell_{\rm v}^2 k^2)$ into Eq. (29). The dashed lines mark. the critical wave vector k_c. The simulation parameters are: $\alpha = -0.01$, $\nu_1 = 0.5$, $\nu_2 = -0.2$, $\ell_{\rm v} = 9$, and $\gamma_0 = 23.6$.

The effective damping coefficient α provides a k-independent contribution to all modes, hence mainly influences the system's behavior at the scale of the system size, whereas mesoscopic scales with large k are mainly dominated by the effective viscosity and interfacial tension. For simplicity, we drop the terms involving α. We further consider the case when $|\nu k^2| \gg |\tilde{\omega}|$. Using Taylor expansion, we obtain $\beta_1 \approx 1 + \frac{i\tilde{\omega}}{2\nu_1 k^2}$, $\beta_2 \approx 1 + \frac{i\tilde{\omega}}{2\nu_2 k^2}$. In this case, the resulting dispersion relation in Eq. (29) reduces to:

$$(i\tilde{\omega})^2 + \frac{1}{\tau_v} i\tilde{\omega} + \frac{1}{\tau_0^2} = 0, \tag{30}$$

involving two characteristic time scales: the capillary oscillation period $\tau_0 = \sqrt{\frac{3}{\gamma_0 k^3}}$ and the viscous relaxation time $\tau_v = \left(\frac{2(\nu_1+\nu_2)k^2}{3}\right)^{-1}$. Supplementary Figure 28 shows quantitative agreement among Eq. (29), Eq. (30) and the simulation results for $k < k_c$, validating our neglect of α and the assumption $|\nu k^2| \gg |\tilde{\omega}|$.

Activity enters the interface dynamics by regulating the viscous damping time τ_v. At low activity, $|\nu_2| \ll \nu_1$, viscous damping dominates and no oscillation occurs (Supplementary Figure 21a, Supplementary Figure 22a,d, Supplementary Figure 23a,d). An intermediate activity can adequately offset the exterior viscous damping, such that $\nu_1 + \nu_2 \sim 0$, rendering the interface underdamped, and restore the inertia effects to support propagating waves (Supplementary Figure 21b, Supplementary Figure 22b,e, Supplementary Figure 23b,e). At very high activity, $|\nu_2| \gg \nu_1$, τ_v becomes sufficiently negative, providing a negative damping that destabilizes the interface and drives exponential growth of fluctuations. In full simulations, such high activity also enhances interfacial deformations, introducing nonlinear effects that suppress propagating waves (Supplementary Figure 21c, Supplementary Figure 22c,f, Supplementary Figure 23c,f).

Both experiments and simulations reveal a clear cutoff wavenumber k_c: for $k > k_c$, the DSF no longer exhibits propagating signals (Fig. 2d,h; Supplementary Figure 21b; Supplementary Figure 22b; Supplementary Figure 23b; Supplementary Figure 28b). Consistently, simulations with different initial perturbations show that modes with $k > k_c$ in 2D and $\ell/R > k_c$ in 3D decay monotonically without oscillation (Fig. 3b; Supplementary Figure 20b,e). Our linear theory also predicts a cutoff wavenumber $k_c' = \frac{3\gamma_0}{(\nu_1+\nu_2)^2}$., obtained from the critical damping condition $\tau_v = \tau_0/2$. However, this estimate is larger than the k_c found in simulations (Supplementary Figure 28). Such discrepancy likely arises from higher-order viscous and nonlinear effects that provide additional damping not captured by the linear theory. To incorporate these effects, we phenomenologically introduce an effective fourth-order viscosity, resulting in a k-dependent effective viscosity

$$\nu_2'(k) = \nu_2(1 - 4\ell_{\mathrm{v}}^2 k^2). \tag{31}$$

This correction implies that the active (negative) driving weakens as k increases. The chosen form of $\nu_2'(k)$ is by no means arbitrary, as it gives a characteristic vortex size $2\ell_{\mathrm{v}}$ according to the Swift-Hohenberg pattern forming framework [31], consistent with our simulations.

Accordingly, the viscous time scale becomes

$$\tau_v = \left[\frac{2(\nu_1 + \nu_2'(k))k^2}{3}\right]^{-1}.$$

By imposing the condition $\tau_v = \tau_0/2$, we obtain a cutoff wavenumber k_c':

$$\nu_1 + \nu_2(1 - 4\ell_{\mathrm{v}}^2 k_c'^2) = \sqrt{\frac{3\gamma_0}{k_c'}}. \tag{32}$$

With this correction, the dispersion relation and k_c' from the linear theory both agree with the simulations (red curves in Fig. 3d and Supplementary Figure 28b). Equation (31) further shows that as k increases, the effect of negative viscosity gradually weakens. Once the wavelength becomes comparable to the vortex size, viscous dissipation dominates and oscillations cease. Consequently, in both experiments and simulations, wave propagation disappears on length scales set by the velocity correlation length ℓ_{v} (Fig. 2d,h,c; Supplementary Figure 24; Supplementary Figure 25). In 2D simulations, increasing ℓ_{v} systematically lowering k_c (Supplementary Figure 25). However, the precise value of k_c reflects the combined effects of ν_1, ν_2, γ_0, and ℓ_{v}, with the latter scaling as $\sqrt{\zeta/|\nu_2|}$ and thus being coupled to activity.

G. Comparison with other active-interface wave mechanisms

Propagating or oscillatory interfacial modes have been reported in several active soft-interface systems, but the underlying mechanisms differ from the one considered here. In active nematic liquid–liquid interfaces, propagating waves are commonly interpreted through the coupling between interfacial deformation, flow, and the nematic director field [21, 32]. In such descriptions, the orientational degree of freedom is an explicit dynamical variable: director distortions generate active stresses that drive interfacial motion, while interfacial deformation and flow feed back on the director field. Recent theories show that nonreciprocal coupling between the interface and the nematic director can give rise to non-inertial propagating modes.

Active vesicles provide another distinct route to propagating dynamics [20]. In that case, the boundary is an elastic membrane rather than a simple liquid–liquid interface. Contractile stresses, membrane bending elasticity, membrane tension, and area constraints can together

generate active shape modes. Thus, the relevant restoring forces are membrane-based, and the wave dynamics are strongly tied to the mechanics of the vesicle boundary.

The mechanism considered here follows a different coarse-grained description. Rather than introducing an explicit interfacial nematic director field, we describe the bacterial phase through an active velocity field with an activity-modified viscous response. Within this framework, collective bacterial motion reduces the effective damping of the droplet phase. When this active contribution nearly compensates the viscous damping from the surrounding passive phase, long-wavelength interfacial modes become effectively underdamped and can propagate even in the low-Reynolds-number regime.

This coarse-grained description does not exclude orientational effects at the microscopic level. For extensile bacteria, local orientational correlations and shear-induced alignment can contribute to active stresses that oppose passive viscous stresses. After coarse graining, these effects may enter as a negative effective viscosity in the TTSH-type description. Thus, orientational correlations are incorporated through the bulk active rheology of the bacterial suspension, rather than through an explicitly modeled interfacial director field.

This modeling choice is consistent with the experimental regime analyzed here. The propagating waves are observed at low bacterial volume fraction, where the droplets remain approximately spherical and no persistent nematic ordering localized at the droplet interface is resolved within our imaging resolution (Supplementary Figure 2). The same damping-compensation mechanism also captures the main spectral features of the observed waves: propagating modes occur primarily at long wavelengths, disappear above a cutoff wavenumber k_c, and show a cutoff scale associated with the active-flow correlation length ℓ_{v}. Together with the agreement between droplet and flat-interface simulations, these results indicate that the low-amplitude interfacial waves can be described by a minimal bulk-rheological mechanism without explicitly introducing nonreciprocal coupling between an interfacial director field and the interface.

Section S5. SUPPLEMENTARY RESULTS FOR DROPLETS WITH HIGH BACTERIAL CONCENTRATION

This section describes the nonlinear deformations emerging at high bacterial concentration, where droplets exhibit strong global distortions and multiscale protrusions. Temporal

evolution, flow alignment, and characteristic morphological features are analyzed.

A. Global and local deformations of droplets with high bacterial concentration

Small droplets with high bacterial density exhibit global elongation. As shown in Supplementary Figure 29, the droplet exhibits pronounced stretching and twisting motions. During the stretching process, directed flow pushes the interface outward, driving protrusions on the interface which, in turn, can guide active jets that continuously push the interface forward (Supplementary Figure 31 and Supplementary Figure 32). Specifically, the droplet aspect ratio can exceed π, corresponding to the geometrical threshold associated with the Rayleigh-Plateau instability criterion. The persistence of these elongated shapes suggests that active forcing may prevent the capillary breakup expected in the passive case. We emphasize that the detailed mechanism remains to be resolved, but two plausible effects may contribute. First, bacterial turbulent flows drive the interface from inside the droplet and can generate unsteady pressure fields and recirculating flows. Because the droplet volume is conserved and the internal flow is incompressible, local interfacial deformations are coupled to the global pressure and velocity field. This coupling may allow internally generated active fluctuations to partially counteract the capillary pressure gradients that would otherwise promote necking and breakup. Second, active flows near the interface may generate tangential interfacial motion. In a spatially modulated protrusion, such tangential motion could redistribute fluid and momentum along the interface and smooth axial variations between necks and bulges.

Compared to small droplets, larger droplets display multiple protrusions emerging from their surfaces, as illustrated in Supplementary Figure 33. This difference is reflected in the surface-area dynamics. In Fig. 4e in the main text, we demonstrate 11 different droplets with different effective radius. As shown in Supplementary Figure 34, both small and large droplets exhibit oscillations in surface area. However, small droplets occasionally relax to near-spherical shapes, with Δ approaching zero, indicating that protrusion formation is intermittent and often followed by global relaxation. By contrast, large droplets maintain $\Delta \approx 0.6$, indicating persistent deformation. This suggests that the apparent occurrence frequency of these protrusions is higher in larger droplets: because of their larger interfacial area, different regions of the same droplet can be activated by different correlated flow domains, so protrusions can appear successively or simultaneously at multiple locations. In

this sense, large droplets remain in a continuously deformed state, whereas small droplets more often alternate between elongated and nearly spherical morphologies.

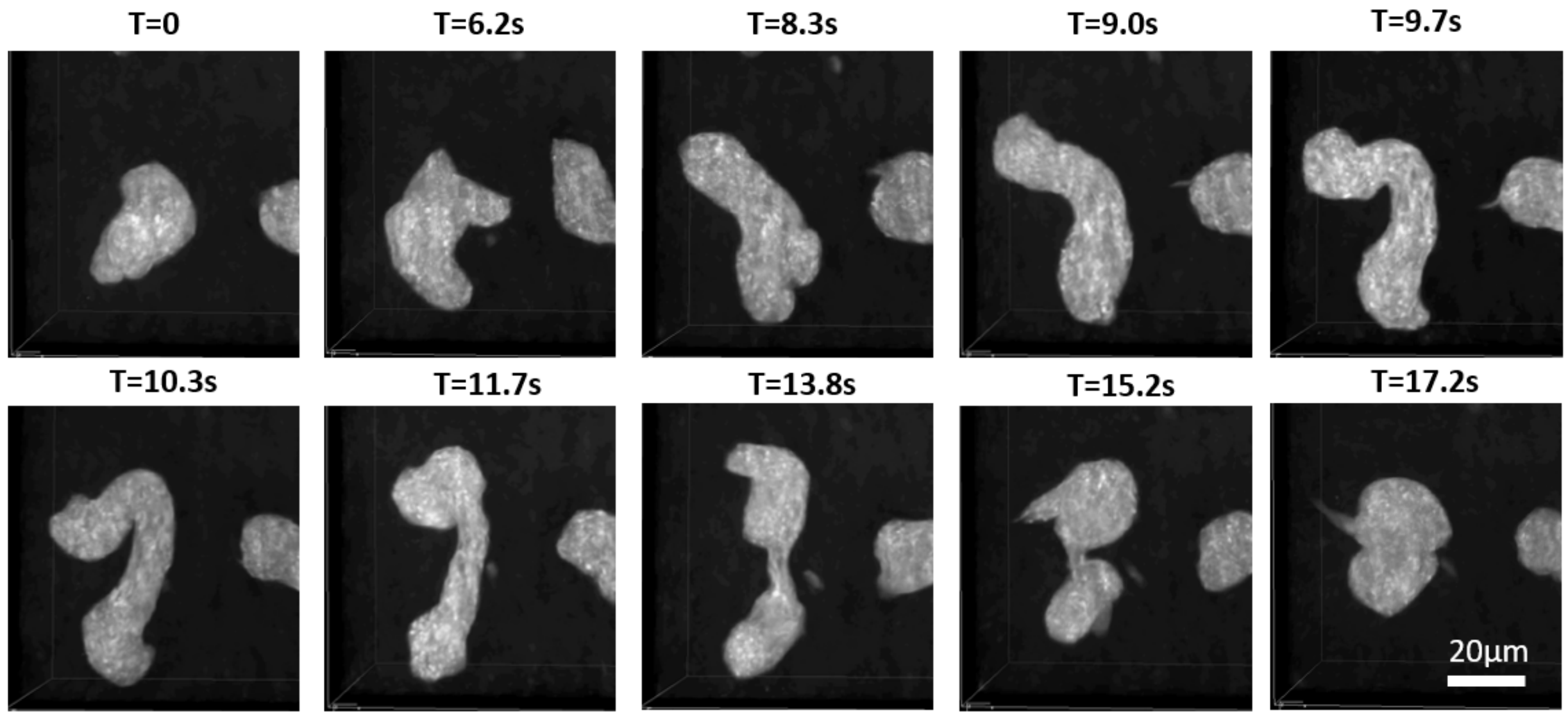

Supplementary Figure 29. Time series of an elongated droplet. The droplet undergoes stretching and twisting in this process.

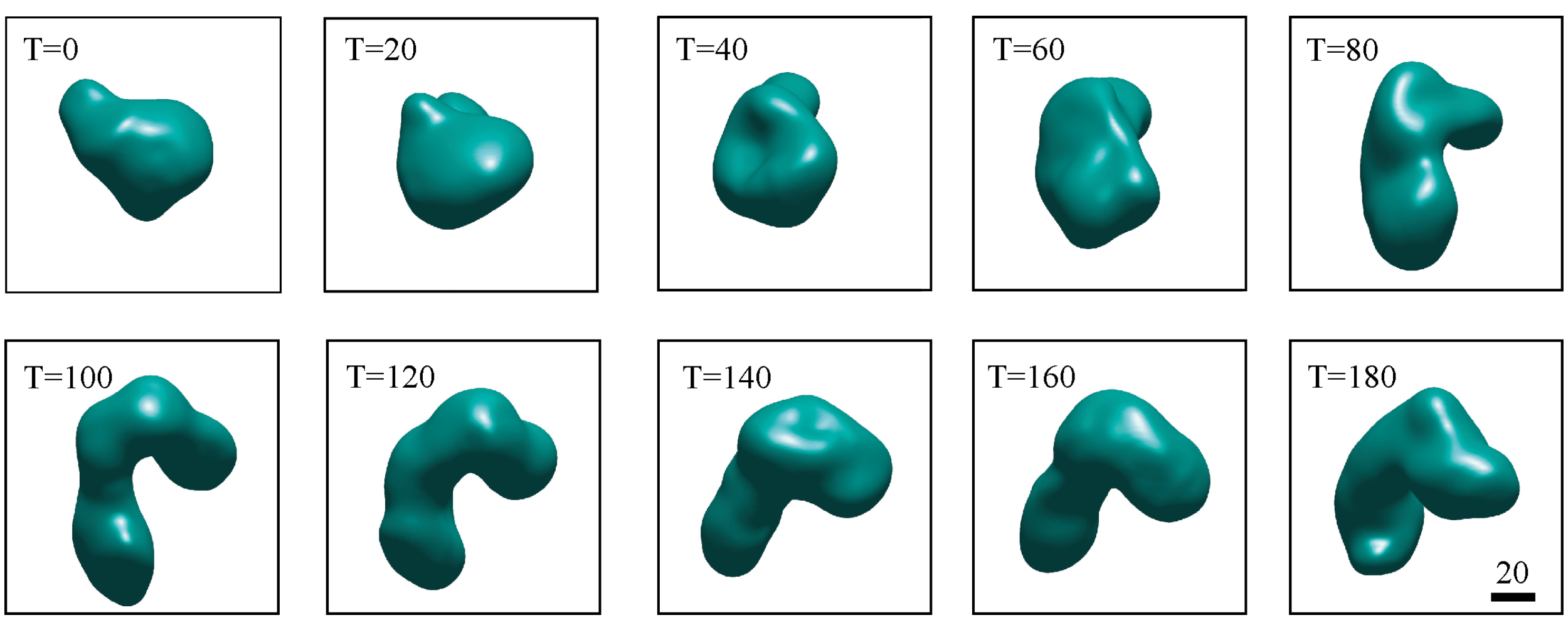

Supplementary Figure 30. Time series of an elongated droplet in simulation. In this simulation, $\nu_2 = -0.5, \zeta = 4$, the other parameters are set to their default values.

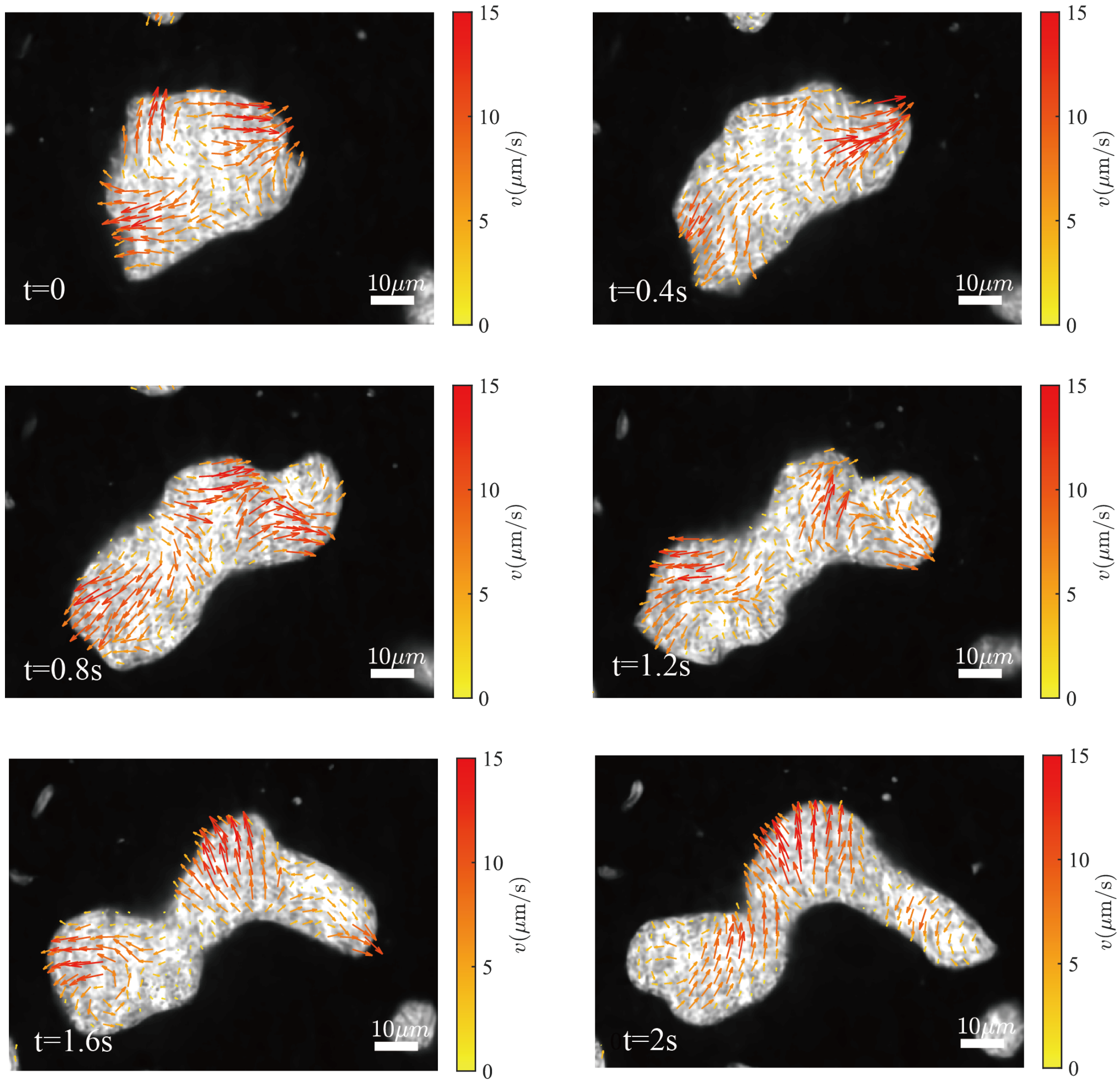

Supplementary Figure 31. Time series of the flow field at the cross section of an elongated droplets in experiment. The jet flows coincide with the generation of protrusions.

B. Formation and dynamics of thin filaments

At high bacterial concentration, slender filament-like protrusions are occasionally observed (Fig. 4d and Supplementary Figure 35). The resulting width is approximately several microns, corresponding to the order of a few bacterial body widths. These thin filaments are shorter-lived than the mesoscopic protrusions. In representative events such as those shown in Supplementary Movie 8, they persist on the order of ten seconds before retracting, breaking, or detaching from the main droplet body. Their occurrence is more heterogeneous from droplet to droplet, likely reflecting small variations in interfacial tension and bacterial

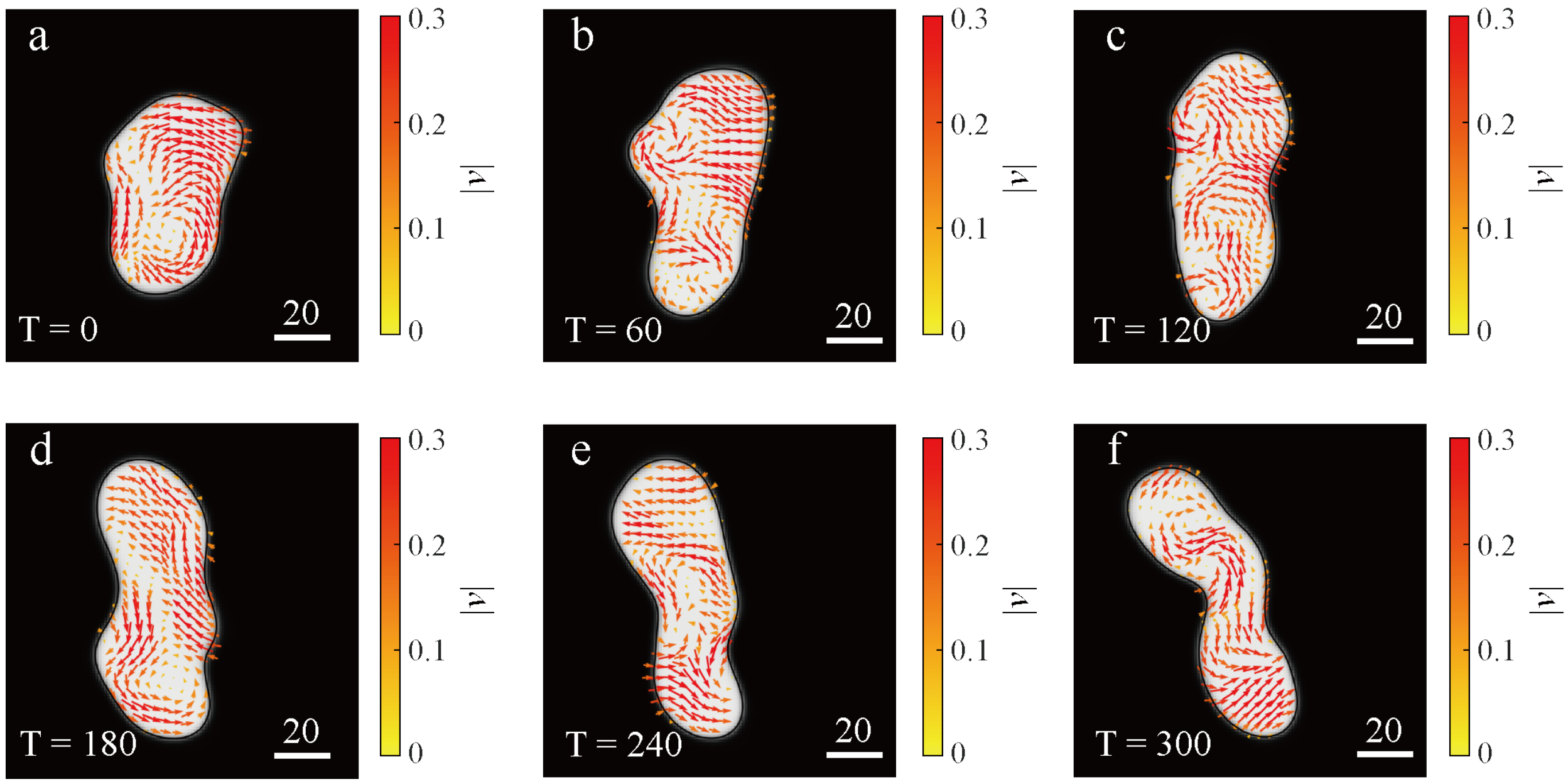

Supplementary Figure 32. Time series of the flow field at the cross section of an elongated droplets in simulation. The jet flows coincide with the generation of protrusions.

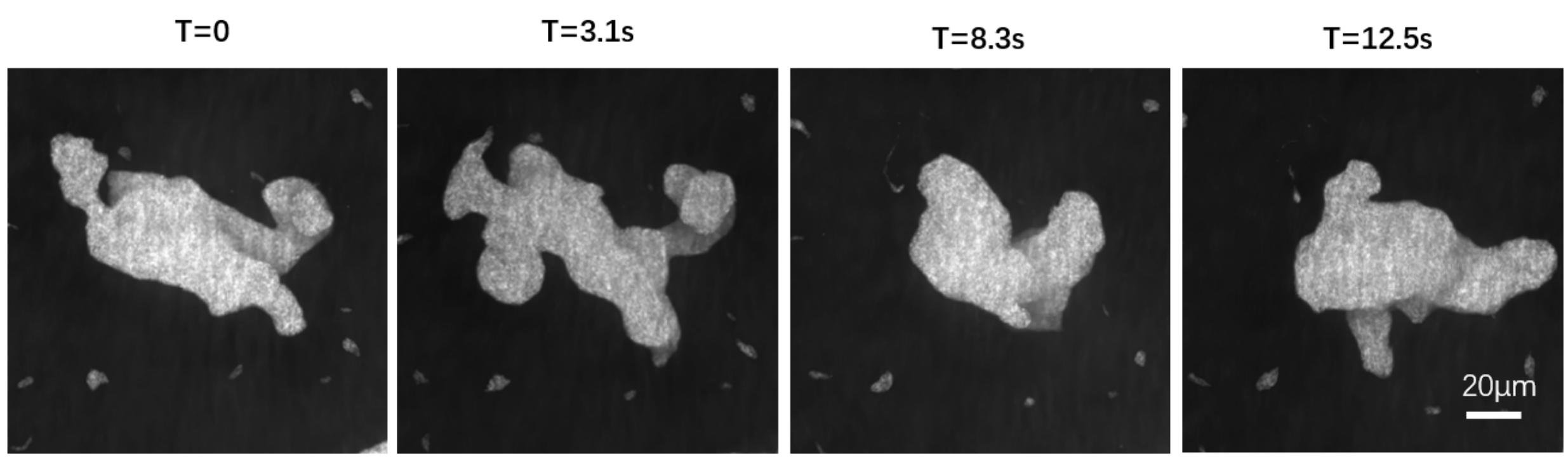

Supplementary Figure 33. Time series of a multi-protrusion droplet.

volume fraction among different droplets.

To obtain a clearer view of the bacteria inside such structures, we use the 240× SORA mode for imaging. As shown in Supplementary Figure 36, the bacteria inside the protrusions are locally aligned along the protrusion axis. We examine 11 bacteria from three different protrusions and find that the angle between the bacterial orientation and the local protrusion direction is typically $8 \pm 5°$, indicating clear local nematic order within the protrusions.

We suggest a possible scenario of the formation of these thin filaments: orientation fluctuations of bacteria near the interface may generate a local curvature perturbation, which

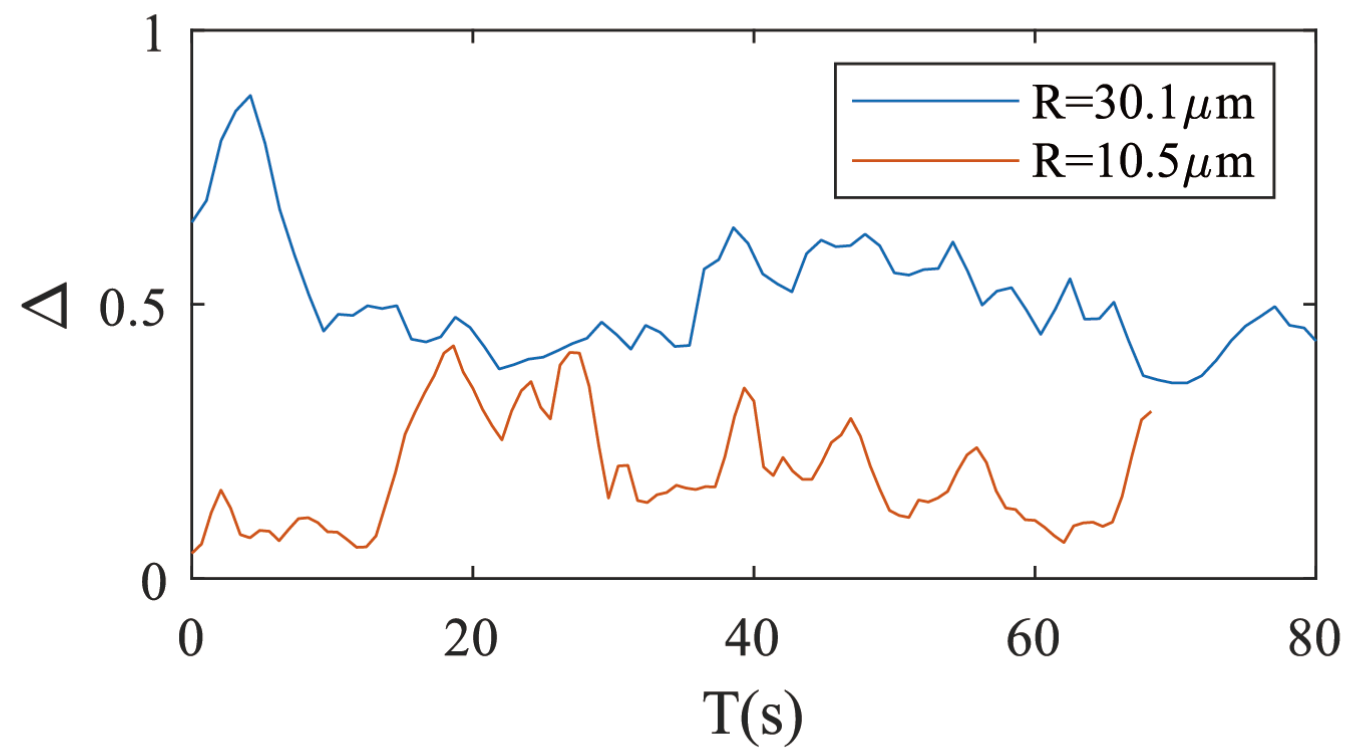

Supplementary Figure 34. The evolution of Δ of the two high-bacterial-density droplets with different sizes. The smaller droplet occasionally relaxed to a sphere with $\Delta \approx 0$, while the larger droplet remains in a deformed state $\Delta \approx 0.6$

could then favor further bacterial accumulation and alignment, thereby reinforcing protrusion growth.

C. Comparison of the two protrusive structures

The two classes of protrusive structures described above are compared quantitatively in Supplementary Figure 37. For the global elongated droplets, we analyze six droplets. For each case, we measure the minor axis of the best-fit ellipsoid obtained from the normalized second central moments at the time when the droplet aspect ratio exceeded π. The resulting width is approximately 20 μm, comparable to the velocity correlation length of the active turbulent flow. We manually measure five representative thin filaments, which exhibit a characteristic width of approximately 2 μm, comparable to a few bacterial body widths. This order-of-magnitude difference indicates that the two structures are associated with different physical length scales.

The global and local deformations discussed in Sec. S5A are therefore most naturally associated with coherent collective flows inside the droplet. Their width is comparable to the scale over which bacterial turbulent flows remain correlated, and their dynamics follow the persistence and reorganization of jet-like or vortical flow structures. This picture is reminiscent of the activity-capillarity competition discussed in simulations of active nematic

droplets [33], although our system is an experimental bacterial-turbulence droplet rather than an explicitly modeled active nematic continuum.

Thin filaments, in contrast, are bacterial-scale structures. Their width, together with the observed local alignment of bacteria along the filament axis, indicates that local confinement, packing, and orientational organization become important at this smaller scale. These filaments are distinct from membrane tubes observed in bacteria-filled vesicles [15, 34]: a membrane has bending elasticity and membrane tension that can stabilize slender tubular geometries, whereas our liquid–liquid interface has no bending rigidity.

Despite these differences, both structures should be viewed as transient nonequilibrium deformations of a liquid-liquid interface. They are sustained by active forcing from the bacterial suspension and opposed by capillary relaxation. They represent two distinct regimes of activity-driven interfacial deformation: one controlled primarily by collective-flow length scales, and the other by bacterial-scale confinement and local organization.

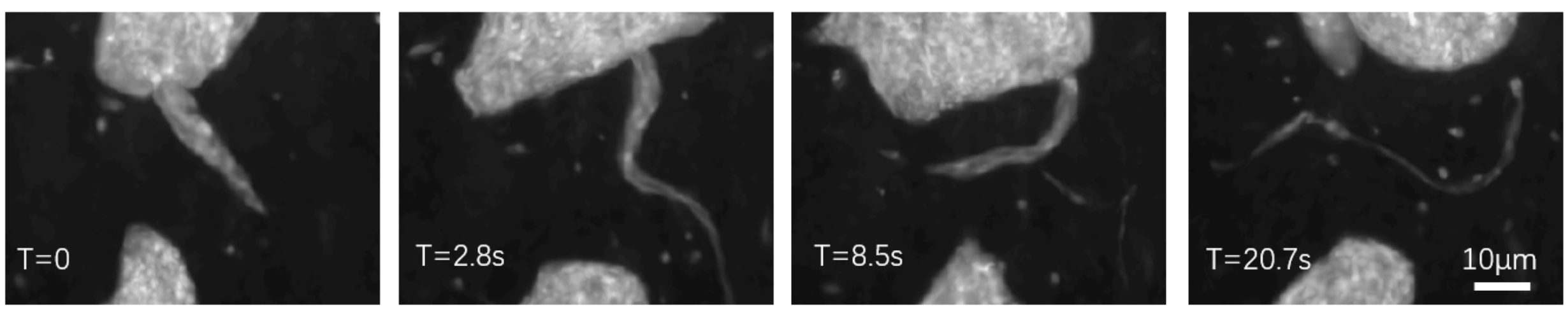

Supplementary Figure 35. The temporal snaps of a thin protrusion.

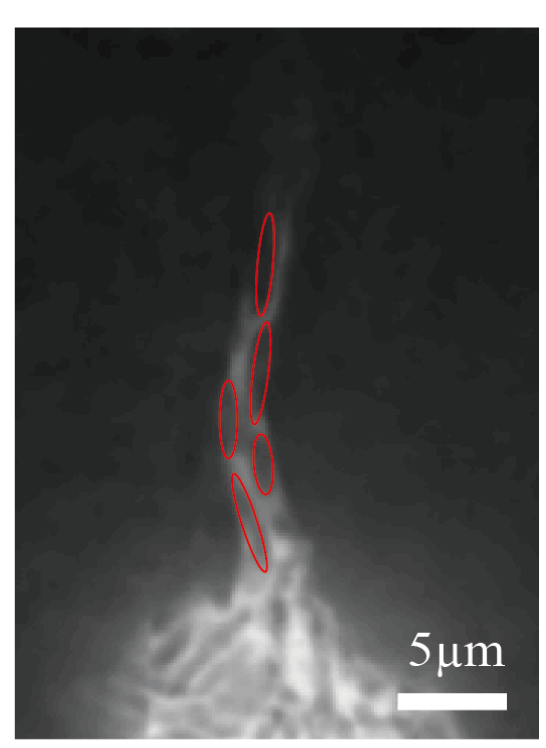

Supplementary Figure 36. A magnified thin filament. The red contours show the bacteria in the filament. Bacteria are oriented along the filament axis.

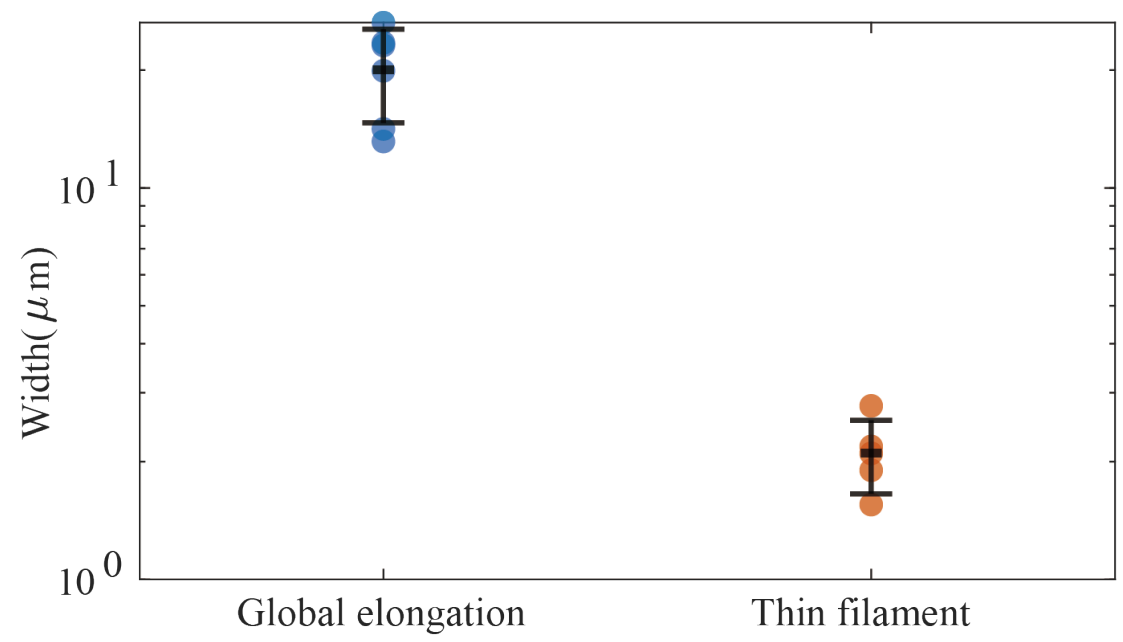

Supplementary Figure 37. Comparison of the characteristic thicknesses of two protrusive structures observed in high-bacterial density droplets.

Section S6. LIST OF SYMBOLS AND PARAMETERS

This section compiles all variables and symbols used throughout the study into a unified glossary. Definitions for geometric quantities, spectral measures, dynamical variables, and model parameters are provided for reference.

Symbol	Definition
$r(\varphi, t)$	Radial position of droplet interface at the equator at azimuthal angle φ and time t.
$r(\theta, \varphi, t)$	3D droplet interface shape in spherical coordinates.
R	Droplet radius.
$R_{\mathbf{eff}}$	Radius of a volume-equivalent sphere: $R_{\mathbf{eff}} = (3V/4\pi)^{1/3}$.
V	Droplet volume.
$\theta,\ \varphi$	Colatitude and longitude in spherical coordinates.
ϕ	Phase-field variable distinguishing droplet interior/exterior.
$C(\mathbf{r})$	The normalized autocorrelation function of the phase field ϕ.
L_c	The capillary length
k_B	Boltzmann constant
Φ_{Bulk}	The total volume fraction of droplets that do not touch the substrate.
φ_{b}	Bacterial volume fraction.
ξ	Characteristic length scale from coarsening analysis.
$\Delta = S/S_0 - 1$	Normalized excess surface area of droplets.
S, S_0	Droplet surface area and equilibrium spherical area.
k	Wave vector of deformation mode.
ω	Oscillation frequency of interfacial mode.
n_k	Wave number.
$S(k)$	Temporal average of interfacial fluctuation spectrum.
$\hat{u}_k$	Fourier component of radial deformation $r(\varphi, t)$.
$S(k, \omega)$	Dynamic structure factor of interfacial fluctuations.
$S_n(k, \omega)$	Dynamic structure factor normalized at each k.
$\tilde{u}_{k,\omega}$	Spatio–temporal Fourier component of deformation field.
ℓ_{v}	Correlation length calculated from $1/e$ of velocity correlation function of active turbulence.

Symbol	Definition
$Y_{\ell m}(\theta,\varphi)$	Spherical harmonic of degree ℓ and order m.
$\ell,\ m$	Angular (degree) and azimuthal (order) indices.
$P_\ell^m(\cdot)$	Associated Legendre function.
$f_{\ell m}(t)$	Time-dependent amplitude of spherical harmonic mode (ℓ, m).
A_ℓ	3D Fluctuation spectrum: $A_\ell = \langle \vert f_{\ell m}\vert^2\rangle_m$.
E_ℓ	Energy density associated with degree ℓ (m mode-averaged).
$n_{\ell n_k}$	Normalization factor mapping $(\ell, m = n_k)$ to equatorial Fourier mode k.
$P_{\ell n_k}(0)$	Associated Legendre function evaluated at $\cos\theta = 0$ (equator).
$h(x,t)$	Height field describing interface deformation in 2D geometry.
$\mathbf{v}$	Velocity field; v_y its vertical component.
v_0	Characteristic active speed in Toner–Tu–Swift–Hohenberg (TTSH) model.
a_ϕ, k_ϕ	Parameters in Cahn-Hillard model.
Γ_ϕ	Thermodynamic relaxation rate.
F_ϕ	Ginzburg-Landau free energy.
α	Effective damping coefficient in TTSH simulation.
S_v	A parameter related to the general hydrodynamics,
	which is positive for pusher swimmers in our simulations.
ν_1, ν_2	Effective viscosities of passive and active phases.
ν	Viscosity in TTSH simulation with $\nu = [\nu_1\,(1-\phi) + \nu_2\phi]$.
ζ	Fourth order viscosity coefficient in TTSH simulation.
ℓ_ϕ	Characteristic interface width $\ell_\phi = \sqrt{k_\phi/a_\phi}$.
τ_ϕ	Typical relaxation time $\tau_\phi = \Gamma_\phi a_\phi^2/k_\phi$.
ϵ_ϕ	Interfacial energy per unit length $\epsilon_\phi = \sqrt{a_\phi k_\phi}$.
λ_0	Advection coefficient in the fluid.
γ_0	Interfacial tension, defined as $\gamma_0 = \sqrt{2a_\phi k_\phi}/3$.
P	Pressure.
d	The dimension of the system.
μ	Chemical potential from Cahn–Hilliard free energy.
F^ϕ	Ginzburg–Landau free energy functional of ϕ.

**Symbol**	**Definition**
$\boldsymbol{\nabla}$	Gradient operator.
$\widetilde{\omega}$	Complex frequency, with $\Re(\widetilde{\omega}) = \omega$.
k_c	Critical wavenumber separating propagating and diffusive modes in experiments and simulations.
k_c'	Critical wavenumber separating propagating and diffusive modes given in theory.
τ_v	Viscous damping time of interface mode.
τ_0	Capillary oscillation period of interface mode.
$\nu_2'(k)$	Scale-dependent effective viscosity including higher-order damping and nonlinear dynamics.

[1] Schwarz-Linek, J. *et al.* Escherichia coli as a model active colloid: A practical introduction. *Colloids and Surfaces B: Biointerfaces* **137**, 2–16 (2016).

[2] del Río, O. I. & Neumann, A. W. Axisymmetric drop shape analysis: Computational methods for the measurement of interfacial properties from the shape and dimensions of pendant and sessile drops. *Journal of Colloid and Interface Science* **196**, 136–147 (1997).

[3] Lama, H., Yamamoto, M. J., Furuta, Y., Shimaya, T. & Takeuchi, K. A. Emergence of bacterial glass. *PNAS Nexus* **3**, pgae238 (2024).

[4] Yue, P., Feng, J. J., Liu, C. & Shen, J. A diffuse-interface method for simulating two-phase flows of complex fluids. *Journal of Fluid Mechanics* **515**, 293–317 (2004).

[5] Dunkel, J. *et al.* Fluid dynamics of bacterial turbulence. *Physical Review Letters* **110**, 228102 (2013).

[6] Dunkel, J., Heidenreich, S., Bär, M. & Goldstein, R. E. Minimal continuum theories of structure formation in dense active fluids. *New Journal of Physics* **15**, 045016 (2013).

[7] Heidenreich, S., Dunkel, J., Klapp, S. H. L. & Bär, M. Hydrodynamic length-scale selection in microswimmer suspensions. *Physical Review E* **94**, 020601 (2016).

[8] Reinken, H., Klapp, S. H. L., Bär, M. & Heidenreich, S. Derivation of a hydrodynamic theory for mesoscale dynamics in microswimmer suspensions. *Physical Review E* **97**, 022613 (2018).

[9] Gurtin, M. E., Polignone, D. & Vinals, J. Two-phase binary fluids and immiscible fluids described by an order parameter. *Mathematical Models and Methods in Applied Sciences* **6**, 815–831 (1996).

[10] Yue, P., Zhou, C., Feng, J. J., Ollivier-Gooch, C. F. & Hu, H. H. Phase-field simulations of interfacial dynamics in viscoelastic fluids using finite elements with adaptive meshing. *Journal of Computational Physics* **219**, 47–67 (2006).

[11] Kim, J. Phase-field models for multi-component fluid flows. *Communications in Computational Physics* **12**, 613–661 (2012).

[12] Yue, P., Feng, J. J., Liu, C. & Shen, J. A diffuse-interface method for simulating two-phase flows of complex fluids. *Journal of Fluid Mechanics* **515**, 293–317 (2004). Publisher: Cambridge University Press.

[13] Thibault, J. & Senocak, I. CUDA Implementation of a Navier-Stokes Solver on Multi-GPU Desktop Platforms for Incompressible Flows. In *47th AIAA Aerospace Sciences Meeting including The New Horizons Forum and Aerospace Exposition* (American Institute of Aeronautics and Astronautics, Orlando, Florida, 2009).

[14] Pécréaux, J., Döbereiner, H.-G., Prost, J., Joanny, J.-F. & Bassereau, P. Refined contour analysis of giant unilamellar vesicles. *The European Physical Journal E* **13**, 277–290 (2004).

[15] Takatori, S. C. & Sahu, A. Active contact forces drive nonequilibrium fluctuations in membrane vesicles. *Physical Review Letters* **124**, 158102 (2020).

[16] Shimobayashi, S. F., Konishi, K., Ackerman, P. J., Taniguchi, T. & Brangwynne, C. P. Critical capillary waves of biomolecular condensates. *bioRxiv* (2023). Preprint, not peer-reviewed.

[17] Vutukuri, H. R. *et al.* Active particles induce large shape deformations in giant lipid vesicles. *Nature* **586**, 52–56 (2020).

[18] Kokot, G., Faizi, H. A., Pradillo, G. E., Snezhko, A. & Vlahovska, P. M. Spontaneous self-propulsion and nonequilibrium shape fluctuations of a droplet enclosing active particles. *Communications Physics* **5**, 91 (2022).

[19] Park, M., Lee, K. & Granick, S. Response of vesicle shapes to dense inner active matter. *Soft Matter* **18**, 6419–6425 (2022).

[20] Sciortino, A. *et al.* Active membrane deformations of a minimal synthetic cell. *Nature Physics* **21**, 954–962 (2025).

[21] Adkins, R. *et al.* Dynamics of active liquid interfaces. *Science* **377**, 768–772 (2022).

[22] Faizi, H. A., Granek, R. & Vlahovska, P. M. Curvature fluctuations of fluid vesicles reveal hydrodynamic dissipation within the bilayer. *Proceedings of the National Academy of Sciences* **121**, e2413557121 (2024).

[23] Hadamard, J. S. Mouvement permanent lent d'une sphère liquide et visqueuse dans un liquide visqueux. *Comptes Rendus Hebdomadaires des Séances de l'Académie des Sciences* **152**, 1735–1738 (1911). In French.

[24] Zhao, L. *et al.* Asymmetric fluctuations and self-folding of active interfaces. *Proceedings of the National Academy of Sciences* **121**, e2410345121 (2024).

[25] Wioland, H., Woodhouse, F. G., Dunkel, J., Kessler, J. O. & Goldstein, R. E. Confinement Stabilizes a Bacterial Suspension into a Spiral Vortex. *Physical Review Letters* **110**, 268102 (2013).

[26] Lushi, E., Wioland, H. & Goldstein, R. E. Fluid flows created by swimming bacteria drive self-organization in confined suspensions. *Proceedings of the National Academy of Sciences of the United States of America* **111**, 9733–9738 (2014).

[27] Wei, D. *et al.* Scaling Transition of Active Turbulence from Two to Three Dimensions. *Advanced Science* **11**, 2402643 (2024).

[28] Grant, M. & Desai, R. C. Fluctuating hydrodynamics and capillary waves. *Physical Review A* **27**, 2577–2584 (1983).

[29] Harden, J. L., Pleiner, H. & Pincus, P. A. Hydrodynamic surface modes on concentrated polymer solutions and gels. *Journal of Chemical Physics* **94**, 5208–5221 (1991).

[30] Flekkøy, E. G. & Rothman, D. H. Fluctuating hydrodynamic interfaces: Theory and simulation. *Physical Review E* **53**, 1622–1643 (1996).

[31] Cross, M. C. & Hohenberg, P. C. Pattern formation outside of equilibrium. *Reviews of Modern Physics* **65**, 851–1112 (1993).

[32] Gulati, P., Caballero, F., Kolvin, I., You, Z. & Marchetti, M. C. Traveling waves at the surface of active liquid crystals. *Soft Matter* **20**, 7703–7714 (2024).

[33] Ruske, L. J. & Yeomans, J. M. Morphology of active deformable 3d droplets. *Physical Review X* **11**, 021001 (2021).

[34] Nagard, L. L. *et al.* Encapsulated bacteria deform lipid vesicles into flagellated swimmers. *Proceedings of the National Academy of Sciences* **119**, e2206096119 (2022).
