# Supplementary material for: Bacterial turbulence drives interfacial waves and shape dynamics in phase-separated droplets
Source: Nat Commun. 2026 Jul 14;17:8661. doi: 10.1038/s41467-026-75497-5 (PMC13490374; doi:10.1038/s41467-026-75497-5)
Supplement: Supplementary file 4 [file 41467_2026_75497_MOESM2_ESM.pdf]

Description of Additional Supplementary Files

Supplementary Movie Legends

File Name: Supplementary Movie 1

Description:

Confocal movies showing droplets coarsening under three bacterial density levels. Grayscales depict dextran-488 fluorescence, highlighting the dextran phase.

Top: passive coarsening without bacteria.

Middle: low-bacterial-density case, containing 200 μL PEG-rich liquid, 10 μL PEG-rich liquid, and 5 μL bacterial suspension, with $\varphi_{\rm b} = 21 \pm 4\%$.

Bottom: high-bacterial-density case, containing 200 μL PEG-rich liquid, 10 μL PEG-rich liquid, and 8 μL bacterial suspension, with $\varphi_{\rm b} = 34 \pm 3\%$.

The box size is 330 μm $\times$ 330 μm $\times$ 200 μm.

File Name: Supplementary Movie 2

Description:

Confocal movie of droplets with low bacterial density. The movie shows the top view of the droplet. Grayscales depict dextran-488 fluorescence, highlighting the dextran phase. The box size is 110 μm $\times$ 110 μm $\times$ 200 μm. The scale bar corresponds to the uppermost surface.

File Name: Supplementary Movie 3

Description:

Flow fields on the equator plane of two droplets with low activity in experiment and simulation.

Left-top: the flow field on the equator plane of a droplet with a radius of 39.6 μm in experiment.

Right-top: the flow field on the equator plane of a droplet with a radius of 273.5 μm in experiment.

Left-bottom: the flow field on the equator plane of a droplet with a radius of 12 simulated in a $48 \times 48 \times 48$ lattice.

Right-bottom: the flow field on the equator plane of a droplet with a radius of 32 simulated in a $128 \times 128 \times 128$ lattice.

In experiment, grayscales depict dextran-488 fluorescence, highlighting the dextran phase. In simulation, grayscales depict the intensity of the ϕ field, highlighting the active phase.

File Name: Supplementary Movie 4

Description:

Evolution of different initial perturbations on 3D droplets and 2D flat interfaces.

Supplementary Movie 4.1 shows the evolution of different ℓ-mode initialized perturbations, with $m = 0$, on 3D droplets. The left panel shows a droplet initialized with $\ell = 5$ and $m = 0$. The right

panel shows a droplet initialized with $\ell = 13$ and $m = 0$.

Supplementary Movie 4.2 shows the evolution of different k-mode initialized perturbations on 2D flat interfaces. The left panel shows a flat interface initialized with $n_k = 3$. The right panel shows a flat interface initialized with $n_k = 12$.

File Name: Supplementary Movie 5

Description:

Bright-field movie of high-bacterial-density droplets. The movie was recorded with a 10× objective on an inverted microscope.

File Name: Supplementary Movie 6

Description:

High-activity droplets with large aspect ratios in experiment and simulation.

Left-top: confocal images of a high-bacterial-density droplet from the top view. Grayscales depict dextran-488 fluorescence, highlighting the dextran phase. The box size is 67.3 μm$\times$67.3 μm$\times$54 μm. The scale bar corresponds to the length scale measured at the uppermost surface.

Right-top: 3D plot of a high-activity droplet in simulation. The box size is $128 \times 128 \times 128$.

Bottom: the aspect ratio of the droplets in experiment and simulation. The dotted line corresponds to the current time in the top two movies.

File Name: Supplementary Movie 7

Description:

High-bacterial-density droplets with topological changes. The movie shows the top view of the droplet. Grayscales depict dextran-488 fluorescence, highlighting the dextran phase. The box size is 201.9 μm × 201.9 μm × 58 μm. The red arrow highlights the emergence of a hole on the droplet surface, indicating topological changes. The scale bar corresponds to the uppermost surface.

File Name: Supplementary Movie 8

Description:

Thin filaments of droplets with high bacterial density. The movie shows the top view of the droplet. Grayscales depict dextran-488 fluorescence, highlighting the dextran phase. The box size is 110 μm × 110 μm × 56 μm. The red arrows highlight the formation of thin filaments. The scale bar corresponds to the uppermost surface.

File Name: Supplementary Movie 9

Description:

Side-view and contact-line dynamics of droplets at low and high bacterial densities. For each bacterial density level, the top panel shows the side view of the droplet, and the bottom panel shows the contact-line evolution. The green line in the bottom panel marks the instantaneous contact line.
